# Successes and challenges of primary health care in Australia: A scoping review and comparative analysis

**DOI:** 10.7189/jogh.13.04043

**Published:** 2023-07-30

**Authors:** Tesfaye S Mengistu, Resham Khatri, Daniel Erku, Yibeltal Assefa

**Affiliations:** 1 Bahir Dar University, College of Medicine and Health Sciences, School of Public Health, Bahir Dar, Ethiopia; 2 The University of Queensland, Faculty of Medicine, School of Public Health, Herston Queensland, Australia; 3 Centre for Applied Health Economics, Griffith University, Nathan, Queensland, Australia; 4 Menzies Health Institute Queensland, Griffith University, Gold Coast, Queensland, Australia; 5 Addis Consortium for Health Economics and Outcomes Research (AnCHOR)

## Abstract

**Introduction:**

Australia has achieved universal health insurance for its population since 1975 – a major step forward for increasing access to primary care (PC). Nevertheless, there are reports of several multi-layered challenges, including inequity, that persist. This analysis aims to undertake a scoping review of the success, explanatory factors, and challenges of Primary Health Care (PHC) in Australia guided by the World Health Organization (WHO)-defined key characteristics of good PC.

**Methods:**

We searched PubMed, Embase, Scopus and Web of Science using key terms related to PHC principles, attributes, system functioning and health care delivery modalities. We also used key PC terminologies used to assess key characteristics of good PC developed by WHO and key terms and attributes from Australia's health care landscape. We then integrated our search terms with the PHC Search Filters developed by Brown, L., et al. (2014). We restricted the search from 2013 to 2021. Two authors independently assessed study eligibility and performed a quality check on the extracted data. We presented findings according to the Preferred Reporting Items for Systematic Reviews and Meta-Analyses (PRISMA) guidelines.

**Results:**

We identified 112 articles on primary health care (PHC), represented from all Australian states and territories. Overall, Australian PHC has achieved comprehensiveness, access and coverage, quality of care, patient / person centeredness and service coordination indicators with exemplary evidence-base practice/knowledge translation and clinical decision-making practices at the PC settings. Yet, we identified complex and multilayered barriers including geographic and socio-economic berries and inequality, staff dissatisfaction/turn over, low adoption of person-centred care, inadequate sectoral collaboration, and inadequate infrastructure in rural and remote primary care units.

**Conclusion:**

Primary health care in Australia, which has evolved through major reforms, has been adapting to the complex health care needs of the socio-culturally diversified nation, and has achieved many of the PC attributes, including service diversity, accessibility, acceptability, and quality of care. Yet, there are persistent gaps in service delivery to socio-economically disadvantaged populations, including indigenous people, culturally and linguistically diverse (CALD) populations, and rural- and remote-residents. These challenges could be mitigated through system-wide and targeted policy-level intervention to further improve service delivery through effective and functional local health service coordination, sectoral integration, and improving health care providers’ cultural competence.

There is a sustained global commitment to achieve universal health coverage (UHC) through improving the coverage and quality of primary health care (PHC) [1-4]. Both low- and middle-income countries (LMICs) [5,6] and high-income countries (HICs) have designed and implemented PHC reform agendas to meet the changing health care needs of their nations. Establishing local or regional organisational structures, implementing new funding arrangements, and diversifying the PHC workforce skills mix are some of the strategies in the reform agendas that have been given particular emphasis [7-9].

In addition to achieving UHC and improving the quality of PC services, HICs aim to establish a strong and adaptable health care system. Like other HICs, Australia – with the rising burden of chronic disease and complex care needs [10], is developing its PC system to effectively tackle non-communicable diseases (NCDs) [11]. Australia has a strong and adaptable health system where its health care landscape is composed of four tiers- tier 1 “determinants of health”, tier 2 “health promotion and disease prevention”, tier 3 “primary health and community care”, tier 4 “specialist, acute and residential care”. PHC is the largest component constituting the first tier (determinants of health and other demographic factors), second tier (health promotion and disease prevention) and the third tier (primary health and community care) [12,13]. This implies that PHC is the gateway to access a broad range of health care services including disease prevention and health promotion, treatment and management of acute and chronic conditions [14,15]. Even though PHC is a vital component of Australia’s health care system, there is a persistent gap in addressing health equity and equality due to socioeconomic disadvantages, inadequate education, underemployment, racial prejudice, high-risk health-related behaviours and limited access to clinical services and health promotion programmes [16].

Limited availability of reliable and high-quality data has also constrained the extent of PHC implementation on the ground. Some studies show that effectiveness and health care equity have now been compromised [17,18]. However, to the knowledge of the authors, there is no comprehensive scoping review assessing PHC in Australia using the World Health Organization (WHO)’s eight key characteristics of good PC. Therefore, we sought to undertake a scoping review of the success, explanatory factors, and challenges of PHC in Australia.

## METHODS

### Search strategy

We performed a systematic search in major electronic databases including PubMed, Embase, Scopus and Web of Science using combinations of key terms related to the PHC principles, attributes, system functioning and health care delivery modalities. The eight key characteristics of good PC developed by WHO [19], presented in **Box 1** and key terms and attributes from Australia's health care landscape [12] were used to develop comprehensive search terms. We then integrated our search terms with the PHC Search Filters developed by Brown, L., et al. (2014) to facilitate easy and reliable access to the PHC literature [20].

Box 1 **WHO Key characteristics of good Primary Care (PC).**

While good service delivery is fundamental input to ensure population health status along with other factors, the organization and content of health services will differ from one country to another. But in any well-functioning health system, the network of service delivery should have the following key characteristics.

1. Comprehensiveness: a comprehensive range of health services is provided, appropriate to the needs of the target population, including preventative, curative, palliative and rehabilitative services, and health promotion activities.

2. Accessibility: services are directly and permanently accessible with no undue barriers of cost, language, culture, or geography. Health services are close to the people, with a routine point of entry to the service network at primary care level (not at the specialist or hospital level). Services may be provided in the home, the community, the workplace, or health facilities as appropriate.

3. Coverage: service delivery is designed so that all people in a defined target population are covered, ie, the sick and the healthy, all income groups and all social groups.

4. Continuity: service delivery is organized to provide an individual with continuity of care across the network of services, health conditions, levels of care, and over the life cycle.

5. Quality: health services are of high quality, ie, they are effective, safe, centred on the patient’s needs and given in a timely fashion.

6. Person-centeredness: services are organized around the person, not the disease or the financing. Users perceive health services to be responsive and acceptable to them. There is participation from the target population in service delivery design and assessment. People are partners in their own health care.

7. Coordination: local area health service networks are actively coordinated, across types of providers, types of care, levels of service delivery, and for both routine and emergency preparedness. The patient’s primary care provider facilitates the route through the needed services and works in collaboration with other levels and types of providers. Coordination also takes place with other sectors (eg, social services) and partners (eg, community organizations).

8. Accountability and efficiency: health services are well managed to achieve the core elements described above with a minimum wastage of resources. Managers are allocated the necessary authority to achieve planned objectives and held accountable for overall performance and results. Assessment includes appropriate mechanisms for the participation of the target population and civil society.

We searched research works from 2013 to 2021 as this period the hallmark in Australian PHC where the National Primary Health Care Strategic Framework was developed in 2013 [21] and covers phase 3 (build and communicate an evidence base in PHC) of PHC Research, Evaluation and Development (PHCRED) Strategy [22]. The search terms and or / free text concepts were combined using the Boolean connectors “AND” and “OR” and the search strategies were adapted to the specific electronic databases by modifying field codes. We included only studies published in English, conducted in Australia, and meeting the following criteria: (1) original studies of any design, (2) conducted on the subject area of PHC regardless of the health care professional categories involved.

We excluded systematic review / meta-analysis, methodology papers / protocols, global studies / workshops / seminars / conference papers, case studies / case-series, pilot studies / tool validation studies and studies with Australian affiliation but not actually conducted in Australia. TSM and YAA developed search terms, strategies, and filters; and TSM conducted database searches accordingly to the agreed search strategy. The search terms, search strategies for each electronic database and filters are presented in File 1 in the **Online Supplementary Document**.

### Study selection

We imported retrieved papers into EndNote X20 and removed duplicates. TSM screened the titles and abstracts. When the papers had insufficient information in the title and / or abstract during the screening process, a full text was red and TSM generated a final list of eligible papers. YA re-screened the final list of papers deemed eligible for full-text review independently. We resolved disagreements through discussions.

### Data extraction and analysis

Two reviewers (TSM and YA) checked the lists of selected articles and TSM extracted data on study characteristics (author, year of publication, jurisdictions (states and territories), research focus duration of the study, study type / design, study participants, study sample, key findings, conclusions, and the WHO key characteristics of PC assessed / addressed in the study) using data extraction sheet. YA checked the content completeness, accuracy, and quality of extracted data. Data were thematically synthesized and narrated using the key characteristics of PC. Achievements, explanatory factors, and challenges / barriers of PHC service are presented. This scoping review was conducted and reported according to the Preferred Reporting Items for Systematic Reviews and Meta-Analyses (PRISMA) guidelines [23].

The study is guided by the eight key characteristics of good PC delivery as defined by the WHO [19] (**Box 1**).

## RESULTS

**Figure 1:**
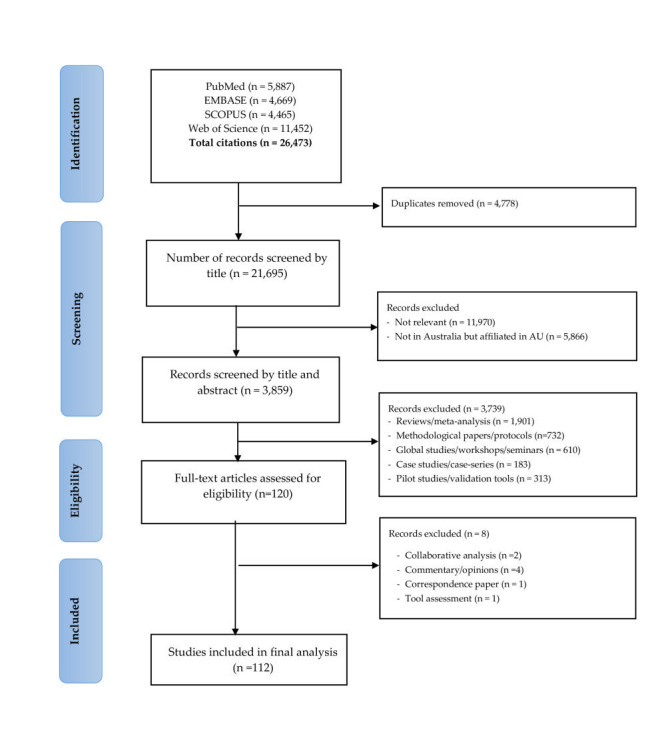
PRISMA flow diagram of screening and selection process.

**Figure 1** describes the process of study screening, selection, and reasons for exclusion. The initial search identified 26 473 studies with 4778 duplicates. After removing duplicates, we screened 21 695 studies by title and 3859 were selected for further abstract screening. Of these, 120 original studies were eligible for full-text review. Finally, a total of 112 studies were included in the final analysis (**Figure 1**).

### Characteristics of included studies

The overall characteristics of included studies are summarized in each section of the key attributes of good PHC delivery [19]. Of 112 studies included, methodologically, 29 were qualitative studies [17,24-51], 20 were cross-sectional [52-71], 15 were mixed–method studies [72-86], 10 were national / state based surveys / census [87-96], nine were retrospective / prospective cohort [97-105], eight were interventional / experimental (pre-post) studies [106-113], four were randomised / non-randomised controlled trials [114-117], four were evaluation studies [118-121], three were population-based studies [64,122,123], three were longitudinal studies [124-126], two were ecological studies [127,128] and one was group stepped-wedge trial [129]. Four studies did not clearly mention the methods used [130-133].

### Key characteristics of studies, success, and challenges of PHC in Australia

#### Comprehensiveness

Comprehensive PHC is characterised by the provision of a full ranges of appropriate services and meeting clients’ health care needs [19,134]. In our analysis, the Australian PHC delivery is comprehensive that it provides a wide range of services for a variety of target populations. In terms of the ranges of services, Australian PHC assess and deal with social determinants and / or risk factors [17,24,25,30,36,45,48,53,62,63,65,90,98,99,125], acute and chronic diseases [27,47,52,59,81,82,84,96,97,101,104,105,108,110,111,114,117,118,120], mental health [26,46,61,68,126,132], cancer and palliative care including aged care [43,86,103,122], pharmacy and medication safety [35,107], evidence-based practice and knowledge translation [55,106], and other multi-disciplinary, sexual and refugee health issues [51,77,119,130]. Increasing number and skill mix of health human resource [7-9,33,38,42,44,58,66,78,80,88,91,92,94) (Table S5 in the **Online Supplementary Document**), improved health care acceptability [53,90], accessibility [72,93,128], ever-increasing health care need / use and service provision [28,29,31,34,37,39-41,60,67,70,73,76,87,89,112,116] (Table S7 in the **Online Supplementary Document**) might have contributed to the overall comprehensiveness of the PHC services.

While improved service access [53,65], sectors coordination [24,37,40,76,87,126], service integration [31,39,41,46,51,69,118] and effective health reforms [55,69] implemented so far also contributed to the comprehensiveness of PHC service delivery, health care cost / economic barriers [131,133], geographic barriers [72,93,128], lack of staffing and physical resources in remote and very remote PHC services that created stress and dissatisfaction in staffs [42], staff turnover [58] and low level of interest among new graduates to work in PHC settings [91] are barriers to ensure further comprehensiveness. While the financial incentive to PHC providers plays a limited role in improving access [88], dissatisfaction among PHC service providers was associated with a higher intention of experienced staffs to leave their role [92].

#### Access and coverage

Given that PHC is a means to improve access to health services towards UHC [14,15], in our scoping review, 36 studies [17,25,30,34,36,44,45,50,53,54,61,65,67,71,72,74,75,79,83,85,90,93,95,96,98-102,116,122,127,128,131-133] assessed various indicators of access to PHC. Of these studies, 15 studies reported service access [36,45,53,54,71,72,74,83,85,90,99,100,102,122,132), three reported acceptability [44,65,75] and the rest of the studies assessed explanatory factors, barriers and challenges of access and coverage of PHC [17,25,30,34,50,61,67,79,93,95,96,98,101,116,127,128,131,133] (Table S1 in the **Online Supplementary Document**). The current study shows that there is a significantly improved overall health care access in Australia. This success could be partly explained by the observed increase in the acceptability of PHC over several years. For example, the acceptability of preventive care ranges from 76%-97% for diverse services and or diseases [53,65]. While the overall access and coverage to PHC showed tremendous achievements, a sizeable population with different socio-demographic and geographic characteristics have less access to preventive, curative and rehabilitative services. Low preventive risk behaviours [53,90], being social disadvantage [36,128], cost and financial barriers [61,95,96,101,131,133], disparity (equity / inequality) [17,25,34,127), regional / geography variation [61,67,93,128] remained major barriers to access PHC. Studies also reported that cultural respect at the PHC level shows a positive result in addressing social determinants of health [30,98]. However, communication barriers, racial discrimination [30], and low level of health professionals’ social connectedness with indigenous people’s culture [50,98] are some of the barriers to addressing social determinants of health and improving access to PHC services (Table S1 in the **Online Supplementary Document**). Physical resources and health human resource factors are also key factors explaining low access to PHC. In our analysis, there is a perceived lack of staffing and physical resources [42], staff dissatisfaction and turnover [42,58,92] affecting PHC access and coverage. Due to the existing gaps to ensure access and coverage, studies have also reported that remote and disadvantaged communities have low PHC coverage and health service (Table S7 in the **Online Supplementary Document**).

#### Quality of care

Table S2 in the **Online Supplementary Document** describes characteristics of 15 studies [26,35,52,60,82,105,107,112,113,115,117,123,124,129,130] conducted on quality of care and its attributes (including continuity of care, effectiveness, and safety). Of these studies, seven reported quality of care [26,52,82,105,112,113,124], three described the effectiveness of different interventions to ensure the quality of care [117,129,130] and two studies reported continuum of care [60,115]. There are several factors including client or system factors [52,105] affecting the quality of care. Yet, the attributes and factors affecting quality of care are neither consistent across PHC settings nor easily measurable [124]. Some studies also reported that there is limited evidence on quality of PC [105]. However, regardless of the measurement challenges and varying trends in quality of care [124], our review shows that the Australian PHC quality meets good quality indicators [26,52]. The effective implementation of the primary health networks (PHNs) strategy could be one of the major reasons to improve the quality care of PC [29,117,129,130]. The emphasis to improve the number and skill mix of health professionals working in PHC setting [7-9], interprofessional teamwork [78], efforts to ensure continuity of care continuum of care [60,115], and health professional role as cultural educators [80], improved physical infrastructure could be the explanatory factors for improved quality of care. On the other hand, the improved use of evidence-based practice and knowledge translation was a crucial factor in improved quality care. In this review, we observed that the Australian PHC system has an exemplary system for the effective implementation of evidence practice, and knowledge translation at PHC settings [55,56,114,121]. In our review, evidence practice and / or knowledge translation in PHC settings are associated with improved patient care [56,106]. Albeit there are gaps, improved pharmacy practice and existing policy emphasis on medication safety [35,107] are also linked with improved quality of care. However, the dynamic and ever-increasing complex health care needs, health professionals’ dissatisfaction [58,92], and staff turnover remain important challenge to sustaining health care quality standards [88,92,94]. Studies also reported the effect of low interagency linkage [26], staff turnover [105] and discrimination [123] on quality of care. Additionally, challenges of implementing evidence-based practice / knowledge translation such as lack of genuine engagement, limited perceived importance, limited access to, or familiarity with the resources and clarity of responsibilities in knowledge translation [55,56,114,121] might affect the quality of care provided (Table S6 in the **Online Supplementary Document**).

#### Person / patient-centeredness

Table S3 in the **Online Supplementary Document** presents characteristics of nine studies that assessed the person / patient-centredness of PHC services [33,47,59,97,108-111,120]. In this review, we observed that clients show strong interest in receiving person-centred health care services and the services were found to be correlated with improved quality of life for patients with chronic diseases [97,108-111,120] and overall health outcomes or self-management of diseases conditions [33,59,110,120]. The possible reason for this link could be due to the fact that patient – centred PC supports patients to be engaged in their care process [110]. In the current review, the overall PC service use has improved over the last decade [33,53,59,65,110,120] and there is a progressive implementation of the person / client-centred PC [135,136] that ensure quality and acceptability of care further. However, low adoption rate, lack of diversity recognition, limited health care providers’ cultural competence [33] and infrastructure particularly in the remote indigenous population [47] are major challenges in implementing person / patient-centred PC service. This implies that prioritisation and a significant level of engagement are required to improve person-centred PC [59] (Table S3 in the **Online Supplementary Document**). With the tremendous improvements in PHC service, the health services use rate in rural communities such as mental health, is a bit over the national rate [68]. These advances might be due to the increased use of evidence based-practices and person-centredness of services. Because the use of evidence to support advanced clinical and community health care impacts health outcomes positively [56,114]. Studies have also shown that reliance on the medical model [64], low health literacy [63] and infrastructure [89] are impediments to patient-centred service sustainability (Table S7 in the **Online Supplementary Document**).

#### Service coordination / multi-stakeholder collaboration

There were 24 studies [24,27-29,31,32,37,39-41,46,48,49,51,57,64,69,76,77,81,87,118,119,126) that reported coordination/integration, stakeholder engagement or intersectoral collaboration (Table S4 in the **Online Supplementary Document**). Of these studies, 13 studies assessed service coordination / integration [24,31,37,39-41,46,51,69,76,87,118,126], six studies reported stakeholder engagement / collaboration [27,28,48,57,81,87], two studies each reported community participation [76,119], multi-agency / multi-faceted communication [29,64] and intersectoral / cross-sectoral coordination [32,49]. Service coordination and effective referral links in PHC are crucial if a patient is to receive continued quality care. In our study, although health service acceptability (86%-97%) [44,65,75], and service use [63,64,68,70,89,103,104,125] (Table S7 in the **Online Supplementary Document**) have increased over time, the number of referrals for behavioural risks for chronic diseases was not increased [129]. Similarly, a study reported that only 38%-49% of chronic disease patients received a referral for their complex health care needs including mental health [53]. Similarly, Harris et al. [114] reported that only one in six patients received a referral for chronic disease risks. Factors such as distance between the source and referral service [64] and health literacy [63] and high out-of-pocket costs are some of the barriers to effective service coordination via referral. Our review also shows that health care coordination is crucial to target resources [37], assess the intersectoral view of PHC [49] and address multiple and complex health care needs [126] through community participation and cultural literacy [119]. Yet, several challenges – including communication / language barriers, limited health literacy, cost [37], infrastructure and territorialism [39] affect PHC service coordination / integration. Given that service integration or inter-sectoral coordination has a synergistic effect in PHC service [81], service coordination / integration or transitioning care for the disadvantaged population such as refugees, prisoners [37,51,77] and indigenous populations [64] needs critical policy and programmatic interventions (Table S4 in the **Online Supplementary Document**). The innovative community discussion model – YARN – an Indigenous cultural form of conversation that creates safe space for discussion and debate - is effective to identify social determinants of health [24] and other barriers [119].

**Figure 2:**
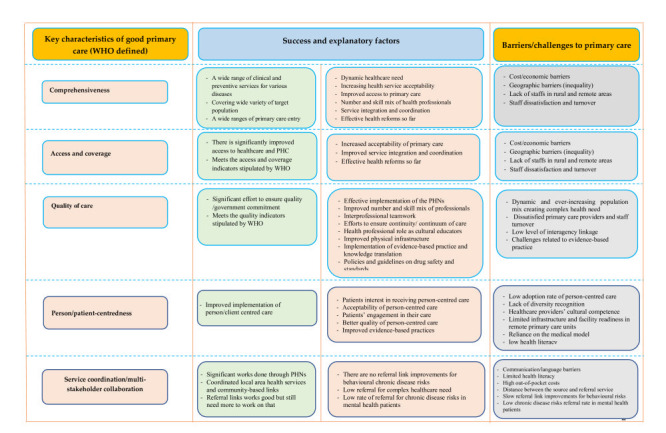
Success, challenges, and explanatory factors in Australian primary health care (PHC).

The overall of success and explanatory factor, and barriers / challenges for the primary health care in Australia are summarised according to the WHO key characteristics of good primary care in the following framework (**Figure 2**).

## DISCUSSION

In this review, we found that Australian PC has achieved most of the good characteristics of a PC delivery. Yet there are persistent, emerging, and re-emerging challenges and barriers either affecting to achieve more successes or sustain the ones that have been accomplished over many years of health sector reforms.

There is substantial evidence that achieving UHC, meeting diversified and dynamic health care needs [19,134] and ensuring quality of PHC have many trade-offs [137]. While there are many strategies to achieve many of the PHC goals and mitigate major bottlenecks, ensuring service comprehensiveness is an important means to minimise major trade-offs. Increasing service diversification – comprehensiveness helps to provide a full range of PC [3,115] and it is also a means to achieve UHC and service access. Given that the Australian health care system has gone through a number of reforms [29,55,87], our findings – like previous studies [25,29] show that it provides a wide range of services and meets most WHO indicators [19]. While there might be several success factors for the provision of comprehensive PC, consecutive health system reforms [29,55,87], continued government commitment [29,55,69,87] and effective implementation of the current PHNs to provide culturally informed holistic PHC that address social and structural determinants of health [25]. Yet, being socially disadvantaged or racial discrimination [30,36,50,98,116,128], cost and financial barriers [61,95,96,101,131,133], regional / geographic variations [61,67,93,128], health disparity (equity / inequality) [17,25,34,127], communication barriers and lack of social connectedness with indigenous people’s culture [50,98] are persistent challenges of accessibility, acceptability, and quality of PHC. Strengthening integrated, accessible, quality and culturally appropriate care for disadvantaged, culturally and linguistically diverse, Aboriginal and Torres Strait Islander people should be priority.

In line with WHO recommendations [19], this study shows that there is a good quality of PC. These achievements could be due to adaptive and need-driven health care reforms so far [29,55,87], the due emphasis on preventive care with good behavioural risk assessment [117,129,130], improved person-centredness of PC [1,4,17,24,76,138] and good evidence-based practice at the PC settings [56,106]. However, there is a wide range of variations in the quality of PHC care [52] which may be linked with a lack of effective interagency linkage [26], low socio-economic status [30,36,50,98,116,128], geographic barriers [61,67,93,128], cultural, and racial discrimination [30,36,123,128]. This implies that the implementation of continuous quality improvement (CQI) [82,112,124] should be further aligned with the identified barriers. Still, the implementation should consider the fact that data on PHC quality are limited [105] or neither consistent across settings nor easily measurable [124].

In addition to previous factors, lack of staffing / retention and staff turnover [105] are also gaps in remote and very remote PHC settings [42] that might have compromised the effort to provide person-centred quality care. In our study, there is significantly low interest among new graduates to take PHC roles [91] which may fuel the existing workforce shortage. This implies that integrated system thinking approaches are needed to motivate and retain primary health care practitioners and ensure the quality of care through providing person-centred care in addition to the less effective financial incentives [58,88,92]. In accordance with previous studies [32,49,76,119,139], this study also shows that local area health service integration, functional intersectoral coordination and community engagement are important to retain and integrate health professionals [24], target resources [37] and assess intersectoral understanding of primary health care [49].

It is reported that the Australian primary health care reforms are adaptive to the dynamic health care needs of a diverse population and emphasized the role of community governance / ownership and multisectoral collaboration to provide accessible, quality, responsive, person-centred or population health care [17,76,138]. Yet, the ever-increasing population diversity, increasing risk factors, increasing chronic diseases and disease co-morbidity [10], limited health literacy, the financial cost [37], infrastructure and territorialism [39] are existing challenges to implementing principled equitable and integrated / coordinated primary health care. Hence, investigating the level of service integration using a service integration matrix “user need vs level of integration” [139] could be effective to identify multiple and complex challenges to integrate primary health care [126]. In this scoping review – despite a recent federal government commitment to close the Indigenous and geographic health care gaps by 2030 [16], we also found that there is a persisted health care gap in the Aboriginal population [57,64,80]. Similarly, inequitable primary health care also contributed to the poorer health status of rural and remote Australians compared to many metropolitan residents [140]. Local health networks and primary health networks should further map identified challenges and barriers to meet the national PHC targets.

The current scoping review shows that the overall PHC service use has improved over the last decade [33,53,59,65,110,120] and there is a progressive implementation of a person / client-centred PHC to ensure the quality and acceptability of care [135,136]. Conversely, studies also show that person–centred PC is still reliant on the medical model and fee for service financing which is focused on the diseases rather than the client / person [64]. This could be due to the low adoption rate of the person / patient centred PC observed in our scoping review [59]. Lack of diversity recognition, limited health care providers’ cultural competence [33] and limited infrastructure in remote and very remote PHC settings [47] could have contributed to the low rate of adoption and implementation of a person / patient-centred PHC service. Whilst the WHO did not mention evidence / knowledge translation as a measure of good characteristics of PHC, it indicates that evidence translation, research, and dialogue in the early stage [19] are important. In this review, we observed that knowledge / evidence translation research in Australian PHC settings is progressive and further multimodal knowledge / evidence translation with effective change communication and familiarisation, and sectoral engagement [55,56,114,121] could ensure effective implementation.

The comprehensive nature of this scoping review and our comparison of the Australian PHC attributes with the WHO-defined key characteristics of good PC are the main strengths of this scoping systematic review. This scoping review included quantitative and qualitative studies from all states and territories with adequate mix of methods. However, this study did not include studies published before 2013. Our exclusion of unpublished studies could also be one of the limitations. This study is also unable to capture adequate numbers of studies to map the continuity of care and accountability in PC settings. In our review, although we speculated that some of the barriers are more relevant to the socio-economically disadvantaged and rural population, the reasons for the barriers and sub-group narrative analyses were beyond the scope of this study. Australia revised and re-endorsed its PHC Policy Position Statement since 2004 with most recent revision in 2020 [141] leading to the current 10-year PHC policy [142]. In these policy documents [141,142] and other reports [143], PHC is broader and encompasses PC. Hence, we used the combination of those concepts, and the readers are advised to make cautious interpretation and conclusion.

## CONCLUSION

Australia has given due emphasis to narrowing the persistent health service and health status gaps among populations and locations. Primary health care in Australia evolved through major reforms to adapt to the dynamic and complex health care needs of its socio-culturally diversified society. The PHC system has developed several implementation strategies to increase service diversity, accessibility, acceptability, and quality of care. Findings in this scoping review ascertained that the Australian PHC meets most of the WHO key characteristics of good PC. However, existing multi-level factors including regional / geography inequality, being socially disadvantaged (Indigenous population), communication barriers, racial discrimination, increasing cost, and financial barriers remain challenges of PHC, which affect accessibility, affordability, acceptability, and quality of care. Furthermore, lack of effective and functional service integration, lack of diversity recognition, limited health care providers’ cultural competence, staff turnover, and health professionals’ dissatisfaction also affect the implementation of a person / client-centred care to the ensure quality and acceptability of PHC service. In addition, high-level sectoral integration and community engagement is required.

The practice of knowledge / evidence translation in clinical decision-making practices in Australian PHC settings plays a key role to ensure quality of care and person-centred health care. This shows that PHC in Australia has this advantage over the WHO-recommended characteristics of good PC. Yet, there is a paucity of evidence on accountability in PHC service which warrants further investigation with other key important PHC characteristics. This study suggests the need to define core or essential PHC services to further facilitate research and assessment of PHC implementation in Australia.

## Additional material


Online Supplementary Document

## References

[1] Sacks E Schleiff M Were M Chowdhury AM Perry HB Communities, universal health coverage and primary health care. Bull World Health Organ. 2020;98:773-80. 10.2471/BLT.20.252445 33177774 PMC7607457

[2] World Health Organization (WHO). Primary health care on the road to universal health coverage: 2019 global monitoring report. 2021. Report No.: 9240004270.

[3] Schwarz D Hirschhorn LR Kim J-H Ratcliffe HL Bitton A Continuity in primary care: a critical but neglected component for achieving high-quality universal health coverage. BMJ Glob Health. 2019;4:e001435. 10.1136/bmjgh-2019-001435 31263586 PMC6570977

[4] Sanders D Nandi S Labonté R Vance C Van Damme W From primary health care to universal health coverage—one step forward and two steps back. Lancet. 2019;394:619-21. 10.1016/S0140-6736(19)31831-8 31448726

[5] Assefa Y Tesfaye D Van Damme W Hill PS Effectiveness and sustainability of a diagonal investment approach to strengthen the primary health-care system in Ethiopia. Lancet. 2018;392:1473-81. 10.1016/S0140-6736(18)32215-3 30343861

[6] Binagwaho A Ghebreyesus TA Primary healthcare is cornerstone of universal health coverage. BMJ. 2019;365:l2391. 10.1136/bmj.l2391 31160322

[7] Gargano G The Bottom-Up Development Model as a Governance Instrument for the Rural Areas. The Cases of Four Local Action Groups (LAGs) in the United Kingdom and in Italy. Sustainability. 2021;13:9123. 10.3390/su13169123

[8] McDonald J, Cumming J, Harris M, Powell Davies G, Burns P. Systematic review of comprehensive primary health care models. 2017.

[9] Organization WH. Primary health care in the Western Pacific Region: looking back and future directions. 2018.

[10] Fisher M Freeman T Mackean T Friel S Baum F Universal health coverage for non-communicable diseases and health equity: lessons from Australian primary healthcare. Int J Health Policy Manag. 2022;11:690-700. 33300769 10.34172/ijhpm.2020.232 PMC9309940

[11] Simpson SJ Saint V Bozorgmehr K Changing the Discourse in Ambitions Towards Universal Health Coverage: Lessons From Australian Primary Healthcare Comment on “Universal Health Coverage for Non-communicable Diseases and Health Equity: Lessons From Australian Primary Healthcare”. Int J Health Policy Manag. 2022;11:851-4. 34973056 10.34172/ijhpm.2021.165 PMC9309900

[12] Australian Government Department of Health. Australia's health landscape infographic. 2017.

[13] Australian Institute of Health Welfare. Primary health care. Canberra: AIHW; 2020.

[14] Australian Institute of Health Welfare. Australia's health 2016. Canberra: AIHW; 2016.

[15] Australian Institute of Health Welfare. Primary health care in Australia. Canberra: AIHW; 2016.

[16] Gracey M Why closing the Aboriginal health gap is so elusive. Intern Med J. 2014;44:1141-3. 10.1111/imj.12577 25367729

[17] Freeman T Baum F Lawless A Javanparast S Jolley G Labonté R Revisiting the ability of Australian primary healthcare services to respond to health inequity. Aust J Prim Health. 2016;22:332-8. 10.1071/PY14180 28442028

[18] Bailie J Potts BA Laycock AF Abimbola S Bailie RS Cunningham FC Collaboration and knowledge generation in an 18-year quality improvement research programme in Australian Indigenous primary healthcare: a coauthorship network analysis. BMJ Open. 2021;11:e045101. 10.1136/bmjopen-2020-045101 33958341 PMC8103942

[19] World Health O. Monitoring the building blocks of health systems: a handbook of indicators and their measurement strategies. Geneva: World Health Organization; 2010 2010.

[20] Brown L Carne A Bywood P McIntyre E Damarell R Lawrence M Facilitating access to evidence: Primary Health Care Search Filter. Health Info Libr J. 2014;31:293-302. 10.1111/hir.12087 25411047

[21] Health SCo. National primary health care strategic framework. 2013.

[22] Dijkmans-Hadley B Bonney A Barnett SR Development of an Australian practice-based research network as a community of practice. Aust J Prim Health. 2015;21:373-8. 10.1071/PY14099 25738866

[23] Moher D Liberati A Tetzlaff J Altman DG Preferred reporting items for systematic reviews and meta-analyses: the PRISMA statement. PLoS Med. 2009;6:e1000097. 10.1371/journal.pmed.1000097 19621072 PMC2707599

[24] Baum FE Legge DG Freeman T Lawless A Labonté R Jolley GM The potential for multi-disciplinary primary health care services to take action on the social determinants of health: actions and constraints. BMC Public Health. 2013;13:460. 10.1186/1471-2458-13-460 23663304 PMC3660265

[25] Pearson O Schwartzkopff K Dawson A Hagger C Karagi A Davy C Aboriginal community controlled health organisations address health equity through action on the social determinants of health of Aboriginal and Torres Strait Islander peoples in Australia. BMC Public Health. 2020;20:1859. 10.1186/s12889-020-09943-4 33276747 PMC7716440

[26] Bassilios B Nicholas A Ftanou M Fletcher J Reifels L King K Implementing a Primary Mental Health Service for Children: Administrator and Provider Perspectives. J Child Fam Stud. 2017;26:497-510. 10.1007/s10826-016-0572-9

[27] Davy C Cass A Brady J DeVries J Fewquandie B Ingram S Facilitating engagement through strong relationships between primary healthcare and Aboriginal and Torres Strait Islander peoples. Aust N Z J Public Health. 2016;40:535-41. 10.1111/1753-6405.12553 27523395

[28] Durey A McEvoy S Swift-Otero V Taylor K Katzenellenbogen J Bessarab D Improving healthcare for Aboriginal Australians through effective engagement between community and health services. BMC Health Serv Res. 2016;16:224. 10.1186/s12913-016-1497-0 27388224 PMC4936288

[29] Abou Elnour A Dunbar J Ford D Dawda P General practices’ perspectives on medicare locals’ performance are critical lessons for the success of primary health networks. Australas Med J. 2015;8:320-4. 10.4066/AMJ.2015.2508 26576203 PMC4643609

[30] Freeman T Edwards T Baum F Lawless A Jolley G Javanparast S Cultural respect strategies in Australian Aboriginal primary health care services: beyond education and training of practitioners. Aust N Z J Public Health. 2014;38:355-61. 10.1111/1753-6405.12231 25091076

[31] Grace S Bradbury J Avila C Du Chesne A ‘The healthcare system is not designed around my needs’: How healthcare consumers self-integrate conventional and complementary healthcare services. Complement Ther Clin Pract. 2018;32:151-6. 10.1016/j.ctcp.2018.06.009 30057043

[32] Green A Abbott P Luckett T Davidson PM Delaney J Delaney P Collaborating across sectors to provide early intervention for Aboriginal and Torres Strait Islander children with disability and their families: a qualitative study of provider perspectives. J Interprof Care. 2020;34:388-99. 10.1080/13561820.2019.1692798 31821054

[33] Harrison R Walton M Chauhan A Manias E Chitkara U Latanik M What is the role of cultural competence in ethnic minority consumer engagement? An analysis in community healthcare. Int J Equity Health. 2019;18:191. 10.1186/s12939-019-1104-1 31801565 PMC6894316

[34] Henderson J Javanparast S MacKean T Freeman T Baum F Ziersch A Commissioning and equity in primary care in Australia: Views from Primary Health Networks. Health Soc Care Community. 2018;26:80-9. 10.1111/hsc.12464 28608451

[35] Hermansyah A Sainsbury E Krass I Investigating influences on current community pharmacy practice at micro, meso, and macro levels. Research in social & administrative pharmacy. Res Social Adm Pharm. 2017;13:727-37. 10.1016/j.sapharm.2016.06.007 27530306

[36] James S Toombs M Brodribb W Barriers and enablers to postpartum contraception among Aboriginal Australian women: Factors influencing contraceptive decisions. Aust J Prim Health. 2018;24:241-7. 10.1071/PY17041 29731003

[37] Kay M Wijayanayaka S Cook H Hollingworth S Understanding quality use of medicines in refugee communities in Australian primary care: a qualitative study. Br J Gen Pract. 2016;66:e397-409. 10.3399/bjgp16X685249 27162206 PMC4871305

[38] Kelehera H Parker R Health promotion by primary care nurses in Australian general practice. Collegian. 2013;20:215-21. 10.1016/j.colegn.2012.09.001 24596990

[39] Lawn S Lloyd A King A Sweet L Gum L Integration of primary health services: being put together does not mean they will work together. BMC Res Notes. 2014;7:66. 10.1186/1756-0500-7-66 24479605 PMC3915222

[40] Lloyd JE Delaney-Thiele D Abbott P Baldry E McEntyre E Reath J The role of primary health care services to better meet the needs of Aboriginal Australians transitioning from prison to the community. BMC Fam Pract. 2015;16:86. 10.1186/s12875-015-0303-0 26198338 PMC4508903

[41] Massi L Hickey S Maidment SJ Roe Y Kildea S Nelson C Improving interagency service integration of the Australian Nurse Family Partnership Program for First Nations women and babies: a qualitative study. Int J Equity Health. 2021;20:212. 10.1186/s12939-021-01519-x 34563171 PMC8465693

[42] McCullough K Bayes S Whitehead L Williams A Cope V We say we are doing primary health care but we’re not: Remote area nurses’ perspectives on the challenges of providing primary health care services. Collegian. 2021;28:534-40. 10.1016/j.colegn.2021.02.006

[43] Miller EM Porter JE Peel R Palliative and End-of-Life Care in the Home in Regional/Rural Victoria, Australia: The Role and Lived Experience of Primary Carers. SAGE Open Nurs. 2021;7:23779608211036284. 10.1177/23779608211036284 34869854 PMC8642066

[44] Parker R Forrest L Ward N McCracken J Cox D Derrett J How acceptable are primary health care nurse practitioners to Australian consumers? Collegian. 2013;20:35-41. 10.1016/j.colegn.2012.03.001 23678782

[45] Raymundo G Smith-Merry J McNab J Experiences of health service literacy and access amongst Australian young adults from migrant backgrounds. Health promotion journal of Australia: official journal of Australian Association of Health Promotion Professionals. 2021;32 Suppl 1:69-79. 10.1002/hpja.408 32808333

[46] Reifels L Nicholas A Fletcher J Bassilios B King K Ewen S Enhanced primary mental healthcare for Indigenous Australians: service implementation strategies and perspectives of providers. Glob Health Res Policy. 2018;3. 10.1186/s41256-018-0071-1 29881782 PMC5985563

[47] Schmidt B Campbell S McDermott R Community health workers as chronic care coordinators: evaluation of an Australian Indigenous primary health care program. Aust N Z J Public Health. 2016;40 Suppl 1:S107-14. 10.1111/1753-6405.12480 26559016

[48] Taylor KP Bessarab D Hunter L Thompson SC Aboriginal-mainstream partnerships: exploring the challenges and enhancers of a collaborative service arrangement for Aboriginal clients with substance use issues. BMC Health Serv Res. 2013;13:12. 10.1186/1472-6963-13-12 23305201 PMC3547814

[49] Tooher R Collins J Braunack-Mayer A Burgess T Skinner SR O’Keefe M Intersectoral collaboration to implement school-based health programmes: Australian perspectives. Health Promot Int. 2017;32:312-21. 26822033 10.1093/heapro/dav120

[50] Wyndow P Clifton E Walker R Improving Aboriginal Maternal Health by Strengthening Connection to Culture, Family and Community. Int J Environ Res Public Health. 2020;17:9461. 10.3390/ijerph17249461 33348723 PMC7766573

[51] Ziersch A Miller E Baak M Mwanri L Integration and social determinants of health and wellbeing for people from refugee backgrounds resettled in a rural town in South Australia: a qualitative study. BMC Public Health. 2020;20:1700. 10.1186/s12889-020-09724-z 33187489 PMC7663864

[52] Bailie C Matthews V Bailie J Burgess P Copley K Kennedy C Determinants and Gaps in Preventive Care Delivery for Indigenous Australians: A Cross-Sectional Analysis. Front Public Health. 2016;4:34. 10.3389/fpubh.2016.00034 27014672 PMC4785185

[53] Bartlem K Bowman J Freund M Wye P Lecathelinais C McElwaine K Acceptability and Receipt of Preventive Care for Chronic-Disease Health Risk Behaviors Reported by Clients of Community Mental Health Services. Psychiatr Serv. 2015;66:857-64. 10.1176/appi.ps.201400360 25930044

[54] Brodribb WE Mitchell BL van Driel ML Practice related factors that may impact on postpartum care for mothers and infants in Australian general practice: a cross-ectional survey. BMC Health Serv Res. 2016;16:244. 10.1186/s12913-016-1508-1 27400740 PMC4940844

[55] Dadich A Hosseinzadeh H Healthcare reform: implications for knowledge translation in primary care. BMC Health Serv Res. 2013;13:490. 10.1186/1472-6963-13-490 24274773 PMC3893505

[56] Dadich A Hosseinzadeh H Communication channels to promote evidence-based practice: a survey of primary care clinicians to determine perceived effects. Health Res Policy Syst. 2016;14:62. 10.1186/s12961-016-0134-z 27514872 PMC4982010

[57] Edmond KM McAuley K McAullay D Matthews V Strobel N Marriott R Quality of social and emotional wellbeing services for families of young Indigenous children attending primary care centers; a cross sectional analysis. BMC Health Serv Res. 2018;18:100. 10.1186/s12913-018-2883-6 29426308 PMC5807859

[58] Halcomb E Ashley C Australian primary health care nurses most and least satisfying aspects of work. J Clin Nurs. 2017;26:535-45. 10.1111/jocn.13479 27461981

[59] Havas K Douglas C Bonner A Person-centred care in chronic kidney disease: a cross-sectional study of patients’ desires for self-management support. BMC Nephrol. 2017;18:17. 10.1186/s12882-016-0416-2 28086812 PMC5237219

[60] Ifediora CO Rogers GD Continuity of care in after-hours house call medical services: An exploration of follow-up patterns in an Australian context. J Eval Clin Pract. 2018;24:514-20. 10.1111/jep.12902 29498149

[61] Inder KJ Handley TE Fitzgerald M Lewin TJ Coleman C Perkins D Individual and district-level predictors of alcohol use: cross sectional findings from a rural mental health survey in Australia. BMC Public Health. 2012;12:586. 10.1186/1471-2458-12-586 22853803 PMC3491021

[62] Jayasinghe UW Harris MF Parker SM Litt J van Driel M Mazza D The impact of health literacy and life style risk factors on health-related quality of life of Australian patients. Health Qual Life Outcomes. 2016;14:68. 10.1186/s12955-016-0471-1 27142865 PMC4855442

[63] Joshi C Jayasinghe UW Parker S Del Mar C Russell G Lloyd J Does health literacy affect patients’ receipt of preventative primary care? A multilevel analysis. BMC Fam Pract. 2014;15:171. 10.1186/s12875-014-0171-z 25928342 PMC4212097

[64] Kildea S Stapleton H Murphy R Kosiak M Gibbons K The maternal and neonatal outcomes for an urban Indigenous population compared with their non-Indigenous counterparts and a trend analysis over four triennia. BMC Pregnancy Childbirth. 2013;13:167. 10.1186/1471-2393-13-167 24000821 PMC3766203

[65] McElwaine KM Freund M Campbell EM Knight J Bowman JA Doherty EL The delivery of preventive care to clients of community health services. BMC Health Serv Res. 2013;13:167. 10.1186/1472-6963-13-167 23642238 PMC3656789

[66] McFarlane K Devine S Judd J Nichols N Watt K Workforce insights on how health promotion is practised in an Aboriginal Community Controlled Health Service. Aust J Prim Health. 2017;23:243-8. 10.1071/PY16033 28162218

[67] Mu C Hall J What explains the regional variation in the use of general practitioners in Australia? BMC Health Serv Res. 2020;20:325. 10.1186/s12913-020-05137-1 32306952 PMC7168818

[68] Perkins D Fuller J Kelly BJ Lewin TJ Fitzgerald M Coleman C Factors associated with reported service use for mental health problems by residents of rural and remote communities: cross-sectional findings from a baseline survey. BMC Health Serv Res. 2013;13:157. 10.1186/1472-6963-13-157 23631501 PMC3655863

[69] Reeve C Humphreys J Wakerman J Carter M Carroll V Reeve D Strengthening primary health care: achieving health gains in a remote region of Australia. Med J Aust. 2015;202:483-7. 10.5694/mja14.00894 25971572

[70] Yang B Messom R Association between potential primary care emergency service and general practitioner care utilisation in New South Wales. Emergency medicine Australasia. Emergency medicine Australasia: EMA. 2021;33:52-7. 10.1111/1742-6723.13564 32596973

[71] Yelland J Weetra D Stuart-Butler D Deverix J Leane C Ah Kit J Primary health care for Aboriginal women and children in the year after birth: findings from a population-based study in South Australia. Aust N Z J Public Health. 2016;40:418-23. 10.1111/1753-6405.12581 27624177

[72] Hegarty K Parker R Newton D Forrest L Seymour J Sanci L Feasibility and acceptability of nurse-led youth clinics in Australian general practice. Aust J Prim Health. 2013;19:159-65. 10.1071/PY12025 22951244

[73] McMurray A Ward L Johnston K Yang L Connor M The primary health care nurse of the future: Preliminary evaluation of the Nurse Navigator role in integrated care. Collegian. 2018;25:517-24. 10.1016/j.colegn.2017.12.003

[74] Munns A Community midwifery: A primary health care approach to care during pregnancy for Aboriginal and Torres Strait Islander women. Aust J Prim Health. 2021;27:57-61. 10.1071/PY20105 33502971

[75] Munns A Mahony A Miller K Whitehead A The WA Goldfields Aboriginal Community Antenatal Program-A community midwifery initiative. Coll Rev. 2016;23:367-72. 10.1016/j.colegn.2016.07.001 29116710

[76] Reeve C Humphreys J Wakerman J Carroll V Carter M O’Brien T Community participation in health service reform: the development of an innovative remote Aboriginal primary health-care service. Aust J Prim Health. 2015;21:409-16. 10.1071/PY14073 25629591

[77] Ziersch A Freeman T Javanparast S Mackean T Baum F Regional primary health care organisations and migrant and refugee health: the importance of prioritisation, funding, collaboration and engagement. Aust N Z J Public Health. 2020;44:152-9. 10.1111/1753-6405.12965 32050306

[78] Bentley M Freeman T Baum F Javanparast S Interprofessional teamwork in comprehensive primary healthcare services: Findings from a mixed methods study. J Interprof Care. 2018;32:274-83. 10.1080/13561820.2017.1401986 29182411

[79] James S Halcomb E Desborough J McInnes S Barriers and facilitators to lifestyle risk communication by Australian general practice nurses. Aust J Prim Health. 2021;27:30-5. 10.1071/PY20139 33222756

[80] Reath J Abbott P Kurti L Morgan R Martin M Parry A Supporting aboriginal and Torres Strait islander cultural educators and cultural mentors in Australian general practice education. BMC Med Educ. 2018;18:236. 10.1186/s12909-018-1340-x 30309368 PMC6182837

[81] Loban E Scott C Lewis V Law S Haggerty J Activating Partnership Assets to Produce Synergy in Primary Health Care: A Mixed Methods Study. Healthcare (Basel). 2021;9:1060. 10.3390/healthcare9081060 34442197 PMC8394800

[82] Bailie J Matthews V Laycock A Schultz R Burgess CP Peiris D Improving preventive health care in Aboriginal and Torres Strait Islander primary care settings. Global Health. 2017;13:48. 10.1186/s12992-017-0267-z 28705223 PMC5512740

[83] Carman R Andrew L Devine A Oosthuizen J Barriers to vaccination service delivery within general practice: opportunity to make a sustainable difference in Aboriginal child health? Aust N Z J Public Health. 2019;43:563-9. 10.1111/1753-6405.12937 31535420

[84] Hegney DG Patterson E Eley DS Mahomed R Young J The feasibility, acceptability and sustainability of nurse-led chronic disease management in Australian general practice: the perspectives of key stakeholders. Int J Nurs Pract. 2013;19:54-9. 10.1111/ijn.12027 23432889

[85] Hoang H Le Q Terry D Women’s access needs in maternity care in rural Tasmania, Australia: A mixed methods study. Women Birth. 2014;27:9-14. 10.1016/j.wombi.2013.02.001 23473790

[86] Lam L Ansari AS Baquir PJ Chowdhury N Tran K Bailey J Current practices, barriers and enablers for advance care planning among healthcare workers of aged care facilities in western New South Wales, Australia. Rural Remote Health. 2018;18:4714. 10.22605/RRH4714 30447659

[87] Robinson S Varhol R Ramamurthy V Denehy M Hendrie D O’Leary P The Australian primary healthcare experiment: a national survey of Medicare Locals. BMJ Open. 2015;5:e007191. 10.1136/bmjopen-2014-007191 25818276 PMC4386220

[88] Swami M Scott A Impact of rural workforce incentives on access to GP services in underserved areas: Evidence from a natural experiment. Social science & medicine (1982). 2021;281:114045. 10.1016/j.socscimed.2021.114045 34091229

[89] Zander KK Taylor AJ Carson DB Impacts of Service and Infrastructure Provision on Indigenous Temporary Mobility in the Northern Territory of Australia: Insights from the 2011 Census. Popul Space Place. 2016;22:99-116. 10.1002/psp.1871

[90] Bartlem K Wolfenden L Colyvas K Campbell L Freund M Doherty E The association between the receipt of primary care clinician provision of preventive care and short term health behaviour change. Prev Med. 2019;123:308-15. 10.1016/j.ypmed.2019.03.046 30930261

[91] Bloomfield JG Gordon CJ Williams AM Aggar C Nursing students’ intentions to enter primary health care as a career option: Findings from a national survey. Collegian. 2015;22:161-7. 10.1016/j.colegn.2015.02.001 26281403

[92] Keane S Lincoln M Rolfe M Smith T Retention of the rural allied health workforce in New South Wales: a comparison of public and private practitioners. BMC Health Serv Res. 2013;13:32. 10.1186/1472-6963-13-32 23351491 PMC3599445

[93] Mazumdar S Feng X Konings P McRae I Girosi F A brief report on Primary Care Service Area catchment geographies in New South Wales Australia. Int J Health Geogr. 2014;13:38. 10.1186/1476-072X-13-38 25292210 PMC4197238

[94] Morell AL Kiem S Millsteed MA Pollice A Attraction, recruitment and distribution of health professionals in rural and remote Australia: early results of the Rural Health Professionals Program. Hum Resour Health. 2014;12:15. 10.1186/1478-4491-12-15 24602181 PMC3973976

[95] Quach J Oberklaid F Gold L Lucas N Mensah FK Wake M Primary health-care costs associated with special health care needs up to age 7 years: Australian population-based study. J Paediatr Child Health. 2014;50:768-74. 10.1111/jpc.12649 24923806

[96] Zurynski Y Ansell J Ellis LA Pomare C Smith CL Holt J Accessible and affordable healthcare? Views of Australians with and without chronic conditions. Intern Med J. 2021;51:1060-7. 10.1111/imj.15172 33350562 PMC8361684

[97] Khanam MA Kitsos A Stankovich J Kinsman L Wimmer B Castelino R Chronic kidney disease monitoring in Australian general practice. Aust J Gen Pract. 2019;48:132-7. 10.31128/AJGP-07-18-4630 31256479

[98] Doyle J Atkinson-Briggs S Atkinson P Firebrace B Calleja J Reilly R A prospective evaluation of first people’s health promotion program design in the goulburn-murray rivers region. BMC Health Serv Res. 2016;16:645. 10.1186/s12913-016-1878-4 27832789 PMC5105254

[99] Inacio MC Amare AT Whitehead C Bray SCE Corlis M Visvanathan R Factors associated with accessing aged care services in Australia after approval for services: Findings from the historical cohort of the Registry of Senior Australians. Australas J Ageing. 2020;39:e382-92. 10.1111/ajag.12760 31975527 PMC7687099

[100] Tennett D Kearney L Kynn M Access and outcomes of general practitioner obstetrician (rural generalist)-supported birthing units in Queensland. Aust J Rural Health. 2020;28:42-50. 10.1111/ajr.12593 31903661 PMC7328769

[101] Thomas SL Zhao Y Guthridge SL Wakerman J The cost-effectiveness of primary care for Indigenous Australians with diabetes living in remote Northern Territory communities. Med J Aust. 2014;200:658-62. 10.5694/mja13.11316 24938348

[102] Wood A MacKay D Fitzsimmons D Derkenne R Kirkham R Boyle JA Primary Health Care for Aboriginal Australian Women in Remote Communities after a Pregnancy with Hyperglycaemia. Int J Environ Res Public Health. 2020;17:720. 10.3390/ijerph17030720 31979123 PMC7037226

[103] Valery PC Bernardes CM de Witt A Martin J Walpole E Garvey G Patterns of primary health care service use of Indigenous Australians diagnosed with cancer. Supportive care in cancer: official journal of the Multinational Association of Supportive Care in Cancer. 2020;28:317-27. 10.1007/s00520-019-04821-1 31049670

[104] Hussain J Robinson A Stebbing M McGrail M More is more in remote Central Australia: more provision of primary healthcare services is associated with more acute medical evacuations and more remote telephone consultations. Rural Remote Health. 2014;14:2796. 10.22605/RRH2796 25391688

[105] Jones MP Zhao Y Guthridge S Russell DJ Ramjan M Humphreys JS Effects of turnover and stability of health staff on quality of care in remote communities of the Northern Territory, Australia: a retrospective cohort study. BMJ Open. 2021;11:e055635. 10.1136/bmjopen-2021-055635 34667018 PMC8527144

[106] Sinclair C Gates K Evans S Auret KA Factors Influencing Australian General Practitioners’ Clinical Decisions Regarding Advance Care Planning: A Factorial Survey. J Pain Symptom Manage. 2016;51:718-27.e2. 10.1016/j.jpainsymman.2015.11.014 26706628

[107] Khalil H Lee S The implementation of a successful medication safety program in a primary care. J Eval Clin Pract. 2018;24:403-7. 10.1111/jep.12870 29322597

[108] John JR Tannous WK Jones A Effectiveness of a patient-centered medical home model of primary care versus standard care on blood pressure outcomes among hypertensive patients. Hypertens Res. 2020;43:892-902. 10.1038/s41440-020-0431-3 32238947

[109] John JR Tannous WK Jones A Outcomes of a 12-month patient-centred medical home model in improving patient activation and self-management behaviours among primary care patients presenting with chronic diseases in Sydney, Australia: a before-and-after study. BMC Fam Pract. 2020;21:158. 10.1186/s12875-020-01230-w 32770944 PMC7414685

[110] John JR Tannous WK Jones A Effectiveness of a Patient-Centre Medical Home model on diabetes and other clinically relevant outcomes among primary care patients diagnosed with type-2 diabetes in Sydney, Australia. Prim Care Diabetes. 2021;15:464-71. 10.1016/j.pcd.2021.01.007 33547009

[111] John JR Tannous WK Jones A Changes in health-related quality of life before and after a 12-month enhanced primary care model among chronically ill primary care patients in Australia. Health Qual Life Outcomes. 2020;18:288. 10.1186/s12955-020-01539-1 32831086 PMC7445903

[112] Percival N O’Donoghue L Lin V Tsey K Bailie RS Improving Health Promotion Using Quality Improvement Techniques in Australian Indigenous Primary Health Care. Front Public Health. 2016;4:53. 10.3389/fpubh.2016.00053 27066470 PMC4812048

[113] Sawyer MG Frost L Bowering K Lynch J Effectiveness of nurse home-visiting for disadvantaged families: results of a natural experiment. BMJ Open. 2013;3:e002720. 10.1136/bmjopen-2013-002720 23619089 PMC3641507

[114] Harris MF Parker SM Litt J van Driel M Russell G Mazza D An Australian general practice based strategy to improve chronic disease prevention, and its impact on patient reported outcomes: evaluation of the preventive evidence into practice cluster randomised controlled trial. BMC Health Serv Res. 2017;17:637. 10.1186/s12913-017-2586-4 28886739 PMC5591527

[115] Forster DA McLachlan HL Davey MA Biro MA Farrell T Gold L Continuity of care by a primary midwife (caseload midwifery) increases women’s satisfaction with antenatal, intrapartum and postpartum care: results from the COSMOS randomised controlled trial. BMC Pregnancy Childbirth. 2016;16:28. 10.1186/s12884-016-0798-y 26841782 PMC4739100

[116] Liaw ST Wade V Furler JS Hasan I Lau P Kelaher M Cultural respect in general practice: a cluster randomised controlled trial. Med J Aust. 2019;210:263-8. 10.5694/mja2.50031 30802313

[117] Bartlem KM Bowman J Freund M Wye PM Barker D McElwaine KM Effectiveness of an intervention in increasing the provision of preventive care by community mental health services: a non-randomized, multiple baseline implementation trial. Implement Sci. 2016;11:46. 10.1186/s13012-016-0408-4 27039077 PMC4818909

[118] Mitchell GK Young CE Janamian T Beaver KM Johnson JLK Hannan-Jones C Mutch AJ Factors affecting the embedding of integrated primary-secondary care into a health district. Aust J Prim Health. 2020;26:216-21. 10.1071/PY18177 32527371

[119] Hulme Chambers A Tomnay J Stephens K Crouch A Whiteside M Love P Facilitators of community participation in an Aboriginal sexual health promotion initiative. Rural Remote Health. 2018;18:4245. 10.22605/RRH4245 29655365

[120] Odgers-Jewell K Isenring E Thomas R Reidlinger DP Process evaluation of a patient-centred, patient-directed, group-based education program for the management of type 2 diabetes mellitus. Nutr Diet. 2017;74:243-52. 10.1111/1747-0080.12327 28731607

[121] Thomas S Higgins H Leask J Menning L Habersaat K Massey P Improving child immunisation rates in a disadvantaged community in New South Wales, Australia: A process evaluation for research translation. Aust J Prim Health. 2019;25:310-6. 10.1071/PY19016 31479627

[122] Dasgupta P Condon JR Whop LJ Aitken JF Garvey G Wenitong M Access to Aboriginal Community-Controlled Primary Health Organizations Can Explain Some of the Higher Pap Test Participation Among Aboriginal and Torres Strait Islander Women in North Queensland, Australia. Front Oncol. 2021;11:725145. 10.3389/fonc.2021.725145 34395296 PMC8355598

[123] Brown SJ Gartland D Weetra D Leane C Francis T Mitchell A Health care experiences and birth outcomes: Results of an Aboriginal birth cohort. Women Birth. 2019;32:404-11. 10.1016/j.wombi.2019.05.015 31202584

[124] Larkins S Woods CE Matthews V Thompson SC Schierhout G Mitropoulos M Responses of Aboriginal and Torres Strait Islander Primary Health-Care Services to Continuous Quality Improvement Initiatives. Front Public Health. 2016;3:288. 10.3389/fpubh.2015.00288 26835442 PMC4720733

[125] Ryan SM Toumbourou JW Jorm AF Factors Associated With Service Use for Young Adolescents With Mental Health Problems: Findings From an Australian Longitudinal Study. SAGE Open. 2014;4. 10.1177/2158244014556286

[126] Isaacs A Beauchamp A Sutton K Kocaali N Care Coordination Can Reduce Unmet Needs of Persons With Severe and Persistent Mental Illness. Front Psychiatry. 2019;10:563. 10.3389/fpsyt.2019.00563 31447714 PMC6697021

[127] Rolfe MI Donoghue DA Longman JM Pilcher J Kildea S Kruske S The distribution of maternity services across rural and remote Australia: does it reflect population need? BMC Health Serv Res. 2017;17. 10.1186/s12913-017-2084-8 28231830 PMC5324256

[128] Slimings C Moore M Geographic variation in health system performance in rural areas of New South Wales, Australia. Aust J Rural Health. 2021;29:41-51. 10.1111/ajr.12688 33567162

[129] Wiggers J McElwaine K Freund M Campbell L Bowman J Wye P Increasing the provision of preventive care by community healthcare services: a stepped wedge implementation trial. Implement Sci. 2017;12:105. 10.1186/s13012-017-0636-2 28830568 PMC5567434

[130] Baum F Freeman T Jolley G Lawless A Bentley M Värttö K Health promotion in Australian multi-disciplinary primary health care services: case studies from South Australia and the Northern Territory. Health Promot Int. 2014;29:705-19. 10.1093/heapro/dat029 23656732

[131] Callander E Larkins S Corscadden L Variations in out-of-pocket costs for primary care services across Australia: a regional analysis. Aust J Prim Health. 2017;23:379-85. 10.1071/PY16127 28502310

[132] Reifels L Bassilios B Nicholas A Fletcher J King K Ewen S Improving access to primary mental healthcare for Indigenous Australians. Aust N Z J Psychiatry. 2015;49:118-28. 10.1177/0004867414562046 25492971

[133] Spaeth BA Kaambwa B Shephard MDS Omond R Economic evaluation of point-of-care testing in the remote primary health care setting of Australia’s Northern Territory. Clinicoecon Outcomes Res. 2018;10:269-77. 10.2147/CEOR.S160291 29881299 PMC5985789

[134] Haggerty JL Beaulieu M-D Pineault R Burge F Lévesque J-F Santor DA Comprehensiveness of care from the patient perspective: comparison of primary healthcare evaluation instruments. Healthc Policy. 2011;7:154-66. 10.12927/hcpol.2011.22708 23205042 PMC3399439

[135] Sturmberg JP. A Complex Adaptive Health System Redesign Based on “First Principles”. Health System Redesign: Springer; 2018. p. 75-96.

[136] Senderowicz L Pearson E Hackett K Huber-Krum S Francis JM Ulenga N ‘I haven’t heard much about other methods’: quality of care and person-centredness in a programme to promote the postpartum intrauterine device in Tanzania. BMJ Glob Health. 2021;6:e005775. 10.1136/bmjgh-2021-005775 34162627 PMC8230964

[137] Goonetilleke M. Global Health Systems: Design and Reform. Handbook of Global Health: Springer; 2021. p. 1541-68.

[138] Bath J Wakerman J Impact of community participation in primary health care: what is the evidence? Aust J Prim Health. 2015;21:2-8. 10.1071/PY12164 24176202

[139] Nicholson C, Jackson C, Marley J. A framework for integrated primary/secondary health care governance in Australia: results of a systematic review. APHCRI Centre of Research Excellence in Primary Health Care Microsystems, The University of Queensland. 2014.

[140] Thomas SL Wakerman J Humphreys JS Ensuring equity of access to primary health care in rural and remote Australia - what core services should be locally available? Int J Equity Health. 2015;14:111. 10.1186/s12939-015-0228-1 26510998 PMC4625941

[141] (SIG) PHAASIG. Primary Health Care (PHC) Policy Position Statement. 2020.

[142] Health AGDo. Future focused primary health care: Australia’s Primary Health Care 10 Year Plan 2022-2032. 2022.

[143] Muldoon LK Hogg WE Levitt M Primary care (PC) and primary health care (PHC). Can J Public Health. 2006;97:409-11. 10.1007/BF03405354 17120883 PMC6976192

