# Supplementary material for: Successes and challenges of primary health care in Australia: A scoping review and comparative analysis
Source: J Glob Health. 2023 Jul 30;13:04043. doi: 10.7189/jogh.13.04043 (PMC10311945; doi:10.7189/jogh.13.04043)
Supplement: Online Supplementary Document [file jogh-13-04043-s001.pdf]

Supplementary files and tables

Supplementary file 1. Search strategies for the databases

PubMed Search Strategy

Search Details	Results
(((("comprehensive health care"[mh:noexps] OR "comprehensive health care"[tw] OR "preventative care" [tw] OR "curative care" [tw] OR "palliative care" [tw] OR "rehabilit* care" [tw] OR "health promotion" [tw] OR "comprehensive health service" [tw] OR "inclusive health care" [tw] OR "inclusive health service" [tw] OR "compressive health*" [tw] OR "inclusive health*" [tw] OR "inclusive health servic*" [tw] OR "inclusivity of health care" [tw] OR "comprehensive health care" [tiab] OR "preventative care" [tiab] OR "curative care" [tiab] OR "palliative care" [tiab] OR "rehabilitative care" [tiab] OR "health promotion" [tiab] OR "comprehensive health service" [tiab] OR "inclusive health care" [tiab] OR "inclusive health service" [tiab] OR "compressive health*" [tiab] OR "inclusive health*" [tiab] OR "inclusive health servic*" [tiab] OR "inclusivity of health care" [tiab]) OR ("health service* accessibility" [mh:noexp] OR "health care access" [mh:noexp] OR "health care accessibility" [mh:noexp] OR "access to health servic*" [mh:noexp] OR "access to health care" [mh:noexp] OR "healthcare disparit*" [mh:noexp] OR "medically underserved area" [mh:noexp] OR "medical indigency" [tw] OR "health service* accessibility" [tiab] OR "health care access" [tiab] OR "health care accessibility" [tiab] OR "access to health servic*" [tiab] OR "access to health care" [tiab] OR "healthcare disparit*" [tiab] OR "medically underserved area" [tiab] OR "medical indigency" [tiab]) OR ("health care coverage" [mh:noexp] OR "health service* coverage" [mh:noexp] OR "healthcare coverage" [mh:noexp] OR "coverage of health*" [mh:noexp] OR "coverage of health service*" [mh:noexp] OR "scope of health care" [mh:noexp] OR "health care" [tw] OR "health service* coverage" [tw] OR "healthcare coverage" [tw] OR "coverage of health*" [tw] OR "coverage of health service*" [tw] OR "scope of health care" [tw] OR "health care" [tiab] OR "health service* coverage" [tiab] OR "healthcare coverage" [tiab] OR" coverage of health*" [tiab] OR "coverage of health service*" [tiab] OR "scope of health care" [tiab]) OR ("continuity of patient care" [mh:noexp] OR "continuity of care" [mh:noexp] OR "continuum of care" [mh:noexp] OR "healthcare continuum" [mh:noexp] OR "continuity of patient care" [tw] OR "continuity of care" [tw] OR "continuum of care" [tw] OR "healthcare continuum" [tw] OR "continuity of patient care" [tiab] OR "continuity of care" [tiab] OR "continuum of care" [tiab] OR "healthcare continuum" [tiab]) OR ("quality of health care" [mh:noexp] OR "quality of care" [mh:noexp] OR "quality of health servic*" [mh:noexp] OR "input quality" [mh:noexp] OR "process qualit"y [mh:noexp] OR "quality of health care" [tw] OR "quality of care" [tw] OR "quality of health servic*" [tw] OR "safe healthcare" [mh:noexp] OR "effective healthcare" [mh:noexp] OR "efficient health care" [mh:noexp] OR "quality of health care" [tiab] OR "quality of care" [tiab] OR "quality of health servic*" [tiab] OR "input quality" [tiab] OR "process quality" [tiab] OR "quality of health care" [tiab] OR "quality of care" [tiab] OR "quality of health servic*" [tiab] OR "safe healthcare" [tiab] OR "effective healthcare" [tiab] OR "efficient health care" [tiab]) OR ("person centeredness" [mh:noexp] OR "client centeredness" [mh:noexp] OR "individualised care" [mh:noexp] OR "responsive care" [mh:noexp] OR "client satisfaction" [mh:noexp] OR "client satisfaction" [mh:noexp] OR "person "centeredness[tw] OR "client centeredness" [tw] OR "individualised care" [tw] OR "responsive care" [tw] OR "client satisfaction" [tw] OR "client satisfaction" [tw] OR "person-centeredness" [tiab] OR "client centeredness" [tiab] OR "individualised care" [tiab] OR "responsive care" [tiab] OR "client satisfaction" [tiab] OR "client satisfaction" [tiab]) OR ("coordinated health care" [mh:noexp] OR "coordinated health service*" [mh:noexp] OR "integrated health servic*" [mh:noexp] OR "coordinat*" [tw] OR "integrat*"[tw] OR "multi-sectoral" [tw] OR "multi sectoral"[tw] OR "intra-sectoral"[tw] OR "inra sectoral" [tw] OR "trans-sectoral"[tw] OR "trans sectoral"[tw] OR "public-private partnership"[tw] OR "public private partnership"[tw] OR "community involvement" [tw] OR "community participation" [tw] OR "centrelink" [tw] OR "coordinat*" [tiab] OR "integrat*"[tiab] OR "multi-sectoral"[tiab] OR "multi sectoral"[tiab] OR "intra-sectoral"[tiab] OR "inra sectoral"[tiab] OR "trans-sectoral"[tiab] OR "trans sectoral"[tiab] OR "public-private partnership"[tiab] OR "public private partnership"[tiab] OR "community involvement"[tiab] OR "community participation" [tiab] OR "centrelink" [tiab]) OR (("accountability"[tw] OR "reporting"[tw] OR "transparency"[tw] OR "reward"[tw] OR "punishment"[tw] OR "monitoring"[tw] OR "evaluation"[tw] OR "monitoring and evaluation"[tw]) OR ("delivery of health care"[tw] OR "health care delivery" [tw] OR " health system"[tw] OR "health institu*"[tw) AND ("accountability"[tiab] OR "reporting"[tiab] OR "transparency"[tiab] OR "reward"[tiab] OR "punishment"[tiab] OR "monitoring"[tiab] OR "evaluation"[tiab] OR "monitoring and evaluation"[tiab]))) AND ((journalarticle[Filter]) AND (fft[Filter]) AND (humans[Filter]) AND (2013/1/1:2021/12/30[pdat]) AND (english[Filter]))) AND (((Primary care[tw] OR General practi*[tw] OR Primary health care[tw] OR Community mental health services[mh:noexp] OR Family practice[mh:noexp] OR Home care services[mh:noexp] OR Family physicians[mh:noexp] OR Community health services[mh:noexp] OR Community health nursing[mh:noexp] OR Community pharmacy services[mh:noexp] OR Community health workers[mh:noexp] OR Preventive health services[mh:noexp]) AND Medline[sb]) OR ((Primary care[tiab] OR General practi*[tiab] OR Primary health*[tiab] OR Community mental health*[tiab] OR Family practice[tiab] OR Family medicine[tiab] OR Family physician*[tiab] OR Home care[tiab] OR Home based[tiab] OR Home health*[tiab] OR Community health*[tiab] OR Community nurs*[tiab] OR health visit*[tiab] OR Community pharmac*[tiab] OR Preventive care[tiab] OR Prevention program*[tiab] OR Preventive service*[tiab] OR Preventive health[tiab] OR Health promotion[tiab]) NOT Medline[sb]) AND (.au[ad] OR Australia*[tw] OR Australia*[ad] OR New South Wales[tw] OR New South Wales[ad] OR Victoria[tw] OR Victoria[ad] OR Queensland[tw] OR Queensland[ad] OR South Australia[tw] OR South Australia[ad] OR Western Australia[tw] OR Western Australia[ad] OR Tasmania[tw] OR Tasmania[ad] OR Australian Capital Territory[tw] OR Northern Territory[tw] OR Northern Territory[ad]))) AND ((journalarticle[Filter]) AND (fft[Filter]) AND (humans[Filter]) AND (2013/1/1:2021/12/30[pdat]) AND (english[Filter]))	5,887

Embase Search strategy

No.	Query	Results
#1	'comprehensive health care'/de OR 'comprehensive health care':ti,ab,kw OR 'preventative care':ti,ab,kw OR 'curative care':ti,ab,kw OR 'rehabilit* care':ti,ab,kw OR 'comprehensive health care':ti,ab OR 'preventative care':ti,ab OR 'curative care':ti,ab OR 'palliative care':ti,ab,kw OR 'rehabilitative care':ti,ab,kw OR 'health promotion':ti,ab,kw OR 'comprehensive health service':ti,ab,kw OR 'inclusive health care':ti,ab,kw OR 'inclusive health service':ti,ab,kw OR 'compressive health*':ti,ab,kw OR 'inclusive health*':ti,ab,kw OR 'inclusive health servic*':ti,ab,kw OR 'inclusivity of health care':ti,ab,kw AND ([embase]/lim AND [english]/lim AND (2013:py OR 2014:py OR 2015:py OR 2016:py OR 2017:py OR 2018:py OR 2019:py OR 2020:py OR 2021:py) AND 'article'/it AND ('.au' OR 'australia*':ti,ab,kw OR 'australia*' OR 'new south wales':ti,ab,kw OR 'new south wales' OR 'victoria':ti,ab,kw OR 'victoria' OR 'queensland':ti,ab,kw OR 'queensland' OR 'south australia':ti,ab,kw OR 'south australia' OR 'western australia':ti,ab,kw OR 'western australia' OR 'tasmania':ti,ab,kw OR 'tasmania' OR 'australian capital territory':ti,ab,kw OR 'northern territory':ti,ab,kw OR 'northern territory'))	3,144

#2	'health service* accessibility' OR 'health care access'/de OR 'health care accessibility' OR 'access to health servic*' OR 'access to health care'/de OR 'healthcare disparit*' OR 'medically underserved area'/de OR 'health service* accessibility':ti,ab,kw OR 'health care access':ti,ab,kw OR 'health care accessibility':ti,ab,kw OR 'access to health servic*':ti,ab,kw OR 'access to health care':ti,ab,kw OR 'healthcare disparit*':ti,ab,kw OR 'medically underserved area':ti,ab,kw OR 'medical indigency':ti,ab,kw AND ([embase]/lim AND [english]/lim AND (2013:py OR 2014:py OR 2015:py OR 2016:py OR 2017:py OR 2018:py OR 2019:py OR 2020:py OR 2021:py) AND 'article'/it AND ('.au' OR 'australia*':ti,ab,kw OR 'australia*' OR 'new south wales':ti,ab,kw OR 'new south wales' OR 'victoria':ti,ab,kw OR 'victoria' OR 'queensland':ti,ab,kw OR 'queensland' OR 'south australia':ti,ab,kw OR 'south australia' OR 'western australia':ti,ab,kw OR 'western australia' OR 'tasmania':ti,ab,kw OR 'tasmania' OR 'australian capital territory':ti,ab,kw OR 'northern territory':ti,ab,kw OR 'northern territory'))	3,855
#3	'health care coverage' OR 'health service coverage' OR 'scope of health care' OR 'health service coverage':ti,ab,kw OR 'health care coverage':ti,ab,kw OR 'coverage of health care':ti,ab,kw OR 'coverage of health service':ti,ab,kw OR 'scope of health care':ti,ab,kw AND ([embase]/lim AND [english]/lim AND (2013:py OR 2014:py OR 2015:py OR 2016:py OR 2017:py OR 2018:py OR 2019:py OR 2020:py OR 2021:py) AND 'article'/it AND ('.au' OR 'australia*':ti,ab,kw OR 'australia*' OR 'new south wales':ti,ab,kw OR 'new south wales' OR 'victoria':ti,ab,kw OR 'victoria' OR 'queensland':ti,ab,kw OR 'queensland' OR 'south australia':ti,ab,kw OR 'south australia' OR 'western australia':ti,ab,kw OR 'western australia' OR 'tasmania':ti,ab,kw OR 'tasmania' OR 'australian capital territory':ti,ab,kw OR 'northern territory':ti,ab,kw OR 'northern territory'))	18
#4	'continuity of patient care'/de OR 'continuity of care'/de OR 'healthcare continuum' OR 'continuity of patient care':ti,ab,kw OR 'continuity of care':ti,ab,kw OR 'continuum of care':ti,ab,kw OR 'healthcare continuum':ti,ab,kw AND [embase]/lim AND [english]/lim AND (2013:py OR 2014:py OR 2015:py OR 2016:py OR 2017:py OR 2018:py OR 2019:py OR 2020:py OR 2021:py) AND 'article'/it AND ('.au' OR 'australia*':ti,ab,kw OR 'australia*' OR 'new south wales':ti,ab,kw OR 'new south wales' OR 'victoria':ti,ab,kw OR 'victoria' OR 'queensland':ti,ab,kw OR 'queensland' OR 'south australia':ti,ab,kw OR 'south australia' OR 'western australia':ti,ab,kw OR 'western australia' OR 'tasmania':ti,ab,kw OR 'tasmania' OR 'australian capital territory':ti,ab,kw OR 'northern territory':ti,ab,kw OR 'northern territory')	4,691
#5	('quality of health care'/de OR 'quality of care'/de OR 'quality of health servic*' OR 'input quality' OR 'process quality' OR 'safe healthcare' OR 'effective healthcare' OR 'efficient health care' OR 'input quality':ti,ab,kw OR 'process quality':ti,ab,kw OR 'quality of health care':ti,ab,kw OR 'quality of care':ti,ab,kw OR 'quality of health servic*':ti,ab,kw OR 'safe healthcare':ti,ab,kw OR 'effective healthcare':ti,ab,kw OR 'efficient health care':ti,ab,kw) AND [embase]/lim AND [english]/lim AND (2013:py OR 2014:py OR 2015:py OR 2016:py OR 2017:py OR 2018:py OR 2019:py OR 2020:py OR 2021:py) AND ('.au' OR 'australia*':ti,ab,kw OR 'australia*' OR 'new south wales':ti,ab,kw OR 'new south wales' OR 'victoria':ti,ab,kw OR 'victoria' OR 'queensland':ti,ab,kw OR 'queensland' OR 'south australia':ti,ab,kw OR 'south australia' OR 'western australia':ti,ab,kw OR 'western australia' OR 'tasmania':ti,ab,kw OR 'tasmania' OR 'australian capital territory':ti,ab,kw OR 'northern territory':ti,ab,kw OR 'northern territory')	6,482
#6	'person centred' OR 'client centred' OR 'individualised care' OR 'responsive care' OR 'client satisfaction' OR 'person centeredness':ti,ab,kw OR 'person-centeredness':ti,ab,kw OR 'client centeredness':ti,ab,kw OR 'individualised care':ti,ab,kw OR 'responsive care':ti,ab,kw OR 'client satisfaction':ti,ab,kw AND ([embase]/lim AND [english]/lim AND (2013:py OR 2014:py OR 2015:py OR 2016:py OR 2017:py OR 2018:py OR 2019:py OR 2020:py OR 2021:py) AND 'article'/it AND ('.au' OR 'australia*':ti,ab,kw OR 'australia*' OR 'new south wales':ti,ab,kw OR 'new south wales' OR 'victoria':ti,ab,kw OR 'victoria' OR 'queensland':ti,ab,kw OR 'queensland' OR 'south australia':ti,ab,kw OR 'south australia' OR 'western australia':ti,ab,kw OR 'western australia' OR 'tasmania':ti,ab,kw OR 'tasmania' OR 'australian capital territory':ti,ab,kw OR 'northern territory':ti,ab,kw OR 'northern territory'))	303
#7	'coordinated health care' OR 'coordinated health service' OR 'integrated health servic*' OR 'coordinat*':ti,ab,kw OR 'integrat*':ti,ab,kw OR 'multi-sectoral':ti,ab,kw OR 'multi sectoral':ti,ab,kw OR 'intra-sectoral':ti,ab,kw OR 'inra sectoral':ti,ab,kw OR 'trans-sectoral':ti,ab,kw OR 'trans sectoral':ti,ab,kw OR 'public-private partnership':ti,ab,kw OR 'public private partnership':ti,ab,kw OR 'community involvement':ti,ab,kw OR 'community participation':ti,ab,kw OR 'centrelink':ti,ab,kw AND ([embase]/lim AND [english]/lim AND (2013:py OR 2014:py OR 2015:py OR 2016:py OR 2017:py OR 2018:py OR 2019:py OR 2020:py OR 2021:py) AND 'article'/it AND ('.au' OR 'australia*':ti,ab,kw OR 'australia*' OR 'new south wales':ti,ab,kw OR 'new south wales' OR 'victoria':ti,ab,kw OR 'victoria' OR 'queensland':ti,ab,kw OR 'queensland' OR 'south australia':ti,ab,kw OR 'south australia' OR 'western australia':ti,ab,kw OR 'western australia' OR 'tasmania':ti,ab,kw OR 'tasmania' OR 'australian capital territory':ti,ab,kw OR 'northern territory':ti,ab,kw OR 'northern territory'))	14,292
#8	('accountability':ti,ab,kw OR 'reporting in healthcare':ti,ab,kw OR 'transparency in healthcare':ti,ab,kw OR 'reward':ti,ab,kw OR 'punishment':ti,ab,kw OR 'monitoring':ti,ab,kw OR 'evaluation':ti,ab,kw OR 'monitoring and evaluation':ti,ab,kw) AND [embase]/lim AND [english]/lim AND (2013:py OR 2014:py OR 2015:py OR 2016:py OR 2017:py OR 2018:py OR 2019:py OR 2020:py OR 2021:py) AND 'article'/it AND ('.au' OR 'australia*':ti,ab,kw OR 'australia*' OR 'new south wales':ti,ab,kw OR 'new south wales' OR 'victoria':ti,ab,kw OR 'victoria' OR 'queensland':ti,ab,kw OR 'queensland' OR 'south australia':ti,ab,kw OR 'south australia' OR 'western australia':ti,ab,kw OR 'western australia' OR 'tasmania':ti,ab,kw OR 'tasmania' OR 'australian capital territory':ti,ab,kw OR 'northern territory':ti,ab,kw OR 'northern territory')	28,193
#9	('primary health care':ti,ab,kw OR 'community mental health services'/de OR 'family practice'/de OR 'home care services'/de OR 'family physicians'/de OR 'community health services'/de OR 'community health nursing'/de OR 'community pharmacy services'/de OR 'community health workers'/de OR 'preventive health services'/de OR 'primary care':ti,ab,kw OR 'general practi*':ti,ab,kw OR 'primary health*':ti,ab,kw OR 'community mental health*':ti,ab,kw OR 'family practice':ti,ab,kw OR 'family medicine':ti,ab,kw OR 'family physician*':ti,ab,kw OR 'home care':ti,ab,kw OR 'home based':ti,ab,kw OR 'home health*':ti,ab,kw OR 'community health*':ti,ab,kw OR 'community nurs*':ti,ab,kw OR 'health visit*':ti,ab,kw OR 'community pharmac*':ti,ab,kw OR 'preventive care':ti,ab,kw OR 'prevention program*':ti,ab,kw OR 'preventive service*':ti,ab,kw OR 'preventive health':ti,ab,kw OR 'health promotion':ti,ab,kw) AND [embase]/lim AND [english]/lim AND (2013:py OR 2014:py OR 2015:py OR 2016:py OR 2017:py OR 2018:py OR 2019:py OR 2020:py OR 2021:py) AND 'article'/it AND ('.au' OR 'australia*':ti,ab,kw OR 'australia*' OR 'new south wales':ti,ab,kw OR 'new south wales' OR 'victoria':ti,ab,kw OR 'victoria' OR 'queensland':ti,ab,kw OR 'queensland' OR 'south australia':ti,ab,kw OR 'south australia' OR 'western australia':ti,ab,kw OR 'western australia' OR 'tasmania':ti,ab,kw OR 'tasmania' OR 'australian capital territory':ti,ab,kw OR 'northern territory':ti,ab,kw OR 'northern territory')	10,774
#10	#1 OR #2 OR #3 OR #4 OR #5 OR #6 OR #7 OR #8	58,183
#11	#9 AND #10	4,669

Scopus search strategy

No.	Query	Results
#1	(TITLE-ABS ("comprehensive health care") OR TITLE-ABS ("preventative care") OR TITLE-ABS ("preventative care") OR TITLE-ABS ("curative care") OR TITLE-ABS ("palliative care") OR TITLE-ABS ("rehabilitat* care") OR TITLE-ABS ("health promotion") OR TITLE-ABS ("comprehensive health service") OR TITLE-ABS ("inclusive health care") OR TITLE-ABS ("inclusive health service") OR TITLE-ABS ("inclusive health service") OR TITLE-ABS ("inclusivity of health care")) AND (LIMIT-TO (PUBSTAGE , "final")) AND (LIMIT-TO (PUBYEAR , 2021) OR LIMIT-TO (PUBYEAR , 2020) OR LIMIT-TO (PUBYEAR , 2019) OR LIMIT-TO (PUBYEAR , 2018) OR LIMIT-TO (PUBYEAR , 2017) OR LIMIT-TO (PUBYEAR , 2016) OR LIMIT-TO (PUBYEAR , 2015) OR LIMIT-TO (PUBYEAR , 2014) OR LIMIT-TO (PUBYEAR , 2013)) AND (LIMIT-TO (DOCTYPE , "ar")) AND (LIMIT-TO (SUBJAREA , "MEDI") OR LIMIT-TO (SUBJAREA , "NURS")) AND (LIMIT-TO (LANGUAGE , "English")) AND (LIMIT-TO (SRCTYPE , "j")) AND (LIMIT-TO (SRCTYPE , "j")) AND (AFFIL (".au") OR AFFIL ("australia*") OR AFFIL ("new south wales") OR AFFIL ("victoria") OR AFFIL ("queensland") OR AFFIL ("south australia") OR AFFIL ("western australia") OR AFFIL ("tasmania") OR AFFIL ("australian capital territory") OR AFFIL ("northern territory"))	87,777
#2	(TITLE-ABS ("health service accessibility") OR TITLE-ABS ("health care access") TITLE-ABS ("health care accessibility") OR TITLE-ABS ("access to health care") OR TITLE-ABS ("access to health service") OR TITLE-ABS ("healthcare disparity") OR TITLE-ABS ("medically underserved area") OR TITLE-ABS ("medical indigency")) AND (LIMIT-TO (PUBSTAGE , "final")) AND (LIMIT-TO (PUBYEAR , 2021) OR LIMIT-TO (PUBYEAR , 2020) OR LIMIT-TO (PUBYEAR , 2019) OR LIMIT-TO (PUBYEAR , 2018) OR LIMIT-TO (PUBYEAR , 2017) OR LIMIT-TO (PUBYEAR , 2016) OR LIMIT-TO (PUBYEAR , 2015) OR LIMIT-TO (PUBYEAR , 2014) OR LIMIT-TO (PUBYEAR , 2013)) AND (LIMIT-TO (DOCTYPE , "ar")) AND (LIMIT-TO (SUBJAREA , "MEDI") OR LIMIT-TO (SUBJAREA , "NURS")) AND (LIMIT-TO (LANGUAGE , "English")) AND (LIMIT-TO (SRCTYPE , "j")) AND (LIMIT-TO (SRCTYPE , "j")) AND (AFFIL (".au") OR AFFIL ("australia*") OR AFFIL ("new south wales") OR AFFIL ("victoria") OR AFFIL ("queensland") OR AFFIL ("south australia") OR AFFIL ("western australia") OR AFFIL ("tasmania") OR AFFIL ("australian capital territory") OR AFFIL ("northern territory"))	517
#3	(TITLE-ABS ("health care coverage") OR TITLE-ABS ("health service coverage") OR TITLE-ABS ("scope of health care") OR TITLE-ABS ("coverage of health care") OR TITLE-ABS ("coverage of health service")) AND (LIMIT-TO (PUBSTAGE , "final")) AND (LIMIT-TO (PUBYEAR , 2021) OR LIMIT-TO (PUBYEAR , 2020) OR LIMIT-TO (PUBYEAR , 2019) OR LIMIT-TO (PUBYEAR , 2018) OR LIMIT-TO (PUBYEAR , 2017) OR LIMIT-TO (PUBYEAR , 2016) OR LIMIT-TO (PUBYEAR , 2015) OR LIMIT-TO (PUBYEAR , 2014) OR LIMIT-TO (PUBYEAR , 2013)) AND (LIMIT-TO (DOCTYPE , "ar")) AND (LIMIT-TO (SUBJAREA , "MEDI") OR LIMIT-TO (SUBJAREA , "NURS")) AND (LIMIT-TO (LANGUAGE , "English")) AND (LIMIT-TO (SRCTYPE , "j")) AND (LIMIT-TO (SRCTYPE , "j")) AND (AFFIL (".au") OR AFFIL ("australia*") OR AFFIL ("new south wales") OR AFFIL ("victoria") OR AFFIL ("queensland") OR AFFIL ("south australia") OR AFFIL ("western australia") OR AFFIL ("tasmania") OR AFFIL ("australian capital territory") OR AFFIL ("northern territory"))	1,700
#4	(TITLE-ABS ("continuity of patient care") OR TITLE-ABS ("continuity of care") OR TITLE-ABS ("healthcare continuum") OR TITLE-ABS OR TITLE-ABS ("continuum of care") OR TITLE-ABS ("healthcare continuum")) AND (LIMIT-TO (PUBSTAGE , "final")) AND (LIMIT-TO (PUBYEAR , 2021) OR LIMIT-TO (PUBYEAR , 2020) OR LIMIT-TO (PUBYEAR , 2019) OR LIMIT-TO (PUBYEAR , 2018) OR LIMIT-TO (PUBYEAR , 2017) OR LIMIT-TO (PUBYEAR , 2016) OR LIMIT-TO (PUBYEAR , 2015) OR LIMIT-TO (PUBYEAR , 2014) OR LIMIT-TO (PUBYEAR , 2013)) AND (LIMIT-TO (DOCTYPE , "ar")) AND (LIMIT-TO (SUBJAREA , "MEDI") OR LIMIT-TO (SUBJAREA , "NURS")) AND (LIMIT-TO (LANGUAGE , "English")) AND (LIMIT-TO (SRCTYPE , "j")) AND (LIMIT-TO (SRCTYPE , "j")) AND (AFFIL (".au") OR AFFIL ("australia*") OR AFFIL ("new south wales") OR AFFIL ("victoria") OR AFFIL ("queensland") OR AFFIL ("south australia") OR AFFIL ("western australia") OR AFFIL ("tasmania") OR AFFIL ("australian capital territory") OR AFFIL ("northern territory"))	12,626
#5	(TITLE-ABS ("quality of health care") OR TITLE-ABS ("quality of care") OR TITLE-ABS ("quality of health service") OR TITLE-ABS ("input quality") OR TITLE-ABS ("process quality") OR TITLE-ABS ("safe healthcare") OR TITLE-ABS ("effective healthcare") OR TITLE-ABS ("efficient health care")) AND (LIMIT-TO (PUBSTAGE , "final")) AND (LIMIT-TO (PUBYEAR , 2021) OR LIMIT-TO (PUBYEAR , 2020) OR LIMIT-TO (PUBYEAR , 2019) OR LIMIT-TO (PUBYEAR , 2018) OR LIMIT-TO (PUBYEAR , 2017) OR LIMIT-TO (PUBYEAR , 2016) OR LIMIT-TO (PUBYEAR , 2015) OR LIMIT-TO (PUBYEAR , 2014) OR LIMIT-TO (PUBYEAR , 2013)) AND (LIMIT-TO (DOCTYPE , "ar")) AND (LIMIT-TO (SUBJAREA , "MEDI") OR LIMIT-TO (SUBJAREA , "NURS")) AND (LIMIT-TO (LANGUAGE , "English")) AND (LIMIT-TO (SRCTYPE , "j")) AND (LIMIT-TO (SRCTYPE , "j")) AND (AFFIL (".au") OR AFFIL ("australia*") OR AFFIL ("new south wales") OR AFFIL ("victoria") OR AFFIL ("queensland") OR AFFIL ("south australia") OR AFFIL ("western australia") OR AFFIL ("tasmania") OR AFFIL ("australian capital territory") OR AFFIL ("northern territory"))	56,240
#6	(TITLE-ABS ("person centred care") OR TITLE-ABS ("client centred care") OR TITLE-ABS ("individualised care") OR TITLE-ABS ("responsive care") OR TITLE-ABS ("client satisfaction") OR TITLE-ABS ("person centeredness") OR TITLE-ABS ("person-centeredness") OR TITLE-ABS ("client centeredness")) AND (LIMIT-TO (PUBSTAGE , "final")) AND (LIMIT-TO (PUBYEAR , 2021) OR LIMIT-TO (PUBYEAR , 2020) OR LIMIT-TO (PUBYEAR , 2019) OR LIMIT-TO (PUBYEAR , 2018) OR LIMIT-TO (PUBYEAR , 2017) OR LIMIT-TO (PUBYEAR , 2016) OR LIMIT-TO (PUBYEAR , 2015) OR LIMIT-TO (PUBYEAR , 2014) OR LIMIT-TO (PUBYEAR , 2013)) AND (LIMIT-TO (DOCTYPE , "ar")) AND (LIMIT-TO (SUBJAREA , "MEDI") OR LIMIT-TO (SUBJAREA , "NURS")) AND (LIMIT-TO (LANGUAGE , "English")) AND (LIMIT-TO (SRCTYPE , "j")) AND (LIMIT-TO (SRCTYPE , "j")) AND (AFFIL (".au") OR AFFIL ("australia*") OR AFFIL ("new south wales") OR AFFIL ("victoria") OR AFFIL ("queensland") OR AFFIL ("south australia") OR AFFIL ("western australia") OR AFFIL ("tasmania") OR AFFIL ("australian capital territory") OR AFFIL ("northern territory"))	6,786
#7	(TITLE-ABS ("coordinated health care") OR TITLE-ABS ("coordinated health service") OR TITLE-ABS ("integrated health service") OR TITLE-ABS ("multi-sectoral collaboration") OR TITLE-ABS ("multi sectoral collaboration") OR TITLE-ABS ("intra-sectoral collaboration") OR TITLE-ABS ("intra sectoral collaboration") OR TITLE-ABS ("trans-sectoral collaboration") OR TITLE-ABS ("trans sectoral collaboration") OR TITLE-ABS ("public-private partnership") OR TITLE-ABS ("public private partnership") OR TITLE-ABS ("community involvement") OR TITLE-ABS ("community participation") OR TITLE-ABS ("Centrelink") OR TITLE-ABS ("Centre link")) AND (LIMIT-TO (PUBSTAGE , "final")) AND (LIMIT-TO (PUBYEAR , 2021) OR LIMIT-TO (PUBYEAR , 2020) OR LIMIT-TO (PUBYEAR , 2019) OR LIMIT-TO (PUBYEAR , 2018) OR LIMIT-TO (PUBYEAR , 2017) OR LIMIT-TO (PUBYEAR , 2016) OR LIMIT-TO (PUBYEAR , 2015) OR LIMIT-TO (PUBYEAR , 2014) OR LIMIT-TO (PUBYEAR , 2013)) AND (LIMIT-TO (DOCTYPE , "ar")) AND (LIMIT-TO (SUBJAREA , "MEDI") OR	24,942

	LIMIT-TO (SUBJAREA , "NURS")) AND (LIMIT-TO (LANGUAGE , "English")) AND (LIMIT-TO (SRCTYPE , "j")) AND (LIMIT-TO (SRCTYPE , "j")) AND (AFFIL (".au") OR AFFIL ("australia*") OR AFFIL ("new south wales") OR AFFIL ("victoria") OR AFFIL ("queensland") OR AFFIL ("south australia") OR AFFIL ("western australia") OR AFFIL ("tasmania") OR AFFIL ("australian capital territory") OR AFFIL ("northern territory"))	
#8	(TITLE-ABS ("accountability in health care") OR TITLE-ABS ("accountability in health system") OR TITLE-ABS ("reporting in health care") OR TITLE-ABS ("transparency in health care") OR TITLE-ABS ("transparency in health system") OR TITLE-ABS ("reward in health system") OR TITLE-ABS ("reward in health care") OR TITLE-ABS ("punishment in health system") OR TITLE-ABS ("monitoring and evaluation in health system") OR TITLE-ABS ("monitoring and evaluation in health system")) AND (LIMIT-TO (PUBSTAGE , "final")) AND (LIMIT-TO (PUBYEAR , 2021) OR LIMIT-TO (PUBYEAR , 2020) OR LIMIT-TO (PUBYEAR , 2019) OR LIMIT-TO (PUBYEAR , 2018) OR LIMIT-TO (PUBYEAR , 2017) OR LIMIT-TO (PUBYEAR , 2016) OR LIMIT-TO (PUBYEAR , 2015) OR LIMIT-TO (PUBYEAR , 2014) OR LIMIT-TO (PUBYEAR , 2013)) AND (LIMIT-TO (DOCTYPE , "ar")) AND (LIMIT-TO (SUBJAREA , "MEDI") OR LIMIT-TO (SUBJAREA , "NURS")) AND (LIMIT-TO (LANGUAGE , "English")) AND (LIMIT-TO (SRCTYPE , "j")) AND (LIMIT-TO (SRCTYPE , "j")) AND (AFFIL (".au") OR AFFIL ("australia*") OR AFFIL ("new south wales") OR AFFIL ("victoria") OR AFFIL ("queensland") OR AFFIL ("south australia") OR AFFIL ("western australia") OR AFFIL ("tasmania") OR AFFIL ("australian capital territory") OR AFFIL ("northern territory"))	146
#9	(TITLE-ABS ("primary health care") OR TITLE-ABS ("community mental health services") OR TITLE-ABS ("family practice") OR TITLE-ABS ("home care services") OR TITLE-ABS ("family physicians") OR TITLE-ABS ("community health services") OR TITLE-ABS ("community health nursing") OR TITLE-ABS ("community pharmacy services") OR TITLE-ABS ("community health workers") OR TITLE-ABS ("preventive health services") OR TITLE-ABS ("primary care") OR TITLE-ABS ("general practi*") OR TITLE-ABS ("primary health*") OR TITLE-ABS ("community mental health*") OR TITLE-ABS ("family practice") OR TITLE-ABS ("family medicine") OR TITLE-ABS ("family physician*") OR TITLE-ABS ("home care") OR TITLE-ABS ("home based") OR TITLE-ABS ("home health*") OR TITLE-ABS ("community health*") OR TITLE-ABS ("community nurs*") OR TITLE-ABS ("health visit*") OR TITLE-ABS ("community pharmac*") OR TITLE-ABS ("preventive care") OR TITLE-ABS ("prevention program*") OR TITLE-ABS ("preventive service*") OR TITLE-ABS ("preventive health") OR TITLE-ABS ("health promotion")) AND (LIMIT-TO (PUBSTAGE , "final")) AND (LIMIT-TO (PUBYEAR , 2021) OR LIMIT-TO (PUBYEAR , 2020) OR LIMIT-TO (PUBYEAR , 2019) OR LIMIT-TO (PUBYEAR , 2018) OR LIMIT-TO (PUBYEAR , 2017) OR LIMIT-TO (PUBYEAR , 2016) OR LIMIT-TO (PUBYEAR , 2015) OR LIMIT-TO (PUBYEAR , 2014) OR LIMIT-TO (PUBYEAR , 2013)) AND (LIMIT-TO (DOCTYPE , "ar")) AND (LIMIT-TO (SUBJAREA , "MEDI") OR LIMIT-TO (SUBJAREA , "NURS")) AND (LIMIT-TO (LANGUAGE , "English")) AND (LIMIT-TO (SRCTYPE , "j")) AND (AFFIL (".au") OR AFFIL ("australia*") OR AFFIL ("new south wales") OR AFFIL ("victoria") OR AFFIL ("queensland") OR AFFIL ("south australia") OR AFFIL ("western australia") OR AFFIL ("tasmania") OR AFFIL ("australian capital territory") OR AFFIL ("northern territory"))	475,169
#10	("#1" OR "#2" OR "#3" OR "#4" OR "#5" OR "#6" OR "#7" OR "#8")	39,404,885
#11	("#10") AND ("#9") AND AND (LIMIT-TO (SRCTYPE,"j")) AND (LIMIT-TO (OA,"all")) AND (LIMIT-TO (AFFILCOUNTRY,"Australia")) AND (LIMIT-TO (DOCTYPE,"ar")) AND (LIMIT-TO (SUBJAREA,"MEDI") OR LIMIT-TO (SUBJAREA,"NURS")) AND (LIMIT-TO (PUBYEAR,2021) OR LIMIT-TO (PUBYEAR,2020) OR LIMIT-TO (PUBYEAR,2019) OR LIMIT-TO (PUBYEAR,2018) OR LIMIT-TO (PUBYEAR,2017) OR LIMIT-TO (PUBYEAR,2016) OR LIMIT-TO (PUBYEAR,2015) OR LIMIT-TO (PUBYEAR,2014) OR LIMIT-TO (PUBYEAR,2013)) AND (LIMIT-TO (LANGUAGE,"English")) AND (EXCLUDE (EXACTKEYWORD,"Systematic Review") OR EXCLUDE (EXACTKEYWORD,"Meta Analysis"))	4,465

Web of science search strategy

No.	Query	Results
#1	(TS= (comprehensive health care) OR TS= (preventative care) OR TS= (preventative care) OR TS= (curative care) OR TS= (palliative care) OR TS= (rehabilitat* care) OR TS= (health promotion) OR TS= (comprehensive health service) OR TS= (inclusive health care) OR TS= (inclusive health service) OR TS= (inclusive health service) OR TS= (inclusivity of health care))	220,195
#2	(TS= (health service accessibility) OR TS= (health care access) OR TS= (health care accessibility) OR TS= (access to health care) OR TS= (access to health service) OR TS= (healthcare disparity) OR TS= (medically underserved area) OR TS= (medical indigence))	131,120
#3	(TS= (health care coverage) OR TS= (health service coverage) OR TS= (scope of health care) OR TS= (coverage of health care) OR TS= (coverage of health service))	43,075
#4	(TS= (continuity of patient care) OR TS= (continuity of care) OR TS= (healthcare continuum) OR TS= (continuum of care) OR TS= (healthcare continuum))	24,347
#5	(TS= (quality of health care) OR TS= (quality of care) OR TS= (quality of health service) OR TS= (input quality) OR TS= (process quality) OR TS= (safe healthcare) OR TS= (effective healthcare) OR TS= (efficient health care))	1,015,698
#6	(TS= (person centred care) OR TS= (client centred care) OR TS= (individualised care) OR TS= (responsive care) OR TS= (client satisfaction) OR TS= (person centeredness) OR TS= (person-centeredness) OR TS= (client centeredness))	48,883
#7	(TS= (coordinated health care) OR TS= (coordinated health service) OR TS= (integrated health service) OR TS= (multi-sectoral collaboration) OR TS= (multi sectoral collaboration) OR TS= (intra-sectoral collaboration) OR TS= (inra sectoral collaboration) OR TS= (trans-sectoral collaboration) OR TS= (trans sectoral collaboration) OR TS= (public-private partnership) OR TS= (public private partnership) OR TS= (community involvement) OR TS= (community participation) OR TS= (Centrelink) OR TS= (Centre link))	219,473
#8	(TS= (accountability in health care) OR TS= (accountability in health system) OR TS= (reporting in health care) OR TS= (transparency in health care) OR TS= (transparency in health system) OR TS= (reward in health system) OR TS= (reward in health care) OR TS= (punishment in health system) OR TS= (monitoring and evaluation in health system) OR TS= (monitoring and evaluation in health system))	196,570
#9	(TS= (primary health care) OR TS= (community mental health services) OR TS= (family practice) OR TS= (home care services) OR TS= (family physicians) OR TS= (community health services) OR TS= (community health nursing) OR TS= (community pharmacy services) OR TS= (community health workers) OR TS= (preventive health services) OR TS= (primary care) OR TS= (general practi*) OR TS= (primary health*) OR TS= (community mental health*) OR TS= (family practice) OR TS= (family medicine) OR TS= (family physician*) OR TS= (home care) OR TS= (home based) OR TS= (home health*) OR TS= (community health*) OR TS= (community nurs*) OR TS= (health visit*) OR TS= (community pharmac*) OR TS= (preventive care) OR TS= (prevention program*) OR TS= (preventive service*) OR TS= (preventive health) OR TS= (health promotion))	1,547,531
#10	(((((((#1) OR #2) OR #3) OR #4) OR #5) OR #6) OR #7) OR #8	1,592,043
#11	(#10) AND #9	429,505
#12	(#10) AND #9 and 2013 or 2014 or 2015 or 2016 or 2017 or 2018 or 2019 or 2020 or 2021 (Publication Years) and AUSTRALIA (Countries/Regions) and English (Languages) and Review Articles (Exclude – Document Types) and Articles (Document Types) and Open Access	11,452

Supplementary tables

STable 1. Characteristics of studies reported accessibility and barriers to PHC access

Author (year)	Jurisdiction	Duration of the study	Study type/design	Study participants	Study sample (n)	Key findings	Conclusions	WHO good PHC attribute assessed
Bartlem, K., et al. (2015) (53)	New South Wales	December 2011 to November 2012	Cross – sectional study	community mental health service clients	- 558 mental health clients	- Chronic disease preventive care was highly acceptable to mental health clients (86%-97%). However, receipt of preventive care was low.<br>- The proportion of clients at risk of and assessed for unhealthy behaviour who then received brief advice ranged from 69% to 85%, whereas only 38% to 49% received any referral.<br>- A greater number of mental health appointments were associated with higher prevalence of preventive care for chronic diseases.	- Practice change strategies are required to increase the delivery of routine preventive care within mental health services if clients are to benefit from clinical guidelines.	Acceptability of care
Bartlem, K., et al. (2019) (90)	New South Wales	2009 to 2014	Survey	Client attending community health care	- 5,639 community health care clients	- The receiving chronic disease preventive care is associated with higher odds of behaviour change<br>- However, assessment of smoking and advise toward prevention of chronic disease was not associated with receipt preventive care	- Results highlight the importance of primary care clinicians providing best practice preventive care to maximise client behaviour change	Acceptability of care
Brodribb, W. E., et al. (2016) (54)	Queensland	February to July 2013	Cross – sectional study	- General practices in the catchment providing maternal and child health services	497 general practices	- Although 80% of practice nurses in GP services saw mothers and infants, only 30% had midwifery or child health training.<br>- Out-of-pocket expenses for MCH services ranged from $10-$60.	- There are several practice related factors that could positively impact on postpartum care.<br>- There are no financial impediments to women accessing care.<br>- Some factors can easily be adapted within practices.	Access to care
Yelland, J., et al. (2016) (71)	South Australia	July 2011 to June 2013	Cross – sectional study	- Women 4-9 months after giving birth	- 344 women	- The majority had seen a primary health care practitioner since the birth: 86% had seen a Child and Family Health Service (CaFHS) nurse, 81% a general practitioner (GP), and 61% an Aboriginal health worker (AHW)<br>- Women living in remote areas were more likely to have seen primary care practitioners than women living in Adelaide	- Despite high prevalence of maternal and infant morbidity, a sizeable woman did not access primary care practitioners in their postpartum period. Stronger efforts are needed to ensure Aboriginal women and families receive appropriate postnatal follow-up	Access to healthcare (GPs)
Carman, R., et al. (2019) (83)	West Australia	February to November 2018	Mixed-method study	- GPs providing Aboriginal children vaccination services	- 316 GPs and 3 Key Informants	- Of the total GPs, 67.4% were unaware of the low vaccination coverage in Aboriginal children; 64.8% had not received cultural sensitivity training in their workplace and 46.8% reported having inadequate time to follow up overdue child vaccinations.	- There is inadequate awareness of the current rates and vaccination uptake in Aboriginal children<br>- Cultural safety is a critical component of the acceptability and accessibility of services; lack of awareness may restrict the development of strategies designed to equitably address low coverage vaccination in aboriginal children.	Accessibility and acceptability
Dasgupta, P., et al. (2021) (122)	Queensland	2013 to 2017	Population based	- Women, aged 20–69 years who underwent cervical screening	1,107,233 women	- Disparities in cancer screening care among aboriginal and non-aboriginal women was observed. But this difference was attenuated when further adjusted for relevant confounders	- Improving access to primary health care for Aboriginal through ACCHOs, may reduce existing disparities in cervical screening participation<br>- Further gains will require greater levels of local community engagement and understanding of the experiences of	Access to health care

							screened women to inform effective interventions	
Hegarty, K., et al. (2013) (72)	Victoria	2006 to 2007	Mixed method	- Purposive sampling of PNs, GPs and administrative staff from regional and rural general practices	- 3 GPs in rural and regional areas of Victoria providing nurse-led youth health clinics	- Nurse – led GP clinics were poorly attended by young people and several barriers affecting clinic attendance including the short timeframe of the study, set times of the clinics and a lack of support for the clinics by some GPs and external youth health clinics, resulting in few referrals<br>- The clinics cost from $5912 to $8557 to establish, which included training the practice nurses.	- The implementation of youth health clinics is not feasible in a short timeframe and to maximise use of the clinics, all members of the GPs team need to find the clinics acceptable.	Acceptability of care and cost
Hoang, H., et al. (2014) (85)	Tasmania	May to September 2010	Mixed-method study	- Women from six rural communities studied	- 210 women for the quantitative part<br>- 22 for the interview	- Majority of rural women believed antenatal education, antenatal, postnatal check-ups and maternity units should be provided locally including in local hospitals<br>- Lack of access and difficult of accessing maternity care for rural women were emerged as main themes.	- The study supports the claim that the closure of rural maternity units shifts cost and risk from the health care system to rural women and their families.	Access to health care
Inacio, M. C., et al. (2020) (99)	National	July 2003 to Jun 2013	Retrospective cohort study	- None-Aboriginal or Torres Strait Islander aged population (≥65 years) who had a first-time aged care eligibility assessment by an Aged Care Assessment Team (ACAT), and were approved for permanent residential, home, respite, or transition care	- 799 750 older Australians	- Incidence of accessing aged care in the last 1 year was:<br>- Permanent residential care 70.9% (95%CI:70.8%-71.0%)<br>- Home care 49.5% (95% CI 49.3%-49.7%)<br>- Respite care 41.8% (95% CI 41.7%-41.9%).<br>- Transition care within 28 days was 78.5% (95% CI: 78.2%-78.7%)<br>- The cumulative incidence of accessing the four approved aged care services ranges between 42% and 79% with 22% to 28% of aged individuals approved for aged care services did not access to aged care.	- Monitoring the use of aged care service approvals to identify service access barriers can support ongoing evidence-based policy changes	Access to approved aged care services
James, S., et al. (2018) (36)	Queensland	2015	Qualitative study (Critical grounded theory framework)	- Aboriginal women from urban Aboriginal and Torres Strait Islander Community-Controlled Health Care Organisation (ATSICCHO)	- 17 Aboriginal women aged least 16 years and within 12 months of postpartum	- Aboriginal women seek access to postpartum contraception and control over family planning<br>- Social factors such as embarrassment and low health literacy level in Aboriginal women are barriers to use PHC services for contraception	- While health professional at PHC play key role in dismantling barriers to access and use of PHC for reproductive health, more research is needed concerning Aboriginal and Torres Strait Islander women	Access and utilisation
McElwaine, K. M., et al. (2013) (65)	New South Wales	Nov 2009 to October 2010	Cross – sectional study	- Population in 56 public community health facilities in 1 year	- 1284 clients (from a network of 56 public community health facilities)	- Preventive care was most frequently reported for smoking (assessment: 59.9%, brief advice: 61.7%, and offer of referral to a telephone service: 4.5%) and least frequently for inadequate fruit or vegetable consumption (27.0%, 20.0% and 0.9% respectively).<br>- Preventive care assessment for all risks factors was only 16.2%<br>- Acceptability of preventive care at PHC ranges from 76.0%-95.3%<br>- Socially advantaged people, being male, Aboriginal, unemployed, and socio-economically disadvantaged were more likely to be assessed for preventive risk factors	- Despite strong client support, preventive care was not provided opportunistically to all, and was preferentially provided to selected groups implying that the need for practice change strategies to enhance preventive care improvements	Acceptability of care<br>Disparity of preventive health care
Munns, A. (2021) (74)	Western Australian	2013	Mixed method	- Community Midwives (CMs) and their manager,	- Interviews with 19 providers (CMs, child	- Culturally safe community midwifery primary health care is a strong enabling	- Sensitively designed, incorporating flexible PHC approaches with the	Accessibility<br>Collaboration

				and an Aboriginal Support Worker	health nurses, program managers, a liaison officer, doctors, and community agency staff	factor, with potential to reduce health disparities during pregnancy for Aboriginal and Torres Strait Islander women	involvement of community midwifery, health care managers and Aboriginal Support Workers increase accessibility, collaboration, and sustainability of antenatal care for Aboriginal and Torres Strait Islander women	Sustainability
Munns, A., et al. (2016) (75)	Western Australia	2010 to 2012	Mixed method	- Program and supporting agency staff	- 22 participants	- The Aboriginal Community Antenatal Program (ACAP) show acceptability and satisfaction in provision of community-based pre-conception and antenatal health services and enhancing collaboration between a range of health provider agencies, with partnerships between Aboriginal and non-Aboriginal program staff contributing to an emerging model of community antenatal care.	- The Aboriginal Community Antenatal Program has potential to be an ongoing community-based model of care for preconception and antenatal clients in a remote Australian region	Acceptability and satisfaction
Parker, R., et al. (2013) (44)	ACT, QLD, NSW, WA, VIC	August to September 2010	Qualitative study	- Primary health consumers	- 77 participants	- The role of nurse practitioners in PHC is positively evaluated by the consumers with some role confusion with other primary health care nurses.<br>- Participants in the focus groups were very positive about nurse practitioners and would find them acceptable in providing primary health care.	- The introduction of nurse practitioners to the primary health care workforce offers the potential of increased access for consumers, particularly if nurse practitioners can be enticed to work in areas where the GP workforce is reduced such as rural and remote areas.	Acceptability of PHC nurses
Raymundo, G., et al. (2021) (45)	New South Wales	2018	Qualitative study	- Young adults between 18-24 years from migrant backgrounds	- 25 participants (20 semi-structured individual interviews and one group interview with five participants)	- Comprehensive education about accessing health services for young people, as well as health promotion strategies targeting communities and families are needed to overcome health service access barriers faced by young adult migrants	- More effective delivery of health services information in education, positive engagement between service-providers and service users, and age-appropriate, culturally considerate health promotion strategies are needed to overcome barriers to health services accessibility	Access to health care
Reifels, L., et al. (2015) (132)	National	2003 to 2013	NS	- Indigenous Clients visited Medicare Locals mental health for prevention	- 15,450 Indigenous mental health and suicide prevention clients from 58 Medicare Locals and 16 Medicare Locals involved in the Indigenous suicide prevention service during the 2011-12 and 2012-13 financial years	- Non-Indigenous ATAPS service volume of primary care agencies was uniquely predictive of Indigenous service reach.	- Enhanced mainstreaming of primary mental healthcare programs can result in significant gains in access to mental healthcare for Indigenous populations	Access to health care
Slimings, C. and M. Moore (2021) (128)	New South Wales		Ecological study	- Rural local government health service providers	- 89 local government areas in rural areas comprising (47 inner regional, 33 outer regional, 6 remote and 3 very remote areas)	- There was large geographic variation in health service performance for avoidable mortality and potentially preventable hospital admissions<br>- The average annual avoidable age-standardised mortality rate ranged from 78.1 – 493.7 per 100 000 population and high rate of potentially preventable hospitalisations<br>- The least geographic variation was seen for immunisation coverage.<br>- The most common explanations for variation between rural local government areas in New	- Striking differences in health system performance indicators were found among New South Wales rural local government areas that were not solely due to remoteness<br>- There are geographic disparities in health service performance indicators – with the existing large rural and Indigenous inequities in mortality, morbidity, and potentially preventable hospitalisations	Accessibility (health care disparity)

						South Wales were remoteness and socioeconomic characteristics		
Tennett, D., et al. (2020) (100)	Queensland	January 2017 to December 2017	Retrospective cohort study	- Women birthing in 21 GP-obstetrician supported birthing units in rural maternity units	3111 Women birthing in rural maternity units	- Women birthing in rural maternity units were more likely to be Aboriginal or Torrs Strait Islander<br>- Neonatal outcomes were comparable, with no significant difference in stillbirth rate between rural units and all Queensland public hospitals<br>- Precipitate delivery was the most common labour complication (36% v 33%) ($p<0.001$)	- GP-obstetrician (rural generalist) supported rural birthing units in Queensland provide important access for low and medium risk women to deliver locally, with strong indicators of quality and safety	Access to health care
Wood, A., et al. (2020) (102)	Northern Territory	2013–2014	Retrospective cohort study	- Pregnant women with hyperglycaemia	195 women with hyperglycaemia in pregnancy	- Only 54% with GDM had postpartum glycaemic check<br>- Most women (97%) accessed a PHC unit (health centre) at least once in the 12-month postpartum period but during these visits, only 52% of women had service provision, either structured or opportunistic, related to diabetes.	- While there is high rate of postpartum DM care access, many women did not attend health services for diabetes screening or management. This suggests that still high need for increased access in screening and early intervention at PHC level to prevent the development of T2D and its complications.	Access to PHC in the context of diabetic care during pregnancy
Zurynski, Y., et al. (2021) (96)	National	2018	Survey	- Randomly selected people aged > 18 years	1024 randomly selected people (432 males, 592 females)	- While GP are the most accessed services and the proportion of people with chronic conditions is growing, there is a significant affordability problem when accessing needed healthcare for people with chronic disease<br>- There is also a significant healthcare cost barrier to access services manifested in respondents reporting skipping medication doses and pathology tests, and foregoing appointments with doctors because of cost.	- Providing accessible and affordable PHC services will be critical to meet ongoing demand and to reduce pressure on public hospitals.<br>- Equitable access to healthcare must be at the centre of health reform	Accessibility and affordability of healthcare<br>Health care cost
Mu, C., and J. Hall (2020) (67)	National	2009 to 2010	Cross – sectional study	- Use of general practices (GPs) measured by the number of GP visits per capita in	- 756 Statistical Local Areas (SLAs)	- The use of GPs influenced by demand-side and supply-side factors accounted for 32.24% of the regional differences<br>- GP visits are more frequent in urban regions, areas with a higher percentage of the population who are obese, who have profound or severe disability, and who hold concession cards, and areas with a smaller percentage of the population who reported difficulty in accessing services	- There was substantial variation in GP use across Australian regions with only a small proportion of them being explained by population health needs, indicating a high level of unexplained clinical variation.	Regional variation in GP service
Mazumdar, S., et al. (2014) (93)	New South Wales	2006 to 2009 linked to 2002 to 2010	Survey	- Patient population	267,153 patients	- The median percentage of patients that obtain their PHC is 55% with localization increasing with rurality implicating that Australian PHC has an immense potential to be of value to policymakers and researchers	- Patients in NSW will travel to obtain GP care for many reasons	Geographic accessibility
Inder, K. J., et al. (2012) (61)	New South Wales	2007 to 2009	Cross – sectional study	- Community dwelling residents participated in in the Australian Rural Mental Health Study	- 2639 individuals (representing 1879 households)	- There was higher professional mental health help seeking behaviour in this rural population exceeded the national rate (17% vs.11.9%).<br>- Higher levels of help seeking were associated with the absence of a partner, poorer finances, severity of mental health problems, and higher levels of adversity.<br>- Remoteness was associated with lower utilization of non-professional support.	- There were higher rates of GP attendance for mental health problems for rural community cohort compared with data from metropolitan centres.<br>- Geographic and financial barriers to service use were identified and perception of service adequacy was relatively low, especially among those with the highest levels of distress and greatest adversity.	Geographic and financial barriers

						- Nevertheless, 47% of those with estimated high service need had no contact with professional services.		
Pearson, O., et al. (2020) (25)	National	2017 to 2018	Qualitative report analysis	- ACCHO member services of the National Aboriginal Community Controlled Health Organisation	- 67 ACCHO member services	- 53% reports from ACCHO showed that all annual reports described working to improve socioeconomic position and intermediary determinants of health<br>- Culture had a strong presence in program delivery and building social cohesion and social capital emerged as themes.	- As a PHC provider, ACCHO sector played an essential role in addressing social and structural determinants of health and health inequity experienced by Indigenous communities.<br>- The ACCHO efforts had accumulative positive effect in closing the gap towards health equity.	Health equity
Henderson, J., et al. (2018) (34)	National	May to August 2016	Qualitative study	Senior staff, board members and members of clinical and community advisory councils or health priority groups	- 55 stakeholders from 6 PHN (comprised of 23 senior staff of the PHNs, 11 board members and 21 members of clinical and community advisory councils or health priority groups)	- PHN is a commissioning organisation in PHC planning. However, in this study Service delivery was seen as fragmented, the model is at odds with the way Aboriginal Community Controlled Health Organisations (ACCHOs) operate and rural regions lack services to commission.	- Consequently, reliance upon commissioning of services may not be appropriate for the Australian primary healthcare context.<br>- Commissioning may inhibit service equity in Australia as responsibility for service delivery is fragmented, appears particularly inappropriate when Aboriginal community-controlled services are available and in rural regions which lack services to commission.	Healthcare equity
Freeman, T., et al. (2016) (17)	South Australia and Northern Territory	2009 to 2013	Qualitative study	- 6 Australian PHC services (5 in South Australia and 1 in the Northern Territory), were followed over 5 years	- 55 interviews with<br>- Service managers<br>- Staff, regional health executives and health department representatives	- Changing policy environment over 5 years, has compromised equity of access and services' scope to facilitate access to other health services	- Understanding the value of community governance and need to monitor equity performance and advocate for the importance of health equity are important to ensure PHC access and equity.	Health inequity/equity of healthcare access
Rolfe, M. I., et al. (2017) (127)	New South Wales, Victoria, Queensland, South Australia, West Australia, Tasmania, and NT	2005 to 2010	Ecological study	- 259 health facilities	259 health facilities	- Population factors relating to vulnerability and isolation did not increase the likelihood of a local birthing facility, and very remote communities were less likely to have any service.	- Birthing service in Australia shows disparities between rural and remote distribution<br>- Birthing services are also influenced by jurisdictions	Healthcare disparities
Callander, E., et al. (2017) (131)	National	2014 to 2015	NS	- Out-of-pocked cost dada of clients used in selected PHC	NA	- Out-of-pocket PHC care cost for allied health service is significantly higher in regional areas than PHCs in capital cities<br>- Some areas had both high PHC out-of-pocket charges and disadvantaged populations	- There was a large variation in out-of-pocket charges for PHC between PHN in Australia. But there is a little evidence of health inequality across age and socio-demographic characteristics including Aboriginal and Torres Strait Islander people	Affordability in PHC cost
Thomas, S. L., et al. (2014) (101)	Northern Territory	January 2002 to 31 December 2011	Retrospective cohort study	- Indigenous NT residents (> 5 years) with diabetes	4 184 patients from 5 hospitals or 54 remote clinics in the NT	- Compared with the low PHC use group, the medium-use group (patients who used PHC 2–11 times annually) had lower rates of hospitalisation, lower potentially avoidable hospitalisation (PAH), lower death rates and fewer YLL.<br>- The cost of preventing diabetes hospitalisation is directly associated with the number of PHC use with average cost of $2915 per one hospitalisation.	- Improving access to primary care in remote communities for the management of diabetes results in net health benefits to patients and cost savings to government	Cost-effectiveness
Quach, J., et al. (2014) (95)	National	2004	Survey	- Parent-reported that their children had Special Health	- 5107 children aged 0–1 year	- The proportion of children with special health care need (SHCN) increased from 6.1% at age 0–1 year to 15.0% at age 6–7 years with	- Special health care need (SHCN) incur substantial non-hospital costs to Medicare.	Economic and cost effectiveness

				Care Needs screened at two and their controls	- 4983 children aged 4–5 years	associated additional health care costs ranged from$491 per child at 6–7 years to $1202 at 0–1 year.<br>- This equates to an additional $161.8 million annual cost or 0.8% of federal funding for non-hospital-based health care.	- The difference in primary health-care costs alone during the first 8 years of life accounted for 0.8% of the overall non-hospital-based Medicare expenditure	
Spaeth, B. A., et al. (2018) (133)	Northern Territory	2015	NS	- 6 remote PHC services that have access to on-site point of care test (POCT) to diagnose 3 common acute medical conditions	- 200 patients from 6 remote PHC services	- Use of point of care testing tool (POCT) prevented 60 unnecessary medical evacuations with a significant cost savings (AUD $786 to $8,034) per patient depending on the medical conditions	- When used to aid decision making for acutely ill patients delivered significant cost savings for the NT health care system by preventing unnecessary emergency medical retrievals.	Economic evaluation in rural health
James, S., et al. (2021) (79)	Capital Territory and South-East New South Wales	August 2017 to March 2018	Mixed methods	GP nurses working in both rural and metropolitan settings	- 15 registered nurses from 14 GPs	- Although communication about lifestyle risk factors is within the general practice nurses' scope of practice, concerted efforts fostering interdisciplinary collaboration, the prioritisation of time, funding and educational opportunities would better support this role, at the same time optimising chronic disease management and patient outcomes.	- Communicating with patients about lifestyle risk reduction is a key aspect of GPNs' role in health promotion and chronic disease management. However, there are several barriers and competing clinical and professional issues to be addressed in policy and practice level.	Barriers and facilitators of risk communication
Liaw, S. T., et al. (2019) (116)	New South Wales, Victoria	April 2014 – May 2017	Randomised controlled trial	General practices, patients and staffs working in GPs	- 942 patients from 28 intervention practices consisting of 135 GPs, 807 Indigenous patients and<br>- 1754 patients from 28 control practices consisting of 210 GPs, 1554 Indigenous patients	- Regardless of practice attributes and professional groups, cultural respect in general practice is not associated with increased Indigenous health checks or improve cultural respect scores	- Conceptual, methodologic, and contextual factors that influence cultural mentorship, culturally respectful clinical practice, and Indigenous health care require further investigation	Cultural respect in general practice
Freeman, T., et al. (2014) (30)	South Australia	2009 to 2011.	Qualitative study	- PHC personnel	- 22 interviews with staff from both<br>- 21 clients from 4 community assessment workshops	- Staffs and clients reported positive achievements in addressing health determinant<br>- However, communication difficulties; racism and discrimination; and externally developed programs are barriers in addressing social determinants	- Service-level strategies were necessary to achieving cultural respect.<br>- These strategies have the potential to improve Aboriginal and Torres Strait Islander health and wellbeing	Cultural respect in PHC
Wyndow, P., et al. (2020) (50)	Western Australia	February 2017 to November 2017	Qualitative study	- Aboriginal women including (smokers, non-smokers, and ex-smokers) health professionals and other relevant organisations	- 25 interviews with Aboriginal women participated in focus groups or individual interviews	- Women who stopped smoking did so on their own when the reason was important enough or when they saw alternative ways of living.<br>- Creating safe places to bring women together to YARN about women's business and link with health services was identified as critical to support women to stop smoking	- Connection to Aboriginal culture to address smoking need to bring community, culture together<br>- Strengthening existing community connection and educate communities about the effects of smoking, and health professionals about how to support women to stop smoking.	Connection to Culture, Family and Community
Doyle, J., et al. (2016) (98)	Victoria	2011 and 2014	Prospective study	- Members of the ACCOs and University Health Promotion Alliance	- 88 health promotion activities from 12 different programs across the ACCOs	- There was a wide range of health promotion activities addressing environmental and social determinants of health, as well as physical activity, nutrition, and weight loss.<br>- The most common focus of the activities was social connectedness (76 %).<br>- Physical activity was represented in two thirds of the activities, and nutrition, weight loss and culture were each a focus of about half of the activities.	- First Peoples' health promotion in the region encompasses a broad range of social, cultural, lifestyle and community development activities, including reclaiming and strengthening cultural identity and social connectedness as a response to colonisation	Social connectedness

STable 2. Characteristics and findings of studies reported quality of care and its attributes

Author (year)	Jurisdiction	Duration of the study	Study type/design	Study participants	Study sample (n)	Key findings	Conclusions	WHO good PHC attribute assessed
Bailie, C., et al. (2016) (52)	SA, Queensland, WA, NT	2012 to 2014	Cross – sectional study	- Adult population (aged 15-54) of 101 health for centres to determine adherence to delivery chronic disease preventive services	- 3,623 adult clients	- The delivery of recommended chronic diseases preventive care is good delivery but poor follow-up client after diagnosed with abnormal findings<br>- Client factors (age between 25 and 34 years, female sex, and more regular attendance) small client population and urban centres were associated with higher quality of care	- There is a wide variation in documented preventive care delivery, poor follow-up of abnormal findings, and system factors that influence quality of care	Quality of care
Bailie, J., et al. (2017) (82)	NSW, SA, Queensland, WA, NT, Victoria	2005 to 2014	Mixed-method study	Adult client and clinicians, managers, policy officers, continuous quality improvement (CQI) facilitators and academics	- 17,108 adults with no documented major chronic disease from 137 primary health care (PHC) settings and 367 system assessments	- Priorities to improve preventive Aboriginal PHC include strong Indigenous participation in the PHC service; appropriate team structure and function to support preventive care<br>- Identified priorities to improve aboriginal PHC include following-up abnormal test results; completing cardiovascular risk assessments; family relationships and substance use; providing support for clients identified with emotional wellbeing risk;	- The framework to improve aboriginal PHC may be useful for similar purposes in other parts of the world, with appropriate attention to context in different locations	Quality of care
Bassilios, B., et al. (2017) (26)	National	2013	Qualitative study	Medical Locals from states and Territories	- 18 Medicare Local regions and staffs including<br>- 20 Medicare Local service administrator staff,<br>- 6 referring professionals<br>- 8 mental health professionals	- The child mental health service (CMHS) made good progress towards achieving effective implementation and facilitated the delivery of a high-quality service	- The CMHS established inter-agency linkages (e.g., devoting a professional role— Coordination and Liaison—to this purpose)	Quality of care Coordination (inter-agency linkage)
Larkins, S., et al. (2016) (124)	Queensland, Northern Territory, New South Wales, Western Australia, South Australia	2005 to 2013	Longitudinal clinical audit	- PHC services participating in the Audit and Best Practice for chronic Disease National Research Partnership (ABCD NRP)	73 PHC services providers	- Trends in quality of care varied widely between health services<br>- There were no consistent associations of population size, remoteness, governance model, or accreditation status with positive or negative trends in quality of care.	- The trends in quality of care as reflected by continuous quality improvements (CQI) was not correlated to easily measurable health service characteristics.	Quality of care
Percival, N., et al. (2016) (112)	Northern Territory	January 2008 to December 2010	Interventional study	- PHC centres located in regional and remote Aboriginal communities	4 Indigenous PHC centres (quality improvement activities were measured across 3 years)	- The health promotion continuous quality improvement (CQI) intervention improves staffs' understanding of health promotion.<br>- The CQI intervention improves best practices of health promotion such as engagement and intersectoral partnerships. However, CQI is predominately affected by the emphasis on the clinical and curative services at the PHC services	- CQI can improve the delivery of evidence-based health promotion by engaging front line health practitioners in decision-making processes about the design/redesign of health centre systems to support the delivery of best practice health promotion	Quality of care
Sawyer, M. G., et al. (2013) (113)	South Australia	March 2008 to September 2009	Interventional study	- Socially disadvantaged mothers in South Australia	- 487 mothers (318 home-visiting group – intervention) and 169 mothers in the comparison group)	- Postnatal home-visiting by nurses in a community child health service improved the mothers' perceptions of mother–child relationships and satisfaction with their parental role.	- Home-visiting programmes delivered by community health nurses as part of routine clinical practice have the potential to improve maternal–child	Quality of care (Effectiveness of nurse home-visit)

						- However, postnatal home-visiting by nurses did not improve maternal mental health or reduce prevalence of maternal health-risk behaviours.	relationships and help mothers adjust to their role as parents.	
Jones, M. P., et al. (2021) (105)	Northern Territory	2011–2015	Retrospective study	- Patients, remote area nurses (RANs), aboriginal health practitioners (AHPs) and short- term staff of from 48 emote PHC clinics	- 25 413 patients	- The proportion of resident Aboriginal clients receiving high- quality care varied considerably across indicators and clinics.<br>- There was high quality of care for chronic diseases management such as diabetes was high.<br>- The quality of general/preventive adult health care was low.	- There is no adequate evidence to support the negative effects of increased turnover, decreased stability, and increased reliance on temporary staff on quality of care but with substantial difference in clinic- specific quality factors	Quality of care (Effects of turnover and stability of health staff on quality of care)
Bartlem, K. M., et al. (2016) (117)	New South Wales	2011 to 2014	Non – randomized controlled trial	Clients who attended a face-to-face individual clinical appointment to receive preventive care	- 1787 (n = 805 at baseline, n = 982 at follow-up)	- There was an increase in assessment and provision of preventive care for four chronic disease risk behaviours in community mental health services	- The provision of chronic disease preventive care at community mental health services had a limited effect on four chronic disease risk behaviours	Effectiveness
Wiggers, J et al. (2017) (129)	New South Wales	October 2009 to May 2014	Three-group stepped-wedge trial	- Clients from 56 geographically and administratively defined PHC facilities	5369 clients from 56 community based PHC	- A 12- month sequential intervention at community health care settings shows that provision of both risk assessment and brief advice for all risks is increased to reduce client chronic disease risk behaviours (smoking, healthy diet, and alcohol consumption)<br>- However, no significant increases in care delivery were observed for referral for any risk behaviour, or for physical inactivity.	- The intervention was effective in enhancing assessment of clients' risk status for elements of care that could reduce client's chronic diseases risk	Effectiveness of an implementation intervention
Baum, F., et al. (2014) (130)	South Australia	June 2009 to June 2010	Qualitative study	- Managers, practitioners, administrative staff, and regional health executive staff from the state health department	68 interviews (7 – 15 semi-structured interviews from five PHC services)	- PHC in Australia plays an important role in disease prevention, but there is considerable scope to increase the amount of community-based health promotion<br>- Health reform and consequent reorganizations were seen to reduce the ability of some services to undertake health promotion.	- PHC sites undertakes significant amounts of disease prevention work and a more limited range of health promotion activity	Effectiveness of comprehensive PHC
Brown, S. J., et al. (2019) (123)	South Australia	July 2011 to June 2013	Population-based study	- Reproductive age women living in urban, regional, and remote areas	344 women	- 51% of aboriginal women who used perinatal care perceived that they had experienced discrimination or unfair treatment<br>- Aboriginal women who perceived that they had experienced discrimination in perinatal care were more likely to have a baby with a low birthweight or small for gestational age	- Aboriginal women most at risk of poor infant health outcomes were the least likely to perceive that they received care well matched to their needs.<br>- This implies that culturally sensitive and safe perinatal health care is an urgent priority.	Discrimination or unfair treatment
Forster, D. A., et al. (2016) (115)	Victoria	Sep 2007 to June 2010	Randomised controlled trial	- Women presented to antenatal, intrapartum, and postpartum care	- 2,314 women (1,156 trial arm and 1,158 standard care)	- Compared with standard care, caseload care was associated with higher overall ratings of satisfaction with antenatal care (OR 3.35; 95 % CI: 2.79, 4.03), intrapartum care (OR 2.14; 95 % CI: 1.78, 2.57), hospital postpartum care (OR 1.56, 95 % CI: 1.32, 1.85) and home-based postpartum care (OR 3.19; 95 % CI: 2.64, 3.85).	- For women at low risk of medical complications, caseload midwifery increases women's satisfaction with antenatal, intrapartum, and postpartum care.	- Continuity of care And satisfactory Person centeredness
Ifediora, C. O. and G. D. Rogers (2018) (60)	National	25 to 31 January 2016	Cross – sectional study	- Patients who used Australian after – hours house – call (AHHC)	- 1228 patients	- Patients who required GP follow-ups were likely to be dissatisfied with aspects of the AHHC care received, while those with no follow-ups were generally satisfied	- Patients who used Australian AHHC services either end up requiring no further follow-up, or do so with their own GPs, with few relying on further AHHC visits for follow-up.<br>- The real reasons for follow-up differences observed by age and	Continuity of care

							gender may need to be explored further to ensure that the AHHC services are better used.	
Khalil, H., and S. Lee (2018) (107)	Victoria	2017	Implementation study	- Health professionals working in general health, community health, diabetes, and chronic diseases management, and allied health programs and trained in medication safety	29 participants from 10 rural sites completed the survey	- Medication safety training has improved clinicians' knowledge,<br>- confidence, behaviour, and utilization positively<br>- Medication safety program across the study organization sites relied on 3 main stages	- The success of the implementation of a collaborative medication safety program within a large organization is dependent on emphasizing a wide culture of patient safety and understanding the medication incident reports within an organization.	Medication safety
Hermansyah, A., et al. (2017) (35)	Australian Capital Territory, New South Wales, Queensland, South Australia, Victoria	December 2014 to August 2015	Qualitative study	- Key stakeholders within and beyond community pharmacy in Eastern half Australia	- 27 Key increments	- Social, economic and policy factors are affecting the current context of community pharmacy at micro, meso and macro levels<br>- Community pharmacy has untapped potential in primary health care, but it has been slow to change to meet opportunities available in the current situation	- As the current situation is complex, interrelated, and dynamic with often unintended and unpredictable consequences, this paper suggests that<br>- The existing complex interrelated, and dynamic situation affects community pharmacy enormously<br>- Therefore, due emphasis should be given to micro, meso and macro levels community pharmacy when policy changes.	Pharmacy practice in PHC

STable 3. Characteristics and findings of studies on patient/person centeredness of PHC and community engagement

Author (year)	Jurisdiction	Duration of the study	Study type/design	Study participants	Study sample (n)	Key findings	Conclusions	WHO good PHC attribute assessed
Havas, K., et al. (2017) (59)	Queensland	March to November 2015	Cross – sectional study	- Persons diagnosed with or self-reported chronic kidney disease (CKD) (any stage) aged ≥18 years old and had a self-	97 persons with self-reported or diagnosed CKD (any stage)	- Clients show strong interest in receiving mental health, physical health, early CKD diagnosis particularly for younger population and women were desired at PHC setting.	- Information about CKD and medications<br>- Prioritising strategies in patient education<br>- Varying levels of engagement to person-centred approach at PHC setting are required	Person – centred
Harrison, R., et al. (2019) (33)	New South Wales	2019	Qualitative study	- Healthcare professionals employed across four community healthcare	21 healthcare professionals (19 females and 2 males)	- Adopting patient-centre approach and recognition of diversity within communities and individuals in those communities, all with their own story, was described as pivotal to effective engagement	- Healthcare providers' cultural competence and effective consumer engagement are closely linked in ethnic minority populations.<br>- Embedding cultural competence as a health system, service and professional capability is therefore critical to ensure equitable healthcare quality for consumers from all ethnic backgrounds.	Patient- centred
John, J. R., et al. (2020) (108)	New south Wales	December 2016 to October 2017	Interventional study	- Patients in the chronic disease management program(wellNet)and comparison population from closest GP	3,401 (352 patients and 3,049 comparison group)	- Patients in the WellNet chronic disease management program showed significant reduction in systolic blood pressure.	- Patients in WellNet program experienced statistically significant and clinically meaningful improvement in BP during the follow-up.	Patient-centred
John, J. R., et al. (2020) (109)	New South Wales	December 2016 to October 2017	Interventional study	- Primary care patients enrolled in six GPs in Northern Sydney	- 636 patients conveniently selected	- Older chronic disease patients and those with private insurance patients were significantly associated with lower patient activation model (PMA) scores showing that they are less likely to get patient centred care.<br>- However, those who had higher baseline patient activation model (PAM) score were more likely to receive with higher patient centred follow-up for their chronic conditions at the primary care	- Patient-Centred Medical Home (PCMH) has the potential to improve patient engagement which can lead to long-term health benefits and sustained self-management behaviours.	Person centeredness (patient-centred)
John, J. R., et al. (2021) (110)	New south Wales	December 2016 to October 2017	Interventional study	- Patients in the chronic disease management program (WellNet)and comparison population from closest GP	1851 participants (163 WellNet group and 1,688 comparison group)	- WellNet patients saw a larger increase in the percentage of patients achieving glycaemic control<br>- Patients withdrawn from WellNet were associated with poor glycaemic control at follow-up	- Patient-centred medical home (PCMH) model is effective in primary care.<br>- PCMH model of GP care is effective in improving diabetes and other clinical outcomes among patients with T2D	Patient-centred
John, J. R., et al. (2021) (111)	New South Wales	December 2016 to October 2017	Interventional study	- Selected patients aged > 40 years with one or more chronic conditions	616 eligible from 6 general practices	- There was statistically significant difference in mean quality of life scores after intervention with a change of 0.03 (95% CI: 0.01, 0.05).<br>- There were significant improvements in the pain and symptom scores in both after the implementation of person-centred PHC	- Person-centred care at PHC improved health-related quality of life of patients with one or more chronic condition	Patient-centred
Khanam, M. A., et al. (2019) (97)	National	January 2013 to June 2016	Cohort study	Patients age ≥ 18 year with chronic kidney diseases (CKD) diagnosed by their GP based on pathology results	19,712 eligible patients from 329 general practices	- Complete monitoring was performed for 25% of the cohort; 54.9% among patients with concomitant diabetes and 14.1% among patients without diabetes. Patients with diabetes, hypertension and a documented diagnosis of	- Patients with CKD who also had diabetes were better monitored, compared with patients without diabetes.	Patient monitoring

						CKD were more likely to have complete monitoring.		
Odgers-Jewell, K., et al. (2017) (120)	Northern Territory	2008	Evaluation study	- Patients with diagnosed type 2 diabetes mellitus	- 13 participants	- Patient – centred, patient – directed, group-based health promotion intervention for the management of type 2 diabetes mellitus is associated with a significant mean difference in patients' weight, body mass index and waist circumference.	- Patient – centred, patient – directed, group-based intervention for the management of type 2 diabetes mellitus was both feasible and acceptable to patients	Patient – centred
Schmidt, B., et al. (2016) (47)	Queensland	June to 30 August 2013	Qualitative study	Indigenous Health Workers (IHWs) with a Certificate IV in Aboriginal and Torres Strait Islander PHC to deliver chronic disease management for a caseload of clients.	- 377 project records of health worker activity	- Service management, training, client engagement, clarification of indigenous health workers (IHWs) role and infrastructure were factor affecting the effectiveness of PHC model in remote indigenous care coordination	- Placing skilled and dedicated IHWs to improve care coordination is insufficient to improve chronic disease outcomes.	Client-centred

STable 4. Characteristics of studies assessed coordination and/or multi-stakeholder collaboration of PHC services

Author (year)	Jurisdiction	Duration of the study	Study type/design	Study participants	Study sample (n)	Key findings	Conclusions	WHO good PHC attribute assessed
Isaacs, A., et al. (2019) (126)	Victoria	2019	Longitudinal study	- Persons with severe and persistent mental illness	- 337 clients had both baseline and follow-up data	- At baseline most frequently reported unmet needs were psychological distress, daytime activity, and company (89%, 72%, and 67%, respectively).<br>- At follow-up, these had decreased to 27%, 22%, and 22%, respectively.<br>- Meeting accommodation need for people with severe mental illness is associated with changes in monetary needs (adjusted OR 2.87, 95% CI 1.76, 4.69) other needs such as childcare, food, safety to self, education, and access to other services	- Reducing needs of persons with SPMI is the starting point of recovery and is a good indicator of psychiatric care.<br>- Care coordination is a useful way to address multiple and complex needs of persons with SPMI. While addressing needs, priority must be given to meeting accommodation needs.	Coordination
Kay, M., et al. (2016) (37)	Queensland	2013	Qualitative study	- Refugee health leaders, pharmacists, practice nurses, and GPs	12 people (2 GPs, 3 registered nurses [and 4 pharmacists) and 3 refugee health leaders	Communication and language barriers, limited health literacy and financial cost were five barriers and better coordination between healthcare providers and improved healthcare provider training were facilitators of quality use of medicines in refuges	- This study offers new understandings towards enhancing quality use of medicines in refugee communities and practical insights to assist the targeting of resources for future interventions.	Coordination
Hulme Chambers, A., et al. (2018) (119)	Victoria	2010 to 2011	Evaluation study	- Community members and service managers involved with Smart and Deadly (Smart and Deadly: a community participation approach to sexual health promotion)	- 32 participants (5 focus groups 13 interviews	- Trust is the foundation community participation and cultural safety and cultural literacy, community control, and legacy and sustainability were an important facilitator of community participation in Aboriginal youths' sexual health.	- YARN (community discussion) is highly productive in identifying facilitators of community participation with Aboriginal youths' sexual health	Community participation
Reeve, C., et al. (2015)) **(76)**	Western Australia	2006 – 2012	Mixed method	- 5 stake-holder interviews<br>- 4 focus groups comprised<br>- 10 health service providers<br>- 14 health governing council and<br>- 6 community members	Selected stake holders, health providers and community members	- Community participation in health care reform can lead to innovative PHC delivery<br>- Community-led partnership provides sustainable health service in PHC setting<br>- Primary health care clinics, clinical sessions and PHC programs increased in number and diversity<br>- Total GPs, allied health professionals and number of PHC follow-up appointments were increased over years	- This formal community participation in health service reform is essential to meet the major challenges of providing PHC in a very remote and socially disadvantaged area.	Coordination (community participation) and accessibility of PHC)
Baum, F. E., et al. (2013) (24)	South Australia and Northern Territory	2010	Qualitative study	- 6 PHC services including Aboriginal Controlled PHCs	68 interviews from selected 6 PHC services	- Although the PHC undertake policy advocacy towards social determinant of health (SDH) that create healthier communities, there are barriers to the system.<br>- Primary health care workers are required to transverse "dilemmatic space" in providing advocacy services vs the actual PHC work.	- The PHC services need to be more responsive to social determinants and should support and building alliances with communities and social movements.	Coordination (Multi-disciplinary)
Robinson, S., et al. (2015) (87)	Queensland, Victoria, Western Australia, New South Wales, South Australia, Tasmania,	2013	National survey	- National survey of Medicare Locals	61 Medicare Local Chief Executive Officers (CEOs) from Six states	- There are considerable differences in the form, function, and implementations of the Medicare Local organisations<br>- This diversity and lack of guidance from government impacted on the overall success of the reform. Other barriers to reform	- Findings from this study produce important insights for primary care reform in Australia; and internationally it adds to the growing body of knowledge around primary care reform.	Coordination (stakeholder engagement)

						included difficulties in stakeholder relationships and limited incentives (financial and other) to drive and influence change.		
Lloyd, JE., et al (2015) (40)	New South Wales	Sept 2012 to Feb 2013	Qualitative study	- Aboriginal people who had been in prison and their families and community service providers.	- 30 interviews with purposively selected prisoners and families<br>- 12 interviews with Aboriginals who were in prison<br>- 10 interviews with family members<br>- 8 interviews community service providers	- Effective PHC on release and during transition to inmates show positive effect<br>- Poor communication/link between PHC providers prior to an inmate's release, contributed to a lack of comprehensive management of chronic conditions.<br>- System level barriers such as timely communication between in-custody and community providers significantly affects health outcomes of people released from prison	- For Aboriginal former inmates and family members, release from prison was a period of significant emotional stress<br>- To support their transition into the community, Aboriginal former inmates would benefit from immediate access to culturally-responsive PHC<br>- However, pre-release planning is not always available, especially for Aboriginal inmates who are more likely to be on remand or in custody for less than six months.	Coordination/Link between Prisons and PHC
Grace, S., et al. (2018) (31)	New South Wales	2018	Qualitative study	- Young adults, middle-aged adults, and seniors	13 people participated in the three focus groups.	- The three themes emerged from the qualitative finding and the health service has non-judgmental communication with healthcare providers. Health care consumers nee flexibility in healthcare services.	- Although the health care consumers integrate their own health care by researching practitioners and services, integration without the support of healthcare providers, healthcare integration, knowledge exchange and risk management are limited	Integration
Ziersch, A., et al. (2020) (77)	National	2012 to 2017	Mixed method	- Staff working in Medical Locals and PHN, Organisation working with migrant and refugees	- Survey<br>- 210 ML and 66 PHN staffs<br>- Interviews<br>- 50 ML and 55 PHN staffs<br>- 62 participants from 8 national refugee organisations	- 48% of MLs and 55% of PHNs did not report any activities on migrant health, and 78% and 62% did not report any activities for refugees, respectively.<br>- Factors associated with whether ML and PHN focus on migrant and refugee health include determination of local priority areas, policy context and funding, collaboration with migrant and refugee organisations and communities, and mechanisms for engagement.	- Despite the importance of primary health care for migrants and refugees, there was relatively little attention paid to these population groups in MLs and PHNs, with a small number of notable exceptions.	Coordination/organisations of migrant and refugee health
Ziersch, A., et al. (2020) (51)	South Australia	2016	Qualitative study	- People from refugee backgrounds resettled in a rural	44 participants	- A sense of safety and some elements of social connectedness and support were key enablers for integration and health and wellbeing, with main challenges including limitations in employment opportunities, mismatched education provision, experiences of discrimination and constrained access to services.	- Challenges experienced by refugees resettled in rural areas can affect integration, health and wellbeing and subsequent onward migration intentions.	Integration of migrant services
Lawn, S et al. 2014 (39)	South Australia	June 2011 to March 2012	Qualitative study	- Staffs from GP plus community health service	250 community healthcare staff from GP plus community health service	- Infrastructural impediments to collaboration; territorialism; and interprofessional practice (IPP) simply not on the agenda.<br>- Dedicated staff and resources are needed to keep IPP on the agenda of health service organisations.<br>- Establishing IPP within newly co-located services is a process that needs time to develop, as part of teams building trust with each other in new circumstances, to	- Co-located health service systems can be complex, with competing priorities and differing strategic plans and performance indicators to meet.<br>- Integration of primary health services: being put together does not mean they will work together	Integration of PHC services

						eventually build a new cultural identity for the co-located services.		
Reeve, C., et al. (2015) (69)	Western Australian	2006 to 2012	Cross – sectional study	- Population in the study area	- Population with health record from 2006–12	- Primary health care services provided to very remote outlying communities has notably increased<br>- Quality of care has been improved with down trending mortality.<br>- Health assessment uptake increased from 13% of the eligible population to 61%, leading to 73% of those identified with diabetes being placed on a care plan	- Strengthening PHC services by addressing key enablers and sustainability requirements can translate into population health gains consistent with the goals underpinning the National Health Care Reform and Closing the Gap policies, and may potentially reduce health inequity for remote-living Aboriginal Australians	Integration of health promotion
Reifels, L., et al. (2018) (46)	National	- November 2013 to January 2014	Qualitative study	- Service providers (primary care agency staff, referrers, and mental health professionals)	- 31 metal health service providers in PHC setting	- Agency-level implementation strategies to enhance access to culturally appropriate mental health service for Indigenous stakeholder	- National efforts to enhance mainstream mental healthcare in PHC services for Indigenous populations are critically dependent on effective local agency- and provider level strategies to optimise the integration, adaptation, and utility of these services within local Indigenous community and healthcare service contexts.	Service integration
Massi, L., et al. (2021) (41)	Northern Territory	- September 2018 to April 2019	Qualitative study	- Clients, staff, and Elders/family members	- 76 participants (26 clients, 47 staff and 3 Elders/family members)	- Confusion around program scope, duplication of care, and tensions over 'ownership' of clients were barriers for service integration.<br>- Knowledge and promotion of the program, cultural safety and case coordination, co-location and partnership forums were identified as existing or potential barries for service integration	- Effective service integration is essential to maximise access and acceptability of the Australian Nurse Family Partnership Program (ANFPP)	Service integration (Interagency)
Tooher, R., et al. (2017) (49)	South Australia	November 2010 to June 2011	Qualitative study	- People working in school-based health programmes	19 participants	- Successful intersectoral collaboration provided the necessary to understand the intersectoral point-of-view and collaboration between complex systems was a skilled endeavour which relied on a strong foundation of communication and interpersonal professional relationships	- Aligning divergent sectoral agendas early in the collaborative process was essential for ensuring that all partners could meet their core business needs while also delivering the school-based health promotion programme outcomes.	Intersectoral coordination
Young, C. E., et al. (2020) (118)	Queensland	2016 and June 2017	Process evaluation	- Primary – secondary health services that used GP case conferences (CCs)	four GP–specialist care	- Establishment of integrated care between primary and secondary care providers, leading to the development of key recommendations for routine integration has benefits and challenges	- Primary–secondary integration is conceptually simple and operationally difficult, embedding it into day-to-day practice is possible	Integrated health care
Kildea, S., et al. (2013) (64)	New South Wales	1998 to 2009	Populating based study	- Births from indigenous and non-indigenous population	Total of 45,216 population<br>- 1,523 births to Indigenous mothers and<br>- 43,693 births to non-Indigenous mothers	After adjusting demographic, maternal, and obstetric risk factors, Indigenous women were more likely to experience<br>- Teenage birth rate (aOR 4.24, 95% CI 3.55, 5.07)<br>- Preterm birth (aOR 1.21: 95% CI 1.01, 1.46)<br>- Low birth weight (aOR 1.31, 95% CI 1.09, 1.58)	- There were relatively stable trends of maternal and neonatal outcomes (i.e. small for gestational age, low birth weight babies, and perinatal mortality for both cohorts.<br>- However, there is significant gap in maternal and neonatal outcome between indigenous and non-indigenous which implies that new models of care which recognise the	Multiagency approach

							heterogeneity of Indigenous communities.	
Elnour, A. A., et al. (2015) **(29)**	All Australian states and territories	April 2013 – December 2013	Qualitative study	Frontline staffs including:<br>- 19 GPs<br>- 18 practice managers (PMs)<br>- 15 practices nurses (PNs) and<br>- 1 Community pharmacist	22 high-performing general practices	- There was a perceived design failure of previously implemented primary health organisations (PHOs) before the PHNs come<br>- Most participants perceived that they had loss of support, unaddressed staff needs such as training and overstaffed in some cases<br>- The GP staffs working Medicare Locals (MLs) had more of political role than PHC role	- PHNs will build on the strengths of previous PHOs and create locality structures and processes that maximise the potential for clinical engagement.	Multi-faceted communication strategy
Edmond, K. M., et al. (2018) (57)	National	2012 to 2014	Cross – sectional study	- Families of young Indigenous children	2466 patient client files from 109 remote, rural, and urban PHC	- Families of children aged 3–11 months (39.5%) were more likely to receive social and emotional wellbeing services than families of children aged 12–59 months (30.0%) [aOR] 1.68 95% CI 1.33 to 2.13).<br>- Remote area families (32.6%) received similar services to rural (29.4%) and urban families (44.0%)	- The families of young Indigenous children appear to receive priority for social and emotional wellbeing care in Australian PHC. However, many Indigenous families are not receiving services.<br>- Improvement in resourcing and support of social and emotional wellbeing services in PHC is needed.	- Multi-stakeholder collaboration
Loban, E., et al. (2021) (81)	National and Canada collaboration	August 2016 to September 2018	Mixed Methods Study	- Selected participants form the 5 multi-stakeholder partnerships	- 5 multi-stakeholder PHC partnerships with 54 participants<br>- Service linkage partnership (n = 9)<br>- Community health resource partnership (n = 19)<br>- DM self-management partnership (n = 7)<br>- Primary health connection partnership (n = 12)<br>- Community outreach partnership (n = 8)<br>- 14 partnership meetings and individual semi-structured interviews (n = 16)	- Although it is complex partnership synergise PHC activities and it is associated with partnership leadership, administration and management, decision-making, the ability of partnerships to optimize the involvement of partners and the sufficiency of nonfinancial resources.<br>- The Partnership Synergy framework was useful in assessing the intermediate outcomes of ongoing partnerships	- The partnership is directly related with synergistic performance of PHC services, and it was useful in assessing the intermediate outcomes of ongoing partnerships when it was too early to assess the achievement of long-term intended outcomes	Stakeholder partnership at PHC
Green, A., et al. (2020) (32)	New South Wales	2019	Qualitative study	- The health, education, and social service sectors including governmental, non-governmental and private practice	- 24 organisations from different sectors (13 health,8 education and 3 social service)	- Findings of this study demonstrate the complex interplay of factors related to the cross-sector involvement of providers in early intervention service provision.<br>- Consideration of these factors is required to facilitate collaborative cross-sector responses to improve service access for Aboriginal families	- Collaboration across the health, education, and social service sectors is required to enable service access for Aboriginal children with a disability and their families, although it is evidently complex concept.	Cross-sectoral collaboration
Taylor, K. P., et al. (2013) (48)	West Australia	2012	Qualitative study	- Senior managers, clinical team leaders and counsellors	- 16 staff (5 senior managers, 5 clinical team leaders and 6	- Communication issues, partner unfamiliarity, divergent views regarding staff competencies, staff turnover and different ways of working are challenges for	- Partnerships should have adequate time, budget and be suitably mature and consolidated before the partnership becomes	Partnerships and collaboration

					counsellors) from 4 services	Aboriginal mainstreaming partnership. However, richness and diversity to treatment possibilities and opportunities to explore different, more cultural appropriateness enhancing substance use service enhancement partnership	operational. Partnership and collaborations should have documentations that makes clear and embedded working arrangements between partners to ameliorate many issues.	
Davy, C., et al. (2016) (27)	Central Australia, New South Wales, Queensland	July 2008 to February 2010	Qualitative study	- Aboriginal people with and without chronic healthcare providers, healthcare service managers or administrative staff	- 223 Aboriginal people and health professionals (126 Aboriginal people and<br>- 97 health care providers)	- Aboriginal participants prioritise care provider is both physically and emotionally welcoming, trustworthiness of healthcare providers and strong relationships with patients for when they face acute health issues. These are also important factors for encouraging sustained engagement overtime.	- Responsibility for sustaining relationships does not rest solely with Aboriginal patients. Rather, healthcare providers need to commit to the process of building and maintaining relationships.	Engagement and relationship between PHC
Durey, A., et al. (2016) (28)	West Australia	2012	Qualitative study	- Local Aboriginal community members formed District Aboriginal Health Action Groups (DAHAGs)	60 participants	- Engagement process between Aboriginal community and health services was effective: it was driven and owned by the Aboriginal community, captured a broad range of views, and increased Aboriginal community participation<br>- It built community capacity through regular community forums<br>- Participants reported health services improved in community and hospital settings, leading to increased access and trust in local health services.	- Actively engaging the Aboriginal community in decisions about their health care was a key element in improving local health services, increasing Aboriginal people's trust and access to care.	Engagement between PHC

STable 5. Characteristics of studies on health workforce in PHC settings

Author (year)	Jurisdiction	Duration of the study	Study type/design	Study participants	Study sample (n)	Key findings	Conclusions	WHO good PHC attribute assessed
Bloomfield, J. G., et al. (2015) (91)	National	July to August 2014	Survey	- Final year nursing student	- 456 final nursing students	- Despite 98.2% of students exposed to PHC, only 22.8% show expressed interest to work in PHC after registration.	- Considering the increasing aging population, prevalence of chronic disease with a predicted PHC workforce shortage, education and workplace strategies aimed at attracting new graduates in to PHC are crucial.	Health workforce
Swami, M., and A. Scott (2021) (88)	National	2008-2014	National survey	- General practice services and	- 8,751 observations on 2,058 GPs with complete data	- Rural workforce incentives program to increase access increased the number of GPs in practices in newly eligible areas increased. However, no evidence is found that this reduces waiting times for existing patients	- Financial incentives may only play a limited role in improving access to primary care and should not be the only solution to address medical workforce shortages in underserved areas.	Health work force (incentive)
McMurray, A., et al. (2018) (73)	Queensland	2016	Mixed method	- Chronic diseases patients, general practitioners and practicing nurses participated in integrated care	- 33 randomly selected patients (7 focus group discussion)<br>- 74 health professionals (55 general practitioners and 19 practice nurses)	- Chronic disease patients expressed high satisfaction with the nurse navigators.<br>- General practitioners and practice nurses identified the importance of information, communication, coordination, advocacy, liaison, and patients' positive health outcomes, with 73% of GPs and 84% of PNs reporting being satisfied with the NN role.	- The navigation role is effective and accepted by patients, general practice, and nurse practitioner in enhancing access to care of chronic diseases patients by bridging the gap between primary and secondary care.	Nurse Navigator role in integrated care
Halcomb, E. and C. Ashley (2017) (58)	National	2016	Cross – sectional study	- Nurses working in primary health care settings	- 1166 nurses	- Patient interactions, respect, teamwork, collegiality, and autonomy were identified as the most satisfying professional aspects of being nurse<br>- However, poor financial support and remuneration, lack of a career path, physical work environment and time constraints<br>- National re-structuring of the primary health care was seen as a barrier to role stability and ability to work to a full scope of practice.	- This study has identified a range of positive and negative professional and personal aspects of the primary health care nursing role, which may impact on staff recruitment and retention<br>- The rate of turnover of nurses working in primary health care has been associated with job satisfaction and professional barriers.	Nurses' satisfaction in their PHC work
Hegney, D. G., et al. (2013) (84)	Queensland	2013	Mixed-method study	- Patient who had type 2 diabetes, stable ischaemic heart disease, hypertension and above 18 years<br>- Key stakeholders	- 3 practice managers<br>- 5 participating nurses<br>- 5 participating GPs<br>- 38 male and female patients	- This study showed that nurses provided acceptable, feasible and sustainable chronic disease management<br>- The collaborative involvement of doctors was intrinsic to patient acceptability of nurse-led care that facilitated job satisfaction, and therefore retention and growth within this nursing speciality.	- Nurse led chronic diseases management model in GPs was both feasible and acceptable to the participating practices and should be funded from Practice Nurse Incentive Program (PNIP) for its sustainability	Professional collaboration
Kelehera, H. and R. Parker (2013) (38)	National	August to September 2008	Qualitative study	Registered nurses	- Registered nurses	- Nurses are enthusiastic about being involved in health promotion and valued the work that they do. However, nurses' opportunities are undoubtedly constrained by both the general practice setting and their educational preparation	- Health promotion practices of primary care nurses were most commonly in the downstream realm of disease prevention and health education, but nurses aspired to take on roles in more upstream work of partnerships and collaboration	Perceptions of nurses to their current and potential roles

McFarlane, K., et al. (2017) (66)	Queensland	2015	Cross – sectional study	- Primary health service providers from 11 remote Queensland communities	- 63 staffs including medical, allied health, Aboriginal and Torres Strait Islander health workers and health practitioners and corporate support staff	- Health promotion is delivered across a continuum of one-on-one approaches through to population advocacy and policy change efforts<br>- The attitude towards health promotion was positive<br>- Health promotion capacity can be enhanced at both individual and organisational levels	- Workforce insights have identified areas for continued support and areas that, now identified, can be targeted to strengthen the health promotion capacity of remote Aboriginal community controlled PHC services	Health workforce insights in health promotion
Bentley, M., et al. (2018) (78)	South Australia	2012 to 2013	Mixed method study	- Health practitioners and managers	- 154 participants in the survey and 60 interviews from 6 PHC services	- Interrelated factors affecting interprofessional work at the service, from contextual, organisational process and relational domains.<br>- Funding cuts, and policy changes that saw a reorientation and medicalisation	- Interprofessional teamwork in PHC and Aboriginal community-controlled health service is highly valued by frontline staffs in the service	Interprofessional teamwork
Miller, E. M., et al. (2021) (43)	Victoria	2021	Qualitative study	- Carers providing end-of-life care to a person in their home who had been diagnosed with a life-limiting illness	- 9 carers	- Regional/rural carers have an added burden of travel stress as well as feeling overwhelmed, isolated, and physically and emotionally exhausted. While the engagement of specialist palliative care services is important, education is still needed to build capacity within the primary palliative care workforce, confirming the importance of timely referrals to a specialist palliative care practitioner if pain or symptom control is not effectively managed.	- Providing palliative and end-of-life care in the home is an exhausting and emotionally draining role for unpaid, primary carers. Multiple supports are needed to sustain primary carers, as they play an essential role in the primary health care system.	Role and Lived Experience PHC providers
Reath, J., et al. (2018) (80)	National	2017	Mixed methods	- Cultural educators (CE)/ cultural mentors (CMs), Medical Educators, General Practice Supervisors and Registrars, and representatives of Regional Training Organisations, Indigenous Health Training Posts, and other key organisations.	- 95 interviews representing 13 Registered Training Organisation (RTOs)<br>- 55 valid survey responses<br>- 7 regional training delegates<br>- 10 cultural education/cultural mentors<br>- 2 training posts<br>- 36 Registrars<br>- Interview data were collected from 90 participants representing, 11 Indigenous Health Training Posts and representatives of 18 other stakeholder organisations	- Aboriginal and Torres Strait Islander cultural educators and mentors play important roles in in Australian GP education<br>- Cultural education and cultural mentorship provide baseline learning as preparation for clinical practice and help as a longitudinal, relationship-based learning respectively.<br>- Finding people with skills, employment, support, remuneration of cultural educators and mentor are important challenges described in interview	- Professional and organisational support is needed to ensure delivery of respectful and effective cultural education within general practitioner training.	Roles of cultural educational and cultural mentors
Morell, A. L., et al. (2014) (94)	National	January 2012 to February 2013	Survey	- Health professionals involved in the attraction, recruitment, and distribution of health professionals in rural and remote Australia program	- 349 health professionals	- 87% were recruited to regional areas, with the remaining 13% recruited to remote areas. 60% of recruits were domestically trained. Nurses (29%) and physiotherapists (21%) were the top two professionals to accepted and recruited in rural and remote areas.	- Rural Health Professionals Program is effective to improve the distribution of health professionals to attract and retain health professional in rural locations.	Rural and remote health workforce attraction, recruitment, and distribution

						- While financial support is the most cited reason by recruits (51%) to be recruited in rural and remote areas, being domestically trained, having previously lived in a rural or remote location, being a nurse (as opposed to an allied health professional) and older age were other factors to accept remote recruitment.		
Keane, S., et al. (2013) (92)	New South Wales	2008	Survey	Allied health professionals from 21 eligible allied health professions	1823 respondent (833 from public group) and 756 from private	- Although factor differed between public and private groups, age was the strongest predictor of intention to leave with younger and older groups being significantly more likely to leave than middle aged<br>- High clinical demand, private, professional isolation were associated with intention to leave among health professionals	- In this study there were differences between public versus private sector health professionals in terms of intention to leave and suggests that effectiveness of policy initiatives may be improved through better targeting	Retention of rural allied health workforce
Lam, L., et al. (2018) (86)	New South Wales	July to November 2016	Mixed-methods study	- Health professionals working in aged care settings	109 healthcare workers from 12 residential aged care facilities	- Patient factors such as decreased capacity to make informed decisions were identified as barriers that could be circumvented by pre-emptive implementation of advanced care planning discussion in aged care homes<br>- The rural setting was identified as a facilitator due to a supportive community, which helped to mitigate barriers such as limited staffing	- Advanced care planning plays a critical role in aged care and end-of-life care, and several factors influence its implementation<br>- Attitudes towards advanced care planning in rural New South Wales are highly positive.<br>- The rural setting is a facilitator to advanced care planning in aged care settings.	Attitudes and practices of healthcare workers in advanced care
McCullough, K., et al. (2021) (42)	Northern Territory, Western Australia, Queensland	2014 and 2015	Qualitative study	- Nurse practitioners and registered nurses working in 'remote' and 'very remote'	24 health professionals (13 nurse practitioners and 11 registered nurses)	- Nurses in remote settings considered primary health care to be a holistic social model of care that included caring for the community as well as the individual. However, they were unable to provide care consistent with their intention due to the resource-poor nature of the remote setting. Inadequate physical resources, limited specialist health services, and a lack of time were found to impact on remote area nurses' abilities to provide primary health care.	- Nurses working in remote and very remote PHC services reported that they are unable to provide PHC to their satisfaction due to a lack of staffing and physical resources. This perceived gap is described as a cause of stress and dissatisfaction among remote area nurses in primary health care.	Health care workers challenges to provide PHC

STable 6. Characteristics of studies reported clinical decision making, knowledge translation and evidence-based practice

Author (year)	Jurisdiction	Duration of the study	Study type/design	Study participants	Study sample (n)	Key findings	Conclusions	WHO good PHC attribute assessed
Sinclair, C., et al. (2016) (106)	West Australia	2016	Experimental study	- General practices	- 70 GPs	- Prompt advanced care planning (ACP) is more likely to be received by older patients, with malignant or cardiovascular disease, severe clinical presentations, good doctor-patient relationship, female gender, and poor family support.<br>- Positive GP attitudes toward ACP were associated with greater likelihood of initiating ACP promptly	- Patients with presented with higher mortality risk were identified as needing ACP.<br>- GPs with sensitive attitude to ACP and good doctor-patient interaction were more likely to initiate ACP.	Clinical decision
Harris, M. F., et al. (2017) (114)	New south Wales, Victoria, South	2012/2013	Cluster randomised controlled trial	- Patients (40-69 years) without chronic disease	589 patients	- Preventive evidence to practice education (PEP) intervention was not associated with changes in the proportion of patients reporting a BP,	- This study highlights some of the challenges in providing suitable chronic disease preventive	Evidence-based practice

	Australia, Queensland			who were receipt of GP services and referral		cholesterol, glucose, or weight check in either group.<br>- Less than one in six at-risk patients reported receiving lifestyle advice or referral at baseline with little change at follow up.<br>- There was no significant change in the proportion of patients who reported being at-risk for diet, physical activity (PA) or weight, and no changes in PA, diet, and BMI	interventions which are both scalable to whole practice populations and meet the needs of diverse practice structures.	
Dadich, A., et.al. (2016) (56)	New South Wales	August 2011 to January 2013	Cross – sectional study	- Primary care clinicians – including GPs and Practice Nurses – who were practicing in NSW working in 33 Divisions of General Practice	- 413 primary care clinicians (214 GPs and 217 Practice Nurses)	- The use of clinical aid and online learning in PHC settings improve the perceived impacts the way health professionals practice evidence based clinical practice	- Channels through which evidence-based practices are communicated to healthcare professionals (clinical aid or online learning) shape the ways they engage with, and use, this information.	Evidence-based practices
Dadich, A., et al (2013) (55)	New South Wales	August 2011 to January 2012	Cross – sectional study	- GPs and PNs practicing in NSW	- 431 PHC clinicians (214 GPs and 217 PNs)	- Barriers that hindered resource-use included limited time, limited perceived need, and limited access to, or familiarity with the resources.<br>- The reorganization of the PHC sector and the removal of medical benefits scheme items may have hampered capacity of GPs and NPs to translate evidence-based practices into patient care	- The translation of evidence-based practices into patient care is viable despite PHC reform<br>- The multi-modal approach for knowledge translation potentially valuable in PHC reform<br>- The dissemination of relatively inexpensive resources might influence clinical practices in PHC<br>- PHC reforms to governance and/or funding arrangements may widen the void between evidence-based practices and patient care.	Knowledge translation
Thomas, S., et al. (2019) (121)	New South Wales	June and July 2018	Evaluation study	- Members of the working party (WP) and GPs and practice nurses from practices	- 24 participants took part in 3 focus groups and 4 interviews	- Designing and adopting new ways of working is time consuming and requires new skills, thinking, clarity; who's doing what, when and how?<br>- Genuine engagement and interaction across fields and institutions help build capacity and strengthen motivations	- This process evaluation identified barriers and facilitators to translating research findings into interventions to increase vaccination in a disadvantaged community and suggests<br>- There was strong motivation and opportunity for change, but a need to enhance service capability	Evidence translation (evaluation)
Jayasinghe, U. W., et al. (2016) (62)	New South Wales, Victoria, South Australia, and Queensland	2012 to 2013	Cross – sectional study	- Subsample of people with vascular disease and diabetes factors	739 patients from 30 GPs across four Australian states	- Low health literacy patients were more likely to show higher rates lifestyle risk factors such as smoking, insufficient physical activity, overweight and have lower physical health and lower mental health<br>- Less well-educated, unemployed, and smoking patients with low health literacy reported worse physical health.<br>- Health literacy accounted for 45 and 70% of the total between patient variance in lifestyle risk factors on health-related quality of life	- Addressing health literacy related barriers to preventive care may help reduce some of the disparities in HRQoL.<br>- Recognising and tailoring health related communication to those with low health literacy may improve health outcomes including HRQoL in GP service	Evidence to improve health-related quality of life

STable 7. Characteristics of studies reported PHC service use and recipient of care

Author (year)	Jurisdiction	Duration of the study	Study type/design	Study participants	Study sample (n)	Key findings	Conclusions	WHO good PHC attribute assessed
Perkins, D., et al. (2013) (68)	New South Wales	2007 to 2009	Cross – sectional study	- Health service use for mental health problems	- 2150 participants	- The overall health service use rate for mental health problems during the previous 12 months was better than the national rate (17% vs 11.9%) in this rural population<br>- Remoteness was associated with lower utilisation of non-professional support	- Self-reported patterns of professional and non-professional service use for mental health problems in a rural community cohort revealed relatively higher rates of GP attendance compared with data from metropolitan centres.	Service use
Yang, B. and R. Messom (2021) (70)	New South Wales	2013 to 2015	Cross – sectional study	- General practice and PHC users	- 6, 221, 762 patients	- 41% of emergency department presentations were potential primary care ED presentations over the 5 years<br>- Population rates of potential primary care ED presentations and GP care both increased over the period, with higher potential primary care ED presentation rates in regional areas, and higher GP care rates in major cities.<br>- GP care utilisation was associated with reduced odds for potential primary care ED presentations for patients with five or more GP care services compared with patients with none.<br>- Increased comorbidity index was also associated with increased risk of potential primary care ED presentations.	- General practice (GP) care utilisation was associated with reduced risk for any potential primary care (PPC) ED presentations after adjusting for comorbidity index and other factors.	Health service us/visit
Ryan, S. M., et al. (2014) (125)	Victoria	2004 to 2005	Longitudinal interventional Study	- Parents who have adolescent child	636 parents	- Parental overcontrol was associated with reduced likelihood of service use at follow-up.<br>- No association was found between service use at follow-up and parent gender, socioeconomic status, number of siblings, parent psychopathology, family social connectedness, and prior service use by the parent	- The results indicate that families provide a potential target for interventions aimed at increasing use of professional services for adolescent mental health problems	Service use
Valery, P. C., et al. (2020) (103)	Queensland	2010–2016	Retrospective PHC audit	- Queensland Health-operated PHC services and Aboriginal community-controlled health services (ACCHSs))	- 10 PHC or ACCHSs units serving Indigenous people selected purposively and conveniently<br>- 138 active cancer patients	- Patients PHC service visit increased by 5.95 times on average per year for 12 months following the cancer diagnosis.<br>- Frequency of visits were relatively high in remote areas (IRR= 1.87, 95%CI 1.61–2.17) and among socioeconomic disadvantaged patients IRR= 1.79, 95%CI 1.45–2.21)	- The reliance on PHC services, particularly by patients in remote and disadvantaged communities, has important implications for appropriate resourcing and support for services in these locations.	Health service us/visit
Kruske, S., et al. (2016) (64)	National	August to October 2014	Cross – sectional study	- Primary Maternity Units (PMU) in rural and remote Australia	17 PMUs were identified in rural and remote areas of Australia	- The average distance of the PMUs from referral service was 56 km or 49 minutes and provided care to an average of 59 birthing women per year.<br>- Periodic closures or downgrading of services was common. Low-risk eligibility criteria were universally used, but with some variability.<br>- In most PMUs midwives worked shift work involving both nursing and midwifery duties, with minimal uptake of recent midwifery workforce innovations.	- Only a small number of Primary Maternity Units operate in rural Australia.<br>- Midwifery models of care are under-utilised.<br>- Reliance on medical models impedes service sustainability in rural and remote areas.	Service provision assessment
Joshi, C., et al. (2014) (63)	New South Wales, Queensland,	2012	Cross – sectional study	- 30 general practices	739 patients from the selected 30 general practices	- Insufficient health literacy is associated increased use of preventive PHC such as diet, physical activity or weight management, and referral to	- Patients with insufficient health literacy were more likely to get preventive PHC services from GPs. These findings are	Recipient of care

	Victoria, and South Australia					and attendance at lifestyle modification programs.	positive in that the GPs identify patients with low health literacy and do appropriate referral	
Hussain, J., et al. (2014) (104)	Northern Territory	July 2008 to June 2010	Retrospective study	- Remote central Australian community with Primary health services	- 20 primary healthcare centres (from more than 50 existing remote Central Australian communities)	- There were positive correlations between acute medical evacuations and numbers of face-to-face consultations, remote area nurse consultations and GP practice	- More provision of primary healthcare services is associated with more acute medical evacuations and more remote telephone consultations	Service provision assessment
Zander, K. K., et al. (2016) (89)	Northern Territory	2011	Census	Indigenous communities'	15 Indigenous communities	- Individual characteristics (gender and age) had high impacts on individuals being at home or away on census night and that good health care provision, government subsidised community jobs and<br>- Those living in communities that had recently received new houses were less likely to be away on census night. The results can contribute to the efficiency of service provision and to understanding	- Aboriginal community with jobs in health care were more likely to have temporary mobility. Demographic characteristics explain some temporary mobility.<br>- The propensity to be away on census night is higher for women and people in their teens while babies and older women are more likely to be at home on census night.	Impacts of Service and Infrastructure Provision

Reference

1. Sacks E, Schleiff M, Were M, Chowdhury AM, Perry HB. Communities, universal health coverage and primary health care. Bull World Health Organ. 2020;98(11):773-80.
2. World Health Organization (WHO). Primary health care on the road to universal health coverage: 2019 global monitoring report. 2021. Report No.: 9240004270.
3. Schwarz D, Hirschhorn LR, Kim J-H, Ratcliffe HL, Bitton A. Continuity in primary care: a critical but neglected component for achieving high-quality universal health coverage. BMJ Global Health. 2019;4(3):e001435.
4. Sanders D, Nandi S, Labonté R, Vance C, Van Damme W. From primary health care to universal health coverage—one step forward and two steps back. The Lancet. 2019;394(10199):619-21.
5. Assefa Y, Tesfaye D, Van Damme W, Hill PS. Effectiveness and sustainability of a diagonal investment approach to strengthen the primary health-care system in Ethiopia. The Lancet. 2018;392(10156):1473-81.
6. Binagwaho A, Ghebreyesus TA. Primary healthcare is cornerstone of universal health coverage. BMJ : British Medical Journal (Online). 2019;365.
7. Gargano G. The Bottom-Up Development Model as a Governance Instrument for the Rural Areas. The Cases of Four Local Action Groups (LAGs) in the United Kingdom and in Italy. Sustainability. 2021;13(16):9123.
8. McDonald J, Cumming J, Harris M, Powell Davies G, Burns P. Systematic review of comprehensive primary health care models. 2017.
9. Organization WH. Primary health care in the Western Pacific Region: looking back and future directions. 2018.
10. Fisher M, Freeman T, Mackean T, Friel S, Baum F. Universal health coverage for non-communicable diseases and health equity: lessons from Australian primary healthcare. International Journal of Health Policy and Management. 2022;11(5):690-700.
11. Simpson SJ, Saint V, Bozorgmehr K. Changing the Discourse in Ambitions Towards Universal Health Coverage: Lessons From Australian Primary Healthcare Comment on "Universal Health Coverage for Non-communicable Diseases and Health Equity: Lessons From Australian Primary Healthcare". Int J Health Policy Manag. 2022;11(6):851-4.
12. Australian Government Department of Health. Australia's health landscape infographic. 2017.
13. Australian Institute of Health Welfare. Primary health care. Canberra: AIHW; 2020.
14. Australian Institute of Health Welfare. Australia's health 2016. Canberra: AIHW; 2016.
15. Australian Institute of Health Welfare. Primary health care in Australia. Canberra: AIHW; 2016.
16. Gracey M. Why closing the Aboriginal health gap is so elusive. Internal Medicine Journal. 2014;44(11):1141-3.
17. Freeman T, Baum F, Lawless A, Javanparast S, Jolley G, Labonté R, et al. Revisiting the ability of Australian primary healthcare services to respond to health inequity. Australian journal of primary health. 2016;22(4):332-8.
18. Bailie J, Potts BA, Laycock AF, Abimbola S, Bailie RS, Cunningham FC, et al. Collaboration and knowledge generation in an 18-year quality improvement research programme in Australian Indigenous primary healthcare: a coauthorship network analysis. BMJ open. 2021;11(5):e045101.
19. World Health O. Monitoring the building blocks of health systems: a handbook of indicators and their measurement strategies. Geneva: World Health Organization; 2010 2010.
20. Brown L, Carne A, Bywood P, McIntyre E, Damarell R, Lawrence M, et al. Facilitating access to evidence: Primary Health Care Search Filter. Health Information & Libraries Journal. 2014;31(4):293-302.
21. Health SCo. National primary health care strategic framework. 2013.

22. Dijkmans-Hadley B, Bonney A, Barnett SR. Development of an Australian practice-based research network as a community of practice. Australian journal of primary health. 2015;21(4):373-8.
23. Moher D, Liberati A, Tetzlaff J, Altman DG. Preferred reporting items for systematic reviews and meta-analyses: the PRISMA statement. PLoS Med. 2009;6(7):e1000097.
24. Baum FE, Legge DG, Freeman T, Lawless A, Labonté R, Jolley GM. The potential for multi-disciplinary primary health care services to take action on the social determinants of health: actions and constraints. BMC public health. 2013;13:460.
25. Pearson O, Schwartzkopff K, Dawson A, Hagger C, Karagi A, Davy C, et al. Aboriginal community controlled health organisations address health equity through action on the social determinants of health of Aboriginal and Torres Strait Islander peoples in Australia. BMC public health. 2020;20(1):1859.
26. Bassilios B, Nicholas A, Ftanou M, Fletcher J, Reifels L, King K, et al. Implementing a Primary Mental Health Service for Children: Administrator and Provider Perspectives. Journal of Child and Family Studies. 2017;26(2):497-510.
27. Davy C, Cass A, Brady J, DeVries J, Fewquandie B, Ingram S, et al. Facilitating engagement through strong relationships between primary healthcare and Aboriginal and Torres Strait Islander peoples. Australian and New Zealand journal of public health. 2016;40(6):535-41.
28. Durey A, McEvoy S, Swift-Otero V, Taylor K, Katzenellenbogen J, Bessarab D. Improving healthcare for Aboriginal Australians through effective engagement between community and health services. BMC health services research. 2016;16:224.
29. Elnour AA, Dunbar J, Ford D, Dawda P. General practices' perspectives on medicare locals' performance are critical lessons for the success of primary health networks. Australasian Medical Journal. 2015;8(10):320-4.
30. Freeman T, Edwards T, Baum F, Lawless A, Jolley G, Javanparast S, et al. Cultural respect strategies in Australian Aboriginal primary health care services: beyond education and training of practitioners. Australian and New Zealand journal of public health. 2014;38(4):355-61.
31. Grace S, Bradbury J, Avila C, Du Chesne A. 'The healthcare system is not designed around my needs': How healthcare consumers self-integrate conventional and complementary healthcare services. Complementary therapies in clinical practice. 2018;32:151-6.
32. Green A, Abbott P, Luckett T, Davidson PM, Delaney J, Delaney P, et al. Collaborating across sectors to provide early intervention for Aboriginal and Torres Strait Islander children with disability and their families: a qualitative study of provider perspectives. Journal of interprofessional care. 2020;34(3):388-99.
33. Harrison R, Walton M, Chauhan A, Manias E, Chitkara U, Latanik M, et al. What is the role of cultural competence in ethnic minority consumer engagement? An analysis in community healthcare. International journal for equity in health. 2019;18(1):191.
34. Henderson J, Javanparast S, MacKean T, Freeman T, Baum F, Ziersch A. Commissioning and equity in primary care in Australia: Views from Primary Health Networks. Health & social care in the community. 2018;26(1):80-9.
35. Hermansyah A, Sainsbury E, Krass I. Investigating influences on current community pharmacy practice at micro, meso, and macro levels. Research in social & administrative pharmacy : RSAP. 2017;13(4):727-37.
36. James S, Toombs M, Brodribb W. Barriers and enablers to postpartum contraception among Aboriginal Australian women: Factors influencing contraceptive decisions. Australian Journal of Primary Health. 2018;24(3):241-7.
37. Kay M, Wijayanayaka S, Cook H, Hollingworth S. Understanding quality use of medicines in refugee communities in Australian primary care: a qualitative study. The British journal of general practice : the journal of the Royal College of General Practitioners. 2016;66(647):e397-409.
38. Kelehera H, Parker R. Health promotion by primary care nurses in Australian general practice. Collegian. 2013;20(4):215-21.

39. Lawn S, Lloyd A, King A, Sweet L, Gum L. Integration of primary health services: being put together does not mean they will work together. BMC research notes. 2014;7:66.
40. Lloyd JE, Delaney-Thiele D, Abbott P, Baldry E, McEntyre E, Reath J, et al. The role of primary health care services to better meet the needs of Aboriginal Australians transitioning from prison to the community. BMC family practice. 2015;16:86.
41. Massi L, Hickey S, Maidment SJ, Roe Y, Kildea S, Nelson C, et al. Improving interagency service integration of the Australian Nurse Family Partnership Program for First Nations women and babies: a qualitative study. International Journal for Equity in Health. 2021;20(1).
42. McCullough K, Bayes S, Whitehead L, Williams A, Cope V. We say we are doing primary health care but we're not: Remote area nurses' perspectives on the challenges of providing primary health care services. Collegian. 2021;28(5):534-40.
43. Miller EM, Porter JE, Peel R. Palliative and End-of-Life Care in the Home in Regional/Rural Victoria, Australia: The Role and Lived Experience of Primary Carers. Sage Open Nursing. 2021;7.
44. Parker R, Forrest L, Ward N, McCracken J, Cox D, Derrett J. How acceptable are primary health care nurse practitioners to Australian consumers? Collegian. 2013;20(1):35-41.
45. Raymundo G, Smith-Merry J, McNab J. Experiences of health service literacy and access amongst Australian young adults from migrant backgrounds. Health promotion journal of Australia : official journal of Australian Association of Health Promotion Professionals. 2021;32 Suppl 1:69-79.
46. Reifels L, Nicholas A, Fletcher J, Bassilios B, King K, Ewen S, et al. Enhanced primary mental healthcare for Indigenous Australians: service implementation strategies and perspectives of providers. Global Health Research and Policy. 2018;3(1).
47. Schmidt B, Campbell S, McDermott R. Community health workers as chronic care coordinators: evaluation of an Australian Indigenous primary health care program. Australian and New Zealand journal of public health. 2016;40 Suppl 1:S107-14.
48. Taylor KP, Bessarab D, Hunter L, Thompson SC. Aboriginal-mainstream partnerships: exploring the challenges and enhancers of a collaborative service arrangement for Aboriginal clients with substance use issues. BMC health services research. 2013;13:12.
49. Tooher R, Collins J, Braunack-Mayer A, Burgess T, Skinner SR, O'Keefe M, et al. Intersectoral collaboration to implement school-based health programmes: Australian perspectives. Health Promotion International. 2017;32(2):312-21.
50. Wyndow P, Clifton E, Walker R. Improving Aboriginal Maternal Health by Strengthening Connection to Culture, Family and Community. International Journal of Environmental Research and Public Health. 2020;17(24).
51. Ziersch A, Miller E, Baak M, Mwanri L. Integration and social determinants of health and wellbeing for people from refugee backgrounds resettled in a rural town in South Australia: a qualitative study. BMC public health. 2020;20(1).
52. Bailie C, Matthews V, Bailie J, Burgess P, Copley K, Kennedy C, et al. Determinants and Gaps in Preventive Care Delivery for Indigenous Australians: A Cross-Sectional Analysis. Frontiers in Public Health. 2016;4.
53. Bartlem K, Bowman J, Freund M, Wye P, Lecathelinais C, McElwaine K, et al. Acceptability and Receipt of Preventive Care for Chronic-Disease Health Risk Behaviors Reported by Clients of Community Mental Health Services. Psychiatric services (Washington, DC). 2015;66(8):857-64.
54. Brodribb WE, Mitchell BL, van Driel ML. Practice related factors that may impact on postpartum care for mothers and infants in Australian general practice: a cross-ectional survey. Bmc Health Services Research. 2016;16.
55. Dadich A, Hosseinzadeh H. Healthcare reform: implications for knowledge translation in primary care. BMC health services research. 2013;13:490.
56. Dadich A, Hosseinzadeh H. Communication channels to promote evidence-based practice: a survey of primary care clinicians to determine perceived effects. Health research policy and systems. 2016;14(1):62.

57. Edmond KM, McAuley K, McAullay D, Matthews V, Strobel N, Marriott R, et al. Quality of social and emotional wellbeing services for families of young Indigenous children attending primary care centers; a cross sectional analysis. BMC health services research. 2018;18(1):100.
58. Halcomb E, Ashley C. Australian primary health care nurses most and least satisfying aspects of work. Journal of clinical nursing. 2017;26(3-4):535-45.
59. Havas K, Douglas C, Bonner A. Person-centred care in chronic kidney disease: a cross-sectional study of patients' desires for self-management support. BMC nephrology. 2017;18(1):17.
60. Ifediora CO, Rogers GD. Continuity of care in after-hours house call medical services: An exploration of follow-up patterns in an Australian context. Journal of evaluation in clinical practice. 2018;24(3):514-20.
61. Inder KJ, Handley TE, Fitzgerald M, Lewin TJ, Coleman C, Perkins D, et al. Individual and district-level predictors of alcohol use: cross sectional findings from a rural mental health survey in Australia. BMC public health. 2012;12(1):586.
62. Jayasinghe UW, Harris MF, Parker SM, Litt J, van Driel M, Mazza D, et al. The impact of health literacy and life style risk factors on health-related quality of life of Australian patients. Health and Quality of Life Outcomes. 2016;14.
63. Joshi C, Jayasinghe UW, Parker S, Del Mar C, Russell G, Lloyd J, et al. Does health literacy affect patients' receipt of preventative primary care? A multilevel analysis. BMC family practice. 2014;15:171.
64. Kildea S, Stapleton H, Murphy R, Kosiak M, Gibbons K. The maternal and neonatal outcomes for an urban Indigenous population compared with their non-Indigenous counterparts and a trend analysis over four triennia. Bmc Pregnancy and Childbirth. 2013;13.
65. McElwaine KM, Freund M, Campbell EM, Knight J, Bowman JA, Doherty EL, et al. The delivery of preventive care to clients of community health services. BMC health services research. 2013;13:167.
66. McFarlane K, Devine S, Judd J, Nichols N, Watt K. Workforce insights on how health promotion is practised in an Aboriginal Community Controlled Health Service. Australian journal of primary health. 2017;23(3):243-8.
67. Mu C, Hall J. What explains the regional variation in the use of general practitioners in Australia? BMC health services research. 2020;20(1):325.
68. Perkins D, Fuller J, Kelly BJ, Lewin TJ, Fitzgerald M, Coleman C, et al. Factors associated with reported service use for mental health problems by residents of rural and remote communities: cross-sectional findings from a baseline survey. BMC health services research. 2013;13:157.
69. Reeve C, Humphreys J, Wakerman J, Carter M, Carroll V, Reeve D. Strengthening primary health care: achieving health gains in a remote region of Australia. The Medical journal of Australia. 2015;202(9):483-7.
70. Yang B, Messom R. Association between potential primary care emergency service and general practitioner care utilisation in New South Wales. Emergency medicine Australasia : EMA. 2021;33(1):52-7.
71. Yelland J, Weetra D, Stuart-Butler D, Deverix J, Leane C, Ah Kit J, et al. Primary health care for Aboriginal women and children in the year after birth: findings from a population-based study in South Australia. Australian and New Zealand journal of public health. 2016;40(5):418-23.
72. Hegarty K, Parker R, Newton D, Forrest L, Seymour J, Sanci L. Feasibility and acceptability of nurse-led youth clinics in Australian general practice. Australian journal of primary health. 2013;19(2):159-65.
73. McMurray A, Ward L, Johnston K, Yang L, Connor M. The primary health care nurse of the future: Preliminary evaluation of the Nurse Navigator role in integrated care. Collegian. 2018;25(5):517-24.
74. Munns A. Community midwifery: A primary health care approach to care during pregnancy for Aboriginal and Torres Strait Islander women. Australian Journal of Primary Health. 2021;27(1):57-61.

75. Munns A, Mahony A, Miller K, Whitehead A. The WA Goldfields Aboriginal Community Antenatal Program-A community midwifery initiative. Collegian. 2016;23(4):367-72.
76. Reeve C, Humphreys J, Wakerman J, Carroll V, Carter M, O'Brien T, et al. Community participation in health service reform: the development of an innovative remote Aboriginal primary health-care service. Australian journal of primary health. 2015;21(4):409-16.
77. Ziersch A, Freeman T, Javanparast S, Mackean T, Baum F. Regional primary health care organisations and migrant and refugee health: the importance of prioritisation, funding, collaboration and engagement. Australian and New Zealand journal of public health. 2020;44(2):152-9.
78. Bentley M, Freeman T, Baum F, Javanparast S. Interprofessional teamwork in comprehensive primary healthcare services: Findings from a mixed methods study. Journal of interprofessional care. 2018;32(3):274-83.
79. James S, Halcomb E, Desborough J, McInnes S. Barriers and facilitators to lifestyle risk communication by Australian general practice nurses. Australian Journal of Primary Health. 2021;27(1):30-5.
80. Reath J, Abbott P, Kurti L, Morgan R, Martin M, Parry A, et al. Supporting aboriginal and Torres Strait islander cultural educators and cultural mentors in Australian general practice education. BMC medical education. 2018;18(1):236.
81. Loban E, Scott C, Lewis V, Law S, Haggerty J. Activating Partnership Assets to Produce Synergy in Primary Health Care: A Mixed Methods Study. Healthcare. 2021;9(8).
82. Bailie J, Matthews V, Laycock A, Schultz R, Burgess CP, Peiris D, et al. Improving preventive health care in Aboriginal and Torres Strait Islander primary care settings. Globalization and health. 2017;13(1):48.
83. Carman R, Andrew L, Devine A, Oosthuizen J. Barriers to vaccination service delivery within general practice: opportunity to make a sustainable difference in Aboriginal child health? Australian and New Zealand journal of public health. 2019;43(6):563-9.
84. Hegney DG, Patterson E, Eley DS, Mahomed R, Young J. The feasibility, acceptability and sustainability of nurse-led chronic disease management in Australian general practice: the perspectives of key stakeholders. International journal of nursing practice. 2013;19(1):54-9.
85. Hoang H, Le Q, Terry D. Women's access needs in maternity care in rural Tasmania, Australia: A mixed methods study. Women and Birth. 2014;27(1):9-14.
86. Lam L, Ansari AS, Baquir PJ, Chowdhury N, Tran K, Bailey J. Current practices, barriers and enablers for advance care planning among healthcare workers of aged care facilities in western New South Wales, Australia. Rural and Remote Health. 2018;18(4).
87. Robinson S, Varhol R, Ramamurthy V, Denehy M, Hendrie D, O'Leary P, et al. The Australian primary healthcare experiment: a national survey of Medicare Locals. BMJ open. 2015;5(3):e007191.
88. Swami M, Scott A. Impact of rural workforce incentives on access to GP services in underserved areas: Evidence from a natural experiment. Social science & medicine (1982). 2021;281:114045.
89. Zander KK, Taylor AJ, Carson DB. Impacts of Service and Infrastructure Provision on Indigenous Temporary Mobility in the Northern Territory of Australia: Insights from the 2011 Census. Population Space and Place. 2016;22(1):99-116.
90. Bartlem K, Wolfenden L, Colyvas K, Campbell L, Freund M, Doherty E, et al. The association between the receipt of primary care clinician provision of preventive care and short term health behaviour change. Preventive medicine. 2019;123:308-15.
91. Bloomfield JG, Gordon CJ, Williams AM, Aggar C. Nursing students' intentions to enter primary health care as a career option: Findings from a national survey. Collegian. 2015;22(2):161-7.
92. Keane S, Lincoln M, Rolfe M, Smith T. Retention of the rural allied health workforce in New South Wales: a comparison of public and private practitioners. Bmc Health Services Research. 2013;13.

93. Mazumdar S, Feng X, Konings P, McRae I, Girosi F. A brief report on Primary Care Service Area catchment geographies in New South Wales Australia. International journal of health geographics. 2014;13:38.
94. Morell AL, Kiem S, Millsteed MA, Pollice A. Attraction, recruitment and distribution of health professionals in rural and remote Australia: early results of the Rural Health Professionals Program. Human Resources for Health. 2014;12.
95. Quach J, Oberklaid F, Gold L, Lucas N, Mensah FK, Wake M. Primary health-care costs associated with special health care needs up to age 7 years: Australian population-based study. Journal of paediatrics and child health. 2014;50(10):768-74.
96. Zurynski Y, Ansell J, Ellis LA, Pomare C, Smith CL, Holt J, et al. Accessible and affordable healthcare? Views of Australians with and without chronic conditions. Internal medicine journal. 2021;51(7):1060-7.
97. Khanam MA, Kitsos A, Stankovich J, Kinsman L, Wimmer B, Castelino R, et al. Chronic kidney disease monitoring in Australian general practice. Australian journal of general practice. 2019;48(3):132-7.
98. Doyle J, Atkinson-Briggs S, Atkinson P, Firebrace B, Calleja J, Reilly R, et al. A prospective evaluation of first people's health promotion program design in the goulburn-murray rivers region. Bmc Health Services Research. 2016;16.
99. Inacio MC, Amare AT, Whitehead C, Bray SCE, Corlis M, Visvanathan R, et al. Factors associated with accessing aged care services in Australia after approval for services: Findings from the historical cohort of the Registry of Senior Australians. Australasian journal on ageing. 2020;39(3):e382-e92.
100. Tennett D, Kearney L, Kynn M. Access and outcomes of general practitioner obstetrician (rural generalist)-supported birthing units in Queensland. The Australian journal of rural health. 2020;28(1):42-50.
101. Thomas SL, Zhao Y, Guthridge SL, Wakerman J. The cost-effectiveness of primary care for Indigenous Australians with diabetes living in remote Northern Territory communities. The Medical journal of Australia. 2014;200(11):658-62.
102. Wood A, MacKay D, Fitzsimmons D, Derkenne R, Kirkham R, Boyle JA, et al. Primary Health Care for Aboriginal Australian Women in Remote Communities after a Pregnancy with Hyperglycaemia. International journal of environmental research and public health. 2020;17(3).
103. Valery PC, Bernardes CM, de Witt A, Martin J, Walpole E, Garvey G, et al. Patterns of primary health care service use of Indigenous Australians diagnosed with cancer. Supportive care in cancer : official journal of the Multinational Association of Supportive Care in Cancer. 2020;28(1):317-27.
104. Hussain J, Robinson A, Stebbing M, McGrail M. More is more in remote Central Australia: more provision of primary healthcare services is associated with more acute medical evacuations and more remote telephone consultations. Rural and remote health. 2014;14(4):2796.
105. Jones MP, Zhao Y, Guthridge S, Russell DJ, Ramjan M, Humphreys JS, et al. Effects of turnover and stability of health staff on quality of care in remote communities of the Northern Territory, Australia: a retrospective cohort study. BMJ open. 2021;11(10):e055635.
106. Sinclair C, Gates K, Evans S, Auret KA. Factors Influencing Australian General Practitioners' Clinical Decisions Regarding Advance Care Planning: A Factorial Survey. Journal of pain and symptom management. 2016;51(4):718-27.e2.
107. Khalil H, Lee S. The implementation of a successful medication safety program in a primary care. Journal of evaluation in clinical practice. 2018;24(2):403-7.
108. John JR, Tannous WK, Jones A. Effectiveness of a patient-centered medical home model of primary care versus standard care on blood pressure outcomes among hypertensive patients. Hypertension Research. 2020;43(9):892-902.
109. John JR, Tannous WK, Jones A. Outcomes of a 12-month patient-centred medical home model in improving patient activation and self-management behaviours among primary care

patients presenting with chronic diseases in Sydney, Australia: a before-and-after study. BMC family practice. 2020;21(1):158.
110. John JR, Tannous WK, Jones A. Effectiveness of a Patient-Centre Medical Home model on diabetes and other clinically relevant outcomes among primary care patients diagnosed with type-2 diabetes in Sydney, Australia. Primary care diabetes. 2021;15(3):464-71.
111. John JR, Tannous WK, Jones A. Changes in health-related quality of life before and after a 12-month enhanced primary care model among chronically ill primary care patients in Australia. Health and quality of life outcomes. 2020;18(1):288.
112. Percival N, O'Donoghue L, Lin V, Tsey K, Bailie RS. Improving Health Promotion Using Quality Improvement Techniques in Australian Indigenous Primary Health Care. Frontiers in Public Health. 2016;4.
113. Sawyer MG, Frost L, Bowering K, Lynch J. Effectiveness of nurse home-visiting for disadvantaged families: results of a natural experiment. BMJ open. 2013;3(4).
114. Harris MF, Parker SM, Litt J, van Driel M, Russell G, Mazza D, et al. An Australian general practice based strategy to improve chronic disease prevention, and its impact on patient reported outcomes: evaluation of the preventive evidence into practice cluster randomised controlled trial. BMC health services research. 2017;17(1):637.
115. Forster DA, McLachlan HL, Davey MA, Biro MA, Farrell T, Gold L, et al. Continuity of care by a primary midwife (caseload midwifery) increases women's satisfaction with antenatal, intrapartum and postpartum care: results from the COSMOS randomised controlled trial. BMC pregnancy and childbirth. 2016;16:28.
116. Liaw ST, Wade V, Furler JS, Hasan I, Lau P, Kelaher M, et al. Cultural respect in general practice: a cluster randomised controlled trial. The Medical journal of Australia. 2019;210(6):263-8.
117. Bartlem KM, Bowman J, Freund M, Wye PM, Barker D, McElwaine KM, et al. Effectiveness of an intervention in increasing the provision of preventive care by community mental health services: a non-randomized, multiple baseline implementation trial. Implementation science : IS. 2016;11:46.
118. Young CE, Janamian T, Beaver KM, Johnson JLK, Hannan-Jones C, Mutch AJ. Factors affecting the embedding of integrated primary-secondary care into a health district. Australian Journal of Primary Health. 2020;26(3):216-21.
119. Hulme Chambers A, Tomnay J, Stephens K, Crouch A, Whiteside M, Love P, et al. Facilitators of community participation in an Aboriginal sexual health promotion initiative. Rural and remote health. 2018;18(2):4245.
120. Odgers-Jewell K, Isenring E, Thomas R, Reidlinger DP. Process evaluation of a patient-centred, patient-directed, group-based education program for the management of type 2 diabetes mellitus. Nutrition & Dietetics. 2017;74(3):243-52.
121. Thomas S, Higgins H, Leask J, Menning L, Habersaat K, Massey P, et al. Improving child immunisation rates in a disadvantaged community in New South Wales, Australia: A process evaluation for research translation. Australian Journal of Primary Health. 2019;25(4):310-6.
122. Dasgupta P, Condon JR, Whop LJ, Aitken JF, Garvey G, Wenitong M, et al. Access to Aboriginal Community-Controlled Primary Health Organizations Can Explain Some of the Higher Pap Test Participation Among Aboriginal and Torres Strait Islander Women in North Queensland, Australia. Frontiers in Oncology. 2021;11.
123. Brown SJ, Gartland D, Weetra D, Leane C, Francis T, Mitchell A, et al. Health care experiences and birth outcomes: Results of an Aboriginal birth cohort. Women and Birth. 2019;32(5):404-11.
124. Larkins S, Woods CE, Matthews V, Thompson SC, Schierhout G, Mitropoulos M, et al. Responses of Aboriginal and Torres Strait Islander Primary Health-Care Services to Continuous Quality Improvement Initiatives. Frontiers in Public Health. 2016;3.
125. Ryan SM, Toumbourou JW, Jorm AF. Factors Associated With Service Use for Young Adolescents With Mental Health Problems: Findings From an Australian Longitudinal Study. Sage Open. 2014;4(4).

126. Isaacs A, Beauchamp A, Sutton K, Kocaali N. Care Coordination Can Reduce Unmet Needs of Persons With Severe and Persistent Mental Illness. Frontiers in Psychiatry. 2019;10.
127. Rolfe MI, Donoghue DA, Longman JM, Pilcher J, Kildea S, Kruske S, et al. The distribution of maternity services across rural and remote Australia: does it reflect population need? Bmc Health Services Research. 2017;17.
128. Slimings C, Moore M. Geographic variation in health system performance in rural areas of New South Wales, Australia. The Australian journal of rural health. 2021;29(1):41-51.
129. Wiggers J, McElwaine K, Freund M, Campbell L, Bowman J, Wye P, et al. Increasing the provision of preventive care by community healthcare services: a stepped wedge implementation trial. Implementation science : IS. 2017;12(1):105.
130. Baum F, Freeman T, Jolley G, Lawless A, Bentley M, Värttö K, et al. Health promotion in Australian multi-disciplinary primary health care services: case studies from South Australia and the Northern Territory. Health promotion international. 2014;29(4):705-19.
131. Callander E, Larkins S, Corscadden L. Variations in out-of-pocket costs for primary care services across Australia: a regional analysis. Australian journal of primary health. 2017;23(4):379-85.
132. Reifels L, Bassilios B, Nicholas A, Fletcher J, King K, Ewen S, et al. Improving access to primary mental healthcare for Indigenous Australians. The Australian and New Zealand journal of psychiatry. 2015;49(2):118-28.
133. Spaeth BA, Kaambwa B, Shephard MDS, Omond R. Economic evaluation of point-of-care testing in the remote primary health care setting of Australia’s Northern Territory. ClinicoEconomics and Outcomes Research. 2018;10:269-77.
134. Haggerty JL, Beaulieu M-D, Pineault R, Burge F, Lévesque J-F, Santor DA, et al. Comprehensiveness of care from the patient perspective: comparison of primary healthcare evaluation instruments. Healthc Policy. 2011;7(Spec Issue):154-66.
135. Kruske S, Kildea S, Jenkinson B, Pilcher J, Robin S, Rolfe M, et al. Primary Maternity Units in rural and remote Australia: Results of a national survey. Midwifery. 2016;40:1-9.
136. Goonetilleke M. Global Health Systems: Design and Reform. Handbook of Global Health: Springer; 2021. p. 1541-68.
137. Nicholson C, Jackson C, Marley J. A framework for integrated primary/secondary health care governance in Australia: results of a systematic review. APHCRI Centre of Research Excellence in Primary Health Care Microsystems, The University of Queensland. 2014.
138. Bath J, Wakerman J. Impact of community participation in primary health care: what is the evidence? Australian journal of primary health. 2015;21(1):2-8.
139. Thomas SL, Wakerman J, Humphreys JS. Ensuring equity of access to primary health care in rural and remote Australia - what core services should be locally available? Int J Equity Health. 2015;14:111.
140. Sturmberg JP. A Complex Adaptive Health System Redesign Based on “First Principles”. Health System Redesign: Springer; 2018. p. 75-96.
141. Senderowicz L, Pearson E, Hackett K, Huber-Krum S, Francis JM, Ulenga N, et al. ‘I haven’t heard much about other methods’: quality of care and person-centredness in a programme to promote the postpartum intrauterine device in Tanzania. BMJ Global Health. 2021;6(6):e005775.
142. Carey TA, Wakerman J, Humphreys JS, Buykx P, Lindeman M. What primary health care services should residents of rural and remote Australia be able to access? A systematic review of “core” primary health care services. BMC Health Services Research. 2013;13(1):178.
